# Gold(I) Complexes of ImPyDippDipp and ImPyMesMes: Biaryl L‑Shaped N‑Heterocyclic Carbene Analogues of IPr and IMes

**DOI:** 10.1021/acs.organomet.5c00093

**Published:** 2025-05-15

**Authors:** Yuzhuo Sha, Wenchao Chu, Tongliang Zhou, Roger Lalancette, Roman Szostak, Michal Szostak

**Affiliations:** † Department of Chemistry, 242613 Rutgers University, 73 Warren Street, Newark, New Jersey 07102, United States; ‡ Department of Chemistry, 49572 Wroclaw University, F. Joliot-Curie 14, Wroclaw 50-383, Poland

## Abstract

Imidazol-2-ylidenes, IPr and IMes, represent by far the most important and widely utilized N-heterocyclic carbenes in organic synthesis and catalysis. Herein, we report the synthesis, catalytic activity, and structural and electronic characterization of ImPyDippDipp and ImPyMesMes, sterically bulky and easily accessible biaryl L-shaped N-heterocyclic carbene analogues of IPr and IMes. These ligands exploit the rigid imidazo­[1,5-*a*]­pyridin-3-ylidene architecture to merge the properties of the biaryl scaffold with the electron-rich characteristics of the carbene center. The catalytic activity is evaluated in the gold­(I)-catalyzed hydration of alkynes and cyclization N-propargylamides, two model reactions for π-activation of alkynes that have found broad application in organic synthesis. Structural and electronic evaluation indicates that biaryl L-shaped ImPyDippDipp and ImPyMesMes ligands are more sterically demanding and more electron σ-donating and π-accepting than the classical imidazol-2-ylidnes, IPr and IMes. Both of these L-shaped ligands show excellent catalytic activity in gold­(I)-catalyzed hydration of alkynes and cyclization of N-propargylamides compared to their imidazol-2-ylidene congeners, IPr and IMes. Considering the tremendous impact of imidazol-2-ylidenes in homogeneous catalysis, we anticipate that this class of biaryl L-shaped NHCs will be rapidly and widely adopted to complement IPr and IMes N-heterocyclic carbenes.

## Graphical abstract

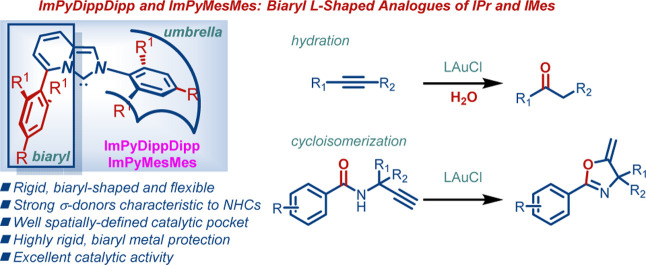

## Introduction

N-Heterocyclic carbenes (NHCs) have undergone rapid and extensive development in the last two decades, emerging as a central ligand class for a plethora of catalytic processes.^1,2^ Since the first isolation of a free carbene in 1991,^1,2^ studies on the development of new N-heterocyclic carbene ligands have primarily focused on (1) backbone modification, (2) N-wingtip modification, and (3) ring remodeling.^3,4^ In particular, by leveraging the flexible steric bulk of the N-aromatic wingtips, NHC ligands have enabled kinetic stabilization of various metals at different oxidation states, opening the door for their application in catalysis.^5^ Furthermore, the well-defined topology of N-heterocyclic carbenes has been widely utilized to fine-tune the reactivity of various metals and catalytic intermediates, where the sterically demanding N-heterocyclic carbenes have emerged as a privileged class of ligands for homogeneous catalysis.^6^ The pioneering contributions from Nolan,^7^ Glorius,^8^ Bertrand,^9^ and others^10^ have led to the discovery of various uniquely successful N-heterocyclic ligands, where the steric demand around the carbene center is the critical element of their high reactivity, including the broadly utilized carbenes, such as imidazol-2-ylidenes,^6^ cyclic (alkyl)­(amino)­carbenes (CAACs),^9^ sterically demanding bioxazoline carbenes (IBiox),^8^ and ring-expanded carbens,^12^ among other scaffolds (Figure 1A).^9–11^

**Figure 1:**
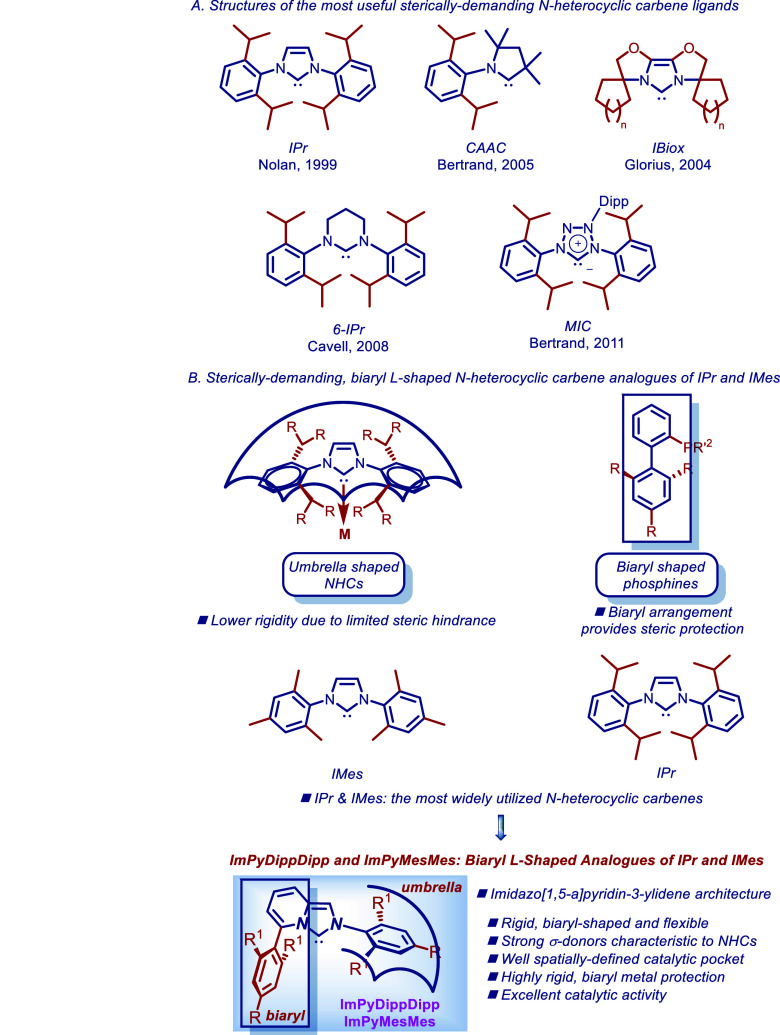
(A) Structures of the most useful sterically demanding N-heterocyclic carbene ligands. (B) Design of sterically demanding, biaryl L-shaped N-heterocyclic carbene analogous of IPr and IMes.

Inspired by our continued interest in N-heterocyclic carbenes and ligand development^13^ and being aware of the advances in NHC design^7–12^ and the tremendous success of biaryl-shaped phosphine ligands,^14^ we aimed to develop biaryl sterically hindered and strongly σ-electron-donating NHC ligands (Figure 1B).^3,4^ In general, the most broadly utilized imidazol-2-ylidenes, IPr and IMes, feature an umbrella-shaped architecture around the metal center, owing to the spatial distribution of N-aromatic wingtips. This in turn leads to lower rigidity, providing limited steric protection and restricting their potential scope of applications.^4^ In contrast, the vertically arranged biaryls have been among the most successful ligand design paradigms in the last decades, offering enhanced steric shielding and improved metal stability.^14^ Based on this concept and considering the highly appealing features of sterically restricted N-heterocyclic carbene ligands,^15^ herein, we report the synthesis, catalytic activity, and structural and electronic characterization of ImPyDippDipp and ImPyMesMes, sterically bulky and easily accessible biaryl N-heterocyclic carbene analogues of IPr and IMes (Figure 1B).^16^ Structural and electronic evaluation indicates that these biaryl L-shaped ImPyDippDipp and ImPyMesMes ligands are more sterically demanding, more electron σ-donating, and π-accepting than the classical imidazol-2-ylidnes, IPr and IMes. Both of these biaryl L-shaped ligands show excellent catalytic activity in gold­(I)-catalyzed hydration of alkynes^17^ and cyclization N-propargylamides^18^ compared to their imidazol-2-ylidene congeners, IPr and IMes. We anticipate that this class of biaryl L-shaped NHCs will find broad application to complement IPr and IMes N-heterocyclic carbenes.

## Results and Discussion

At the outset, ImPyDippDipp and ImPyMesMes were selected as the structural analogues of the most popular IPr and IMes imidazol-2-ylidene N-heterocyclic carbenes. The syntheses of ImPyDippDipp·HCl and ImPyMesMes·HCl are outlined in Scheme 1. Starting from the commercially available 6-bromopicolinaldehyde (**1**), 2-bromo-6-(1,3-dioxolan-2-yl)­pyridine (**2**) is obtained by the reaction with ethylene glycol in the presence of *p*TsOH and sodium sulfate. Next, Ni-catalyzed Kumada cross-coupling between (**2**) and the in situ prepared Grignard reagents (ArBr, magnesium, catalytic amount of I_2_) afforded biaryl intermediates **3**–**4** catalyzed by Ni­(PCy_3_)_2_Cl_2_. Condensation with 2,6-diisopropylaniline or 2,4,6-trimethylaniline in the presence of formaldehyde and HCl in dioxane afforded the desired ImPyDippDipp·HCl and ImPyMesMes·HCl salts in one pot. The desired products are readily obtained by trituration with diethyl ether/ethyl acetate as white solids, obviating the need for chromatographic purification. Note that the same route can be used for the synthesis of both ImPyDippDipp·HCl and ImPyMesMes·HCl, attesting to the generality of the approach.^13a^

**Scheme 1:**
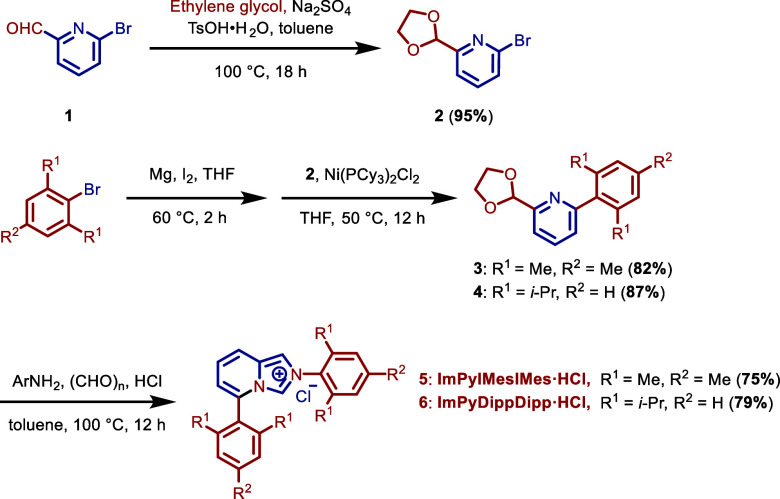
Synthesis of ImPyDippDipp·HCl and ImPyMesMes·HCl^a^

a Conditions: (a) 1 (1.0 equiv); ethylene glycol (2.0 equiv), Na_2_SO_4_ (1.0 equiv), TsOH·H_2_O (10 mol %), toluene, 100 °C, 12 h; (b) 1) Mg (1.44 equiv), I_2_, bromoarene (1.0 equiv), THF, 60 °C; 2 h, 2) 2 (1.0 equiv), Ni­(PCy_3_)_2_Cl_2_ (1 mol %), THF, 50 °C, 12 h; and (c) aniline (1.0 equiv), (CH_2_O)_n_ (1.5 equiv), 4 M HCl in dioxane, toluene, 100 °C, 12 h.

With access to ImPyMesMes·HCl (**5**) and ImPyDippDipp·HCl (**6**), we next comprehensively evaluated the steric properties of these biaryl L-shaped imidazo­[1,5-*a*]­pyridin-3-ylidene ligands. As shown in Scheme 2, gold­(I) complexes, [Au­(ImPyMesMes)­Cl] (**7**) and [Au­(ImPyDippDipp)­Cl] (**8**), were prepared using KO*t*Bu in THF.^19^ These Au­(I)–ImPy complexes (**7**) and (**8**) were found to be stable to air and moisture. Complexes (**7**) and (**8**) were characterized by X-ray crystallography (Figures 2 and 3).^20^ Previous studies by Cavallo, Nolan, and co-workers demonstrated that the combination of the % buried volume (% *V*_bur_) and steric maps of linear [Au­(NHC)­Cl] complexes are the best indication for quantifying the steric impact of NHC ligands.^4,21^ In our case, both [Au­(ImPyMesMes)­Cl] and [Au­(ImPyDippDipp)­Cl] are linear (**7**: C–Au–Cl, 178.10°; C–Au, 1.977 Å; **8**: C–Au–Cl, 179.3°; C–Au, 1.998 Å). The (% *V*_bur_) of [Au­(ImPyDippDipp)­Cl] is 48.8%, while the (% *V*_bur_) of [Au­(ImPyMesMes)­Cl] is 43.4%, indicating an increase in steric demand for the ImPyDippDipp ligand. These values can be compared with the (% *V*_bur_) of 40.3% and 34.4% determined for the analogous imidazol-2-ylidenes, [Au­(IPr)­Cl] and [Au­(IMes)­Cl, indicating a significant increase of steric contribution rendered by the vertical biaryl vs the umbrella-shaped N-wingtip.

**Scheme 2:**
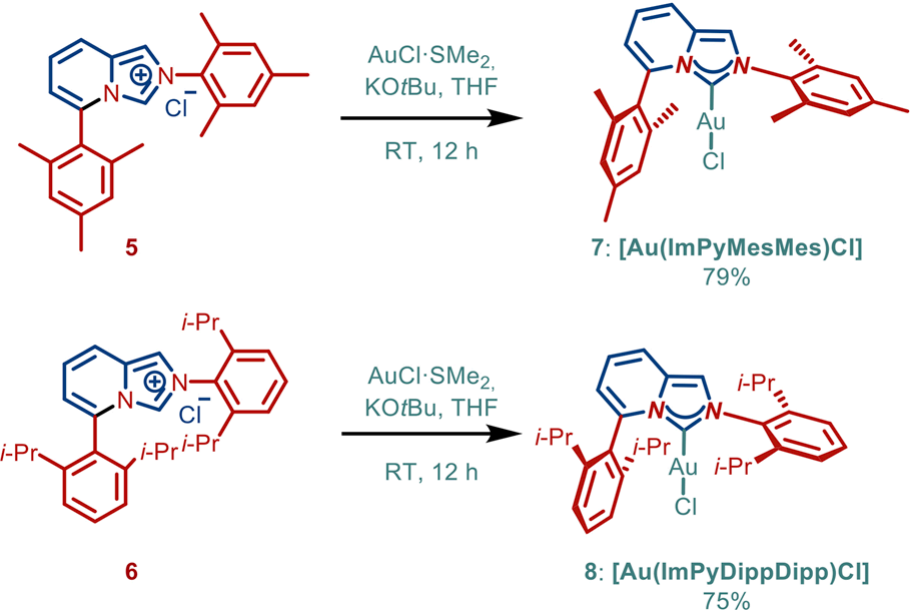
Synthesis of Au­(I)–ImPy Complexes^a^

a Conditions: (a) ImPy·HCl (1.0 equiv), AuCl·Me_2_S (1.0 equiv), KO*t*Bu (1.2 equiv), THF, RT, 12 h.

**Figure 2:**
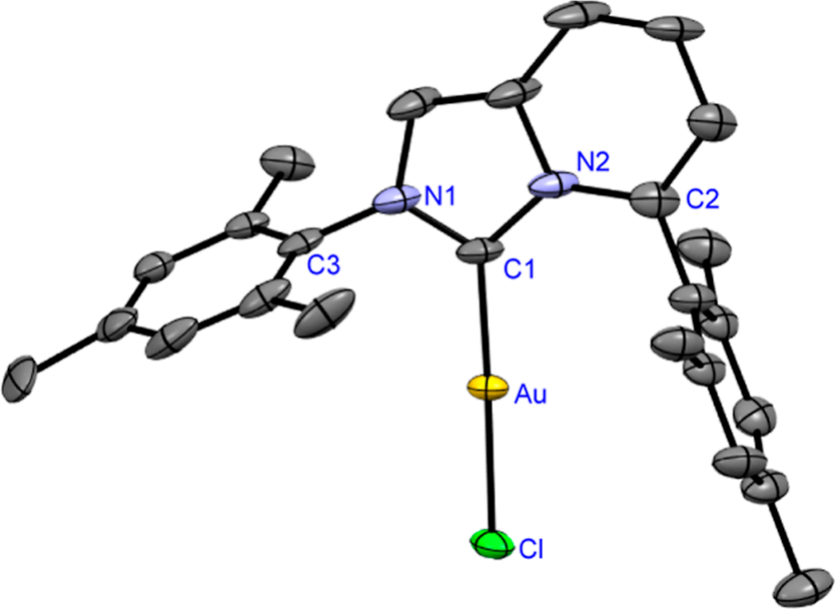
X-ray crystal structure of [Au­(ImPyMesMes)­Cl] (**7**). 50% ellipsoids. Hydrogen atoms have been omitted for the sake of clarity. Selected bond lengths (Å) and angles [°]: Au–C1, 2.278; Au–C1, 1.977; N1–C1, 1.354; N2–C1, 1.385; N2–C2, 1.401; N1–C3,1.436; C1–Au–Cl,178.06; N1–C1–N2, 104.24; C1–N2–C2,128.02; and C3–N1–C1,123.33. CCDC 2426013 (**7**).

**Figure 3:**
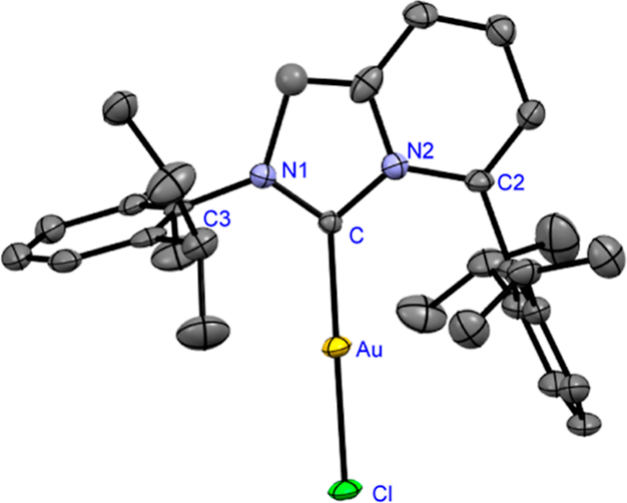
X-ray crystal structure of [Au­(ImPyDippDipp)­Cl] (**8**). 50% ellipsoids. Hydrogen atoms have been omitted for clarity. Selected bond lengths. Selected bond lengths [Å] and angles [°]: Au–C1, 2.299; Au–C1, 1.998; N1–C1, 1.333; N2–C1, 1.379; N2–C2, 1.395; N1–C3,1.450; C1–Au–Cl,179.31; N1–C1–N2, 104.75; C1–N2–C2,129.24; C3–N1–C1,124.53. CCDC 2426012 (**8**).

Importantly, the crystallographic analysis of [Au­(ImPyMesMes)­Cl] and [Au­(ImPyDippDipp)­Cl] revealed a spatially distinct, unsymmetrical biaryl quadrant distribution (**7**: SW 50.4%, NW 50.4%, NE 36.4%, SE 36.4%) and (**8**: SW 42.1%, NW 42.1%, NE 55.5%, SE 55.5%) for each quadrant (Figure 4). These values further indicate a unique enhancement of the steric impact of ImPyMesMes and ImPyDippDipp as compared to the classical IPr and IMes by the steric quadrant distribution: [Au­(IPr)­Cl] (SW 40.3%, NW 40.3%, NE 40.3%, SE 40.3%) and [Au­(IMes)­Cl] (SW 34.4%, NW 34.4%, NE 34.4%, SE 34.4%). This biaryl steric arrangement can provide an important steric differentiation of the biaryl fragment combined with the flexibility of the N-aromatic wingtip by merging vertical aryl and umbrella-wingtip topology.

**Figure 4:**
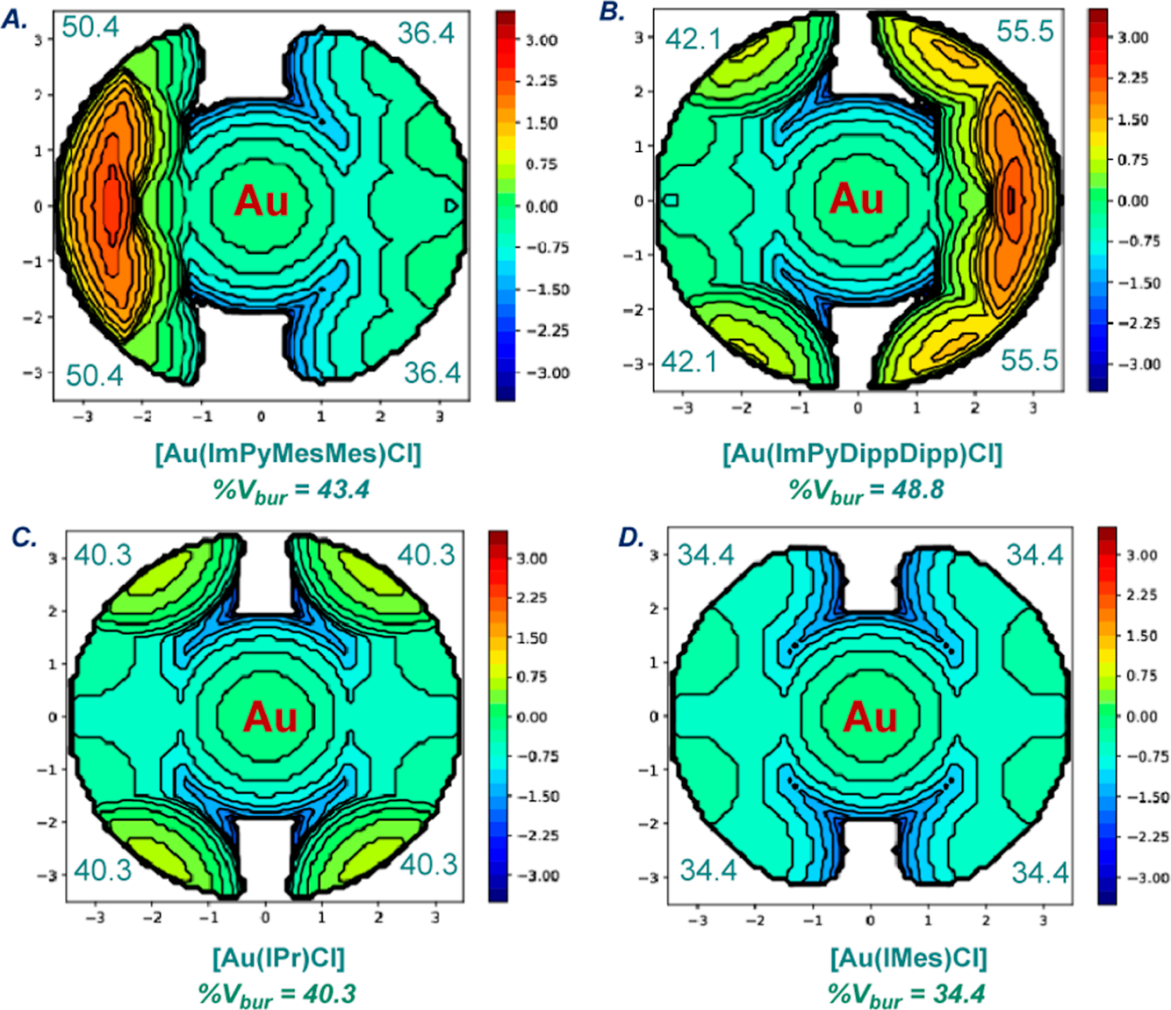
Topographical steric maps of part (A) [Au­(ImPyMesMes)­Cl] (**7**), (B) [Au­(ImPyDippDipp)­Cl] (**8**) and imidazol-2-ylidene congeners, (C) [Au­(IPr)­Cl], and (D) [Au­(IMes)­Cl], showing % *V*_bur_ per quadrant.

To gain insight into the electronic properties of these biaryl L-shaped ImPy N-heterocyclic carbenes, HOMO and LUMO energy levels were determined at the B3LYP 6-311++g­(d,p) level (Figure 5). The HOMO of ImPyDippDipp (−5.97 eV) is slightly higher than IPr (−6.01 eV), and the LUMO of ImPyDippDipp (−1.33 eV) is lower than IPr (−0.48 eV), indicating ImPyDippDipp is a stronger σ-donor and π-acceptor. Likewise, the HOMO of ImPyMesMes (−5.86 eV) is higher than that of IMes (−5.90 eV) and LUMO of ImPyMesMes (−1.24 eV) is lower than that of IMes (−0.33 eV). Furthermore, the HOMO values of [Au­(ImPyDippDipp)­Cl] (−5.90 eV) and of [Au­(ImPyMesMes)­Cl] (−5.84 eV) are higher than those of [Au­(IPr)­Cl] (−6.21 eV) and [Au­(IMes)­Cl] (−6.14 eV). Finally, the Wiberg bond orders of [Au­(ImPyDippDipp)­Cl] (Au–C_(carbene)_, 0.6224; Au–Cl, 0.5526) and [Au­(ImPyMesMes)­Cl] (Au–C_(carbene)_, 0.6459; Au–Cl, 0.5916) can be compared with [Au­(IPr)­Cl] (Au–C_(carbene)_, 0.6287; Au–Cl, 0.5504) and [Au­(IMes)­Cl] (Au–C_(carbene)_, 0.6607; Au–Cl, 0.6026), indicating comparatively strong Au–C_(carbene)_ bonds in the imidazo­[1,5-*a*]­pyridin-3-ylidene ligands. Thus, these biaryl L-shaped ImPy NHC ligands are characterized by strong σ-donation and π-acceptance in combination with vertical biaryl and flexible umbrella steric bulk, which makes these ImPy–NHC ligands well-suited for Au­(I) catalysis.

**Figure 5:**
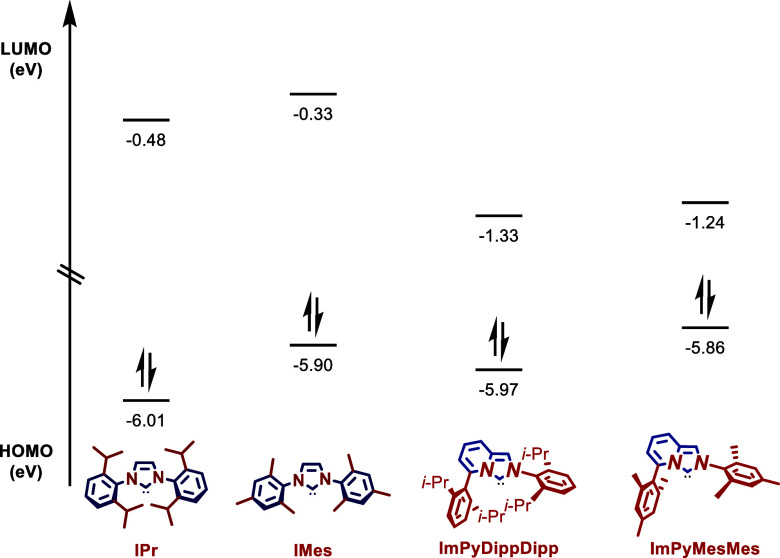
HOMO and LUMO energy levels (eV) of IPr, IMes, ImPyDippDipp, and ImPyMesMes. B3LYP 6-311++g­(d,p). See Supporting Information for details.

Next, we evaluated the catalytic activity of these biaryl L-shaped Au­(I)–ImPyNHC complexes (Schemes 3 and 4). Gold­(I)-catalyzed hydration of alkynes and cycloisomerization of N-propargylamides have been selected as model reactions owing to their importance in organic synthesis and catalysis as well as the well-defined effect of sterically demanding NHC ligands on these two reactions in that the reactions are ineffective in the absence of significant steric demand of the ligand. In particular, the seminal 2009 study by Nolan and co-workers demonstrated a remarkable effect of steric hindrance in Au­(I)–NHC-catalyzed hydration reactions in that [Au­(IPr)­Cl] promoted the reaction in high yields at 120 °C, while the less sterically demanding [Au­(IMes)­Cl] was completely unreactive.^22^

**Scheme 3:**
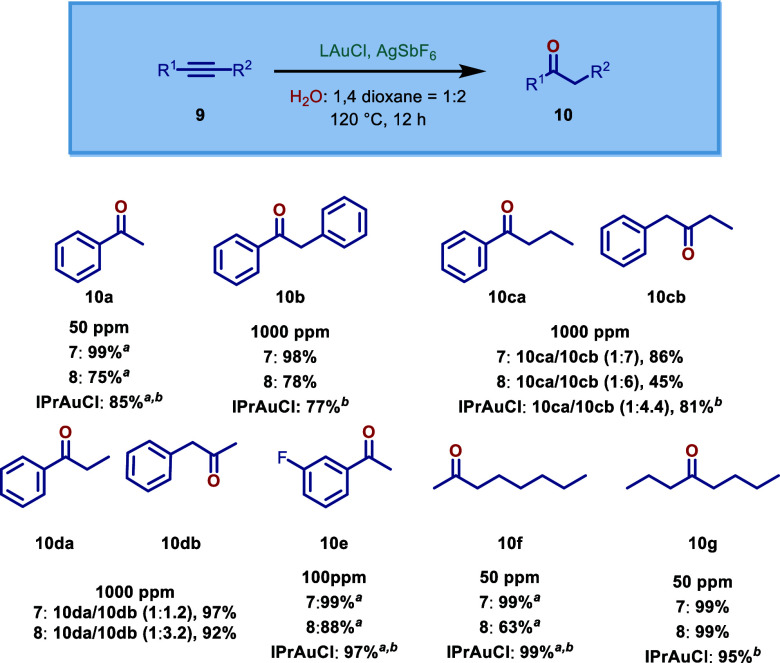
Au­(I)–Catalyzed Hydration of Alkynes Using [Au­(ImPyDippDipp)­Cl] and [Au­(ImPyMesMes)­Cl]^a^ ^,^ ^b^

a Solvent = MeOH.
b Ref 19. Note that IMesAuCl is unreactive.

**Scheme 4:**
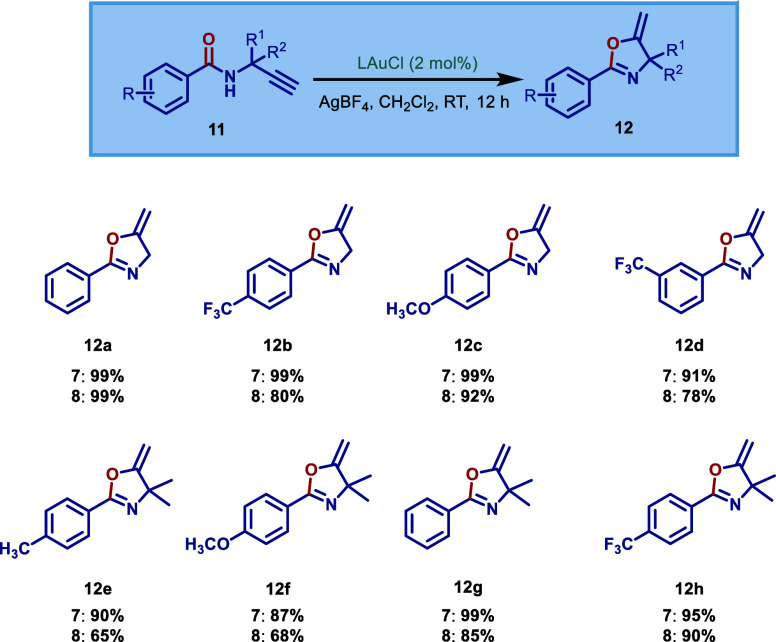
Au­(I)–Catalyzed Cycloisomerization of N-Propargylamides Using [Au­(ImPyDippDipp)­Cl] and [Au­(ImPyMesMes)­Cl]

Evaluation of the catalytic reactivity of Au­(I)–ImPyNHCs in hydration of alkynes revealed that both complexes [Au­(ImPyMesMes)­Cl] (**7**) and [Au­(ImPyDippDipp)­Cl] (**8**) showed excellent catalytic activity even at catalytic loading of 50 ppm (Scheme 3). The catalytic activity is illustrated through hydration of terminal aromatic and aliphatic alkynes and internal aliphatic and aromatic alkynes. Both complexes compare well with the more sterically demanding imidazol-2-ylidene counterparts [Au­(IPr)­Cl],^22^ while the more compact complex (**7**) is more reactive that the more sterically demanding ImPy complex (**8**). For example, the ImPyMesMes-based complex (**7**) afforded the product **10a** quantitatively at 50 ppm loading, while the ImPyDippDipp-based complex (**8**) gave 75% under the same conditions. In the hydration of 1-octyne, complex (**7**) afforded the product **10f** in a quantitative yield, while complex (**8**) was slightly less reactive at 50 ppm of catalytic loading. In the hydration of diphenylethylene and 4-octyne, complex (**7**) was still the best catalyst, affording 98% and 99% yield, respectively. In the hydration of internal unsymmetric alkynes, complex (**7**) afforded 86% yield and 1:7 α/β selectivity for **10ca** and **10cb** in the hydration of 1-phenyl-1-butyne and 97% yield and 1:2 α/β selectivity for **10da** and **10db** in the hydration of 1-phenyl-1-propyne. Thus, Au­(I)–ImPyMesMes emerges as one of the most reactive catalysts for the hydration of alkynes discovered to date.^22,13e^

We next evaluated the reactivity of these biaryl L-shaped ligands in gold­(I)-catalyzed cycloisomerization of N-propargylamides (Scheme 4).^23^ Previous studies demonstrated that similar to the hydration of alkynes, IMes-based catalysts are ineffective in this cycloisomerization. We selected this reaction owing to the utility of oxazolines in various areas of medicinal chemistry research. Preliminary control experiments demonstrated that [Au­(IPr)­Cl] afforded low yields of the model cyclization (<15% yield, 0.5 mol %), which can be compared with 80% yield obtained using [Au­(ImPyMesMes)­Cl] (**7**) and 50% yield using [Au­(ImPyDippDipp)­Cl] (**8**) under the same conditions. In general, both complexes [Au­(ImPyMesMes)­Cl] (**7**) and [Au­(ImPyDippDipp)­Cl] (**8**) show similar activity in this amide cycloisomerization. The less sterically demanding ImPyMesMes is slightly more reactive than the ImPyDippDipp-based catalyst. For example, complex (**7**) afforded a 90% yield of **12e**, while the yield using complex (**8**) was 65%. This cyclization proceeded smoothly with electron-deficient and electron-donating groups to afford **12b** (99%), **12d** (91%), **12c** (99%), and **12e** (90%). Furthermore, the complex [Au­(ImPyMesMes)­Cl] (**7**) was very active for the substrates containing two α-methyl groups (**12f**, 87%; **12g**, 99%; **12h**, 95%). Overall, the high steric demand of these biaryl, L-shaped ImPy ligands results in high activity in Au­(I)-catalyzed hydration and cycloisomerization reactions and bodes well for future applications of this family of ligands in Au­(I) catalysis as valuable counterparts of the classical IMes and IPr ligands.

## Conclusions

In summary, we have reported the synthesis, catalytic activity, and characterization of gold­(I) complexes of ImPyDippDipp and ImPyMesMes, two sterically demanding biaryl, L-shaped N-heterocyclic carbenes that are structural analogues of the two most popular imidazol-2-ylidenes, IPr and IMes. These ImPyNHC ligands feature vertically arranged biaryl architecture merged with a half-umbrella shaped N-wingtip that provides a well-defined catalytic pocket around the carbene center. The parent ligands are readily accessible in a simple, cost-effective, three synthetic steps. This straightforward and operationally simple synthetic pathway mirrors the ease of access to IPr and IMes and is one of the key requirements for broad adoption of new ligands by the synthetic community. Evaluation of structural and electronic properties provided insight into the steric impact, σ-donation, and π-accepting properties. These biaryl L-shaped ligands are more sterically demanding, more σ-donating, and π-accepting than the traditional imidazol-2-ylidenes. Both ligands showed excellent catalytic activity in the gold­(I)-catalyzed hydration of alkynes and cycloisomerization of N-propargylamides. The approach with vertical aryl arrangement in a rigid imidazo­[1,5-*a*]­pyridin-3-ylidene architecture provides an enticing avenue for the future development of novel sterically defined carbene ligands. Given the tremendous importance of N-heterocyclic carbenes in homogeneous catalysis, we expect that this class of biaryl ImPyNHCs will find widespread adoption as counterparts to the classical IPr and IMes.^24^

## Supplementary Material





## Notes

All synthetic procedures, NMR characterization of catalyst synthesis, hydration of alkynes and cyclization of N-proparglamides, crystallographic details, and computational details (PDF)
Cartesian coordinates with zero-point energies and thermal corrections (XYZ)
