# Supplementary material for: Gold(I) Complexes of ImPyDippDipp and ImPyMesMes: Biaryl L‑Shaped N‑Heterocyclic Carbene Analogues of IPr and IMes
Source: Organometallics. 2025 May 15;44(10):1100–7. doi: 10.1021/acs.organomet.5c00093 (PMC12333412; doi:10.1021/acs.organomet.5c00093)
Supplement: Supplementary file 1 [file om5c00093_si_001.pdf]

Supporting Information

Gold(I) Complexes of ImPyDippDipp and ImPyMesMes: Biaryl L-Shaped N-Heterocyclic Carbene Analogues of IPr and IMes

Yuzhuo Sha,[†] Wenchao Chu,[*,†] Tongliang Zhou,[†] Roger Lalancette,[†] Roman Szostak,[‡] and Michal Szostak[*,†]

[†]Department of Chemistry, Rutgers University, 73 Warren Street, Newark, New Jersey 07102, United States
[‡]Department of Chemistry, Wroclaw University, F. Joliot-Curie 14, Wroclaw 50-383, Poland
*Corresponding author:michal.szostak@rutgers.edu; wc558@scarletmail.rutgers.edu

Table of contents S1

General Information S2
General Procedure for Catalyst Synthesis S3
General Procedure for Hydration of Alkynes S6
General Procedure for Cyclization of N-Propargylamides S9
Crystallographic Details S12
Computational Details S16
^{1}H and ^{13}C NMR Spectra S17
References S43

General Information

All starting materials reported in the manuscript have been described in literature and prepared by the method reported previously unless stated otherwise. All substrates were were purchased from Oakwood or Alfa Aesar and used as received. N-Propargylamides were prepared from anilines and benzoyl chloride by standard methods. All experiments were performed using standard Schlenk techniques under nitrogen or argon unless stated otherwise. All solvents were purchased at the highest commercial grade and used as received or after purification by passing through activated alumina columns or distillation from sodium/benzophenone under nitrogen. All solvents were deoxygenated prior to use. All other chemicals were purchased at the highest commercial grade and used as received. Reaction glassware was oven-dried at 140 °C for at least 24 h or flame-dried prior to use, allowed to cool under vacuum and purged with argon (three cycles). All products were identified using ^{1}H NMR analysis and comparison with authentic samples. All yields refer to yields determined by ^{1}H NMR and/or GC or GC/MS using an internal standard (optimization) and isolated yields (preparative runs) unless stated otherwise. ^{1}H NMR, ^{13}C NMR and ^{19}F NMR spectra were recorded in $CDCl_3$ on Bruker spectrometers at 500 (^{1}H NMR), 125 MHz (^{13}C NMR) 471 MHz (^{19}F NMR). All shifts are reported in parts per million (ppm) relative to residual $CHCl_3$ peak (7.26 and 77.2 ppm, 1H NMR and ^{13}C NMR, respectively). All coupling constants (*J*) are reported in hertz (Hz). Abbreviations are: s, singlet; d, doublet; t, triplet; q, quartet; brs, broad singlet. GC-MS chromatography was performed using Agilent HP6890 GC System and Agilent 5973A inert XL EI/CI MSD using helium as the carrier gas at a flow rate of 1 mL/min and an initial oven temperature of 50 °C. The injector temperature was 250 °C. The detector temperature was 250 °C. For runs with the initial oven temperature of 50 °C, temperature was increased with a 10 °C/min ramp after 50 °C hold for 3 min to a final temperature of 220 °C, then hold at 220 °C for 15 min (splitless mode of injection, total run time of 22.0 min). High-resolution mass spectra (HRMS) were measured on a 7T Bruker Daltonics FT-MS instrument. All flash chromatography was performed using silica gel, 60 Å, 300 mesh. TLC analysis was carried out on glass plates coated with silica gel 60 F254, 0.2 mm thickness. The plates were visualized using a 254 nm ultraviolet lamp or aqueous potassium permanganate solutions. ^{1}H NMR and ^{13}C NMR data are given for all compounds in the SI for characterization purposes. ^{1}H NMR, ^{13}C NMR, ^{19}F NMR and HRMS data are given for all new compounds. All products have been previously reported, unless stated otherwise.

Experimental Procedures and Characterization Data

General Procedure for the Synthesis of ImPy–NHC Ligands

A. Synthesis of Imidazo[1,5-a]pyridin-2-ium Salts.

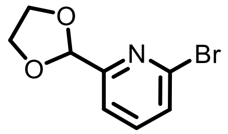

2-Bromo-6-(1,3-dioxolan-2-yl) pyridine 2.

A mixture of 6-bromopyridine-2-carboxaldehyde (18.6 g, 100 mmol, 1 equiv), ethylene glycol (11.2 mL, 12.4 g, 200 mmol, 2 equiv), *p*-toluenesulfonic acid monohydrate (1.72 g, 10 mmol, 10 mol%) and Na_2SO_4 (12 g, 100 mmol, 1 equiv) in toluene (100 mL) was heated at 100 °C for 12 h. Upon cooling, aqueous $NaHCO_3$ solution was added to the reaction mixture. The organic layer was separated, and the water layer was extracted with ethyl acetate (100 mL × 2). The combined organic layers were washed with brine and dried over Na_2SO_4. The solvent was removed in vacuo to give the product as colourless oil in 95% yield (21.9 g). ^{1}H NMR (500 MHz, $CDCl_3$) δ 7.51(t, *J* = 7.7 Hz, 1H), 7.40 (dd, *J* = 12.3, 7.7 Hz, 2H), 5.72 (s, 1H), 4.10 – 4.04 (m, 2H), 4.01 – 3.95(m, 2H). ^{13}C NMR (126 MHz, $CDCl_3$) δ 158.50, 141.54, 139.30, 128.48, 119.50, 102.69, 65.58. NMR spectroscopic data agreed with literature values.[1]

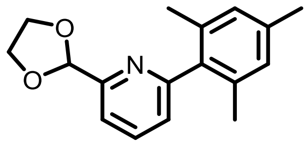

2-(1,3-Dioxolan-2-yl)-6-mesitylpyridine 3.

Activated magnesium turnings (173 mg, 7.2 mmol, 1.44 equiv) and a small amount of iodine (covering the tip of a spatula) was suspended in anhydrous THF (10 mL). To the mixture at ambient temperature was slowly added a solution of 2,4,6-trimethylbromobenzene (1.2g, 6 mmol, 1.2 equiv) in anhydrous THF (5 mL). After complete addition, the reaction mixture was heated at 60 °C for 2h. After indicated time, the mixture was cooled down to room temperature. To a well-stirred suspension of 2-bromo-6-(1,3-dioxolan-2-yl)pyridine (**2**) (1.15 g, 5 mmol, 1.0 equiv) and $Ni(PCy_3)Cl_2$ (34.5 mg, 0.05 mmol, 1 mol%) in anhydrous THF (10 mL) was slowly added the above Grignard solution over 10 minutes. The resultant brown solution was heated at 65 °C for 12 h, after which the mixture was poured over aqueous NH_4Cl solution. The aqueous layer was extracted with ethyl acetate, and the combined organic extracts were washed with brine and dried over anhydrous Na_2SO_4, filtered and concentrated in vacuo. The crude residue was purified by passing through a short pad of silica gel (hexane/ethyl acetate 10/1) and give colorless solid as product in 82% yield (1.1 g). ^{1}H NMR (500 MHz, $CDCl_3$) δ 7.72 (t, *J* = 7.7 Hz, 1H), 7.44 (dd, *J* = 7.8, 1.1 Hz, 1H), 7.15 (dd, *J* = 7.6, 1.1 Hz, 1H), 6.84 (s, 2H), 5.79 (s, 1H), 4.16 – 4.06 (m, 2H), 4.06 – 3.96 (m, 2H), 2.23 (s, 3H), 1.94 (s, 6H). ^{13}C NMR (126 MHz, $CDCl_3$) δ 159.40, 156.90,

137.52, 137.35, 137.04, 135.81, 128.34, 125.16, 118.44, 104.18, 65.60, 21.09, 20.23. NMR spectroscopic data agreed with literature values.[2]

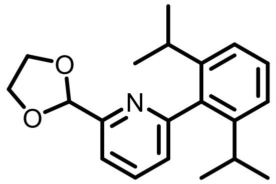

2-(2,6-Diisopropylphenyl)-6-(1,3-dioxolan-2-yl) pyridine 4.

Activated magnesium turnings (173 mg, 7.2 mmol, 1.44 equiv) and a small amount of iodine (covering the tip of a spatula) was suspended in anhydrous THF (10 mL). To the mixture at ambient temperature was slowly added a solution of 2,6-diisopropylbromobenzene (1.446g, 6 mmol, 1.2 equiv) in anhydrous THF (5 mL). After complete addition, the reaction mixture was heated at 60 °C for 2h. After indicated time, the mixture was cooled down to room temperature. To a well-stirred suspension of 2-bromo-6-(1,3-dioxolan-2-yl) pyridine (**2**) (1.15 g, 5 mmol, 1.0 equiv) and $Ni(PCy_3)Cl_2$ (34.5 mg, 0.05 mmol, 1 mol%) in anhydrous THF (10 mL) was slowly added the above Grignard solution over 10 minutes. The resultant brown solution was heated at 65 °C for 12 h, after which the mixture was poured over aqueous NH_4Cl solution. The aqueous layer was extracted with ethyl acetate, and the combined organic extracts were washed with brine and dried over anhydrous Na_2SO_4, filtered and concentrated in vacuo. The crude residue was purified by passing through a short pad of silica gel (hexane/ethyl acetate 10/1) and give pale yellow solid as product in 87% yield (1.3 g). ^{1}H NMR (500 MHz, $CDCl_3$) δ 7.73 (t, J = 7.7 Hz, 1H), 7.48 (dd, J = 7.8, 1.1 Hz, 1H), 7.31 – 7.25 (m, 1H), 7.21 – 7.16 (m, 1H), 7.14 (d, J = 7.8 Hz, 2H), 5.79 (s, 1H), 4.17 – 4.09 (m, 2H), 4.06 – 3.96 (m, 2H), 2.39 (hept, J = 6.8 Hz, 2H), 1.02 (dd, J = 21.2, 6.8 Hz, 12H). ^{13}C NMR (126 MHz, $CDCl_3$) δ 159.29, 156.63, 146.53, 138.39, 136.53, 128.59, 125.29, 122.69, 118.51, 104.14, 65.59, 30.34, 24.15, 23.91. NMR spectroscopic data agreed with literature values.[2]

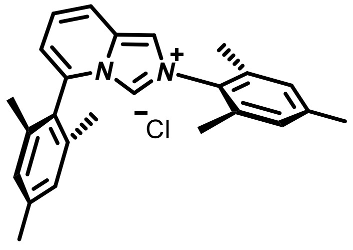

2,5-Dimesitylimidazo[1,5-a]pyridin-2-ium 5.

An oven-dried 100 mL round-bottomed flask equipped with a stir bar was charged with compound 3 (540 mg, 2.0 mmol, 1.0 equiv), 2,4,6-trimethylaniline (270 mg, 2 mmol, 1.0 equiv), paraformaldehyde (90 mg, 3.0 mmol, 1.5 equiv) and toluene (10 mL). The reaction mixture was stirred at 100 °C and 4 M HCl in dioxane (2 mL, 8.0 mmol, 4 equiv) was added. The resulting reaction mixture was stirred at 100 °C for 12 h. The title product was obtained by trituration from diethyl ether/ethyl acetate as colorless solid and give pale yellow solid as product in 75% yield

(545 mg). ^{1}H NMR (500 MHz, $CDCl_3$) δ 9.15 (s, 1H), 8.91 (d, *J* = 9.4 Hz, 1H), 7.79 (d, *J* = 1.9 Hz, 1H), 7.40 (dd, *J* = 9.4, 6.9 Hz, 1H), 6.99 (s, 2H), 6.96 (d, *J* = 6.9 Hz, 1H), 6.90 (s, 2H), 2.27 (d, *J* = 15.8 Hz, 6H), 1.95 (d, *J* = 17.0 Hz, 12H). ^{13}C NMR (126 MHz, $CDCl_3$) δ 141.45, 137.12, 134.05, 132.65, 132.45, 131.23, 129.70, 129.64, 126.76, 125.26, 121.72, 120.43, 119.94, 119.55, 21.35, 21.22, 19.24, 17.49. HRMS calcd for $C_{25}H_{27}N_2$ (M^+ - Cl) 355.2174, found 355.2170. Elemental analysis has not been performed. In this case, the NMR and HRMS data were used to confirm the structure.

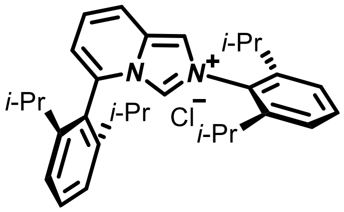

2,5-Bis(2,6-diisopropylphenyl)imidazo[1,5-a]pyridin-2-ium 6.

An oven-dried 100 mL round-bottomed flask equipped with a stir bar was charged with compound 4 (625 mg, 2.0 mmol, 1.0 equiv), 2,6-diisopropylaniline (354 mg, 2 mmol, 1.0 equiv), paraformaldehyde (90 mg, 3.0 mmol, 1.5 equiv) and toluene (10 mL). The reaction mixture was stirred at 100 °C and 4 M HCl in dioxane (2 mL, 8.0 mmol, 4 equiv) was added. The resulting reaction mixture was stirred at 100 °C for 12 h. The title product was obtained by trituration from diethyl ether/ethyl acetate as colorless solid and give pale yellow solid as product in 79% yield (740 mg). ^{1}H NMR (500 MHz, $CDCl_3$): δ 9.23 (s, 1H), 8.93 (t, *J* = 8.9 Hz, 1H), 7.85 (s, 1H), 7.55 – 7.44 (m, 3H), 7.29 (dd, *J* = 7.9, 1.1 Hz, 2H), 7.23 (dd, *J* = 7.9, 1.0 Hz, 2H), 7.04 (d, *J* = 6.8 Hz, 1H), 2.31 – 2.20 (m, 2H), 2.01 – 1.91 (m, 2H), 1.16 (dd, *J* = 6.6, 1.8 Hz, 6H), 1.11 (dd, *J* = 6.7, 1.3 Hz, 6H), 1.02 – 0.96 (m, 12H).^{13}C NMR (126 MHz, $CDCl_3$) δ 148.18, 144.96, 132.34, 130.38, 126.72, 125.74, 124.65, 124.54, 31.32, 28.82, 25.05, 24.64, 24.27, 23.82. HRMS calcd for $C_{31}H_{39}N_2$ $(M\text{-}Cl)^+$ 439.3108, found 439.3124. Elemental analysis has not been performed. In this case, the NMR and HRMS data were used to confirm the structure.

B. Synthesis of Au(I)–ImPyNHC Complexes.

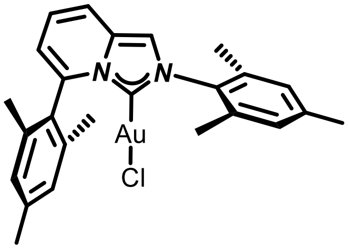

ImPyIMesIMesAuCl 7.

In a sealed reaction vial equipped with a magnetic stirring bar, 2,5-dimesitylimidazo[1,5-a]pyridin-2-ium **(5)** (58.8mg, 0.15mmol, 1 equiv) and AuCl·SMe_2 (45mg, 0.15mmol, 1 equiv) and potassium *tert*-butoxide (21mg, 0.18 mmol, 1.2 equiv) were stirred in THF at room temperature for 6 hours. Mixture was filtered through silica to get title product as colorless solid in 79% yield (70 mg). ^{1}H

NMR (500 MHz, $CDCl_3$) δ 7.37 (dd, *J* = 9.3, 1.3 Hz, 1H), 7.19 (s, 1H), 7.02 – 6.95 (m, 3H), 6.85 (s, 2H), 6.48 (dd, *J* = 6.6, 1.2 Hz, 1H), 2.34 (s, 3H), 2.24 (s, 3H), 2.00 (s, 6H), 1.89 (s, 6H). ^{13}C NMR (126 MHz, $CDCl_3$) δ 164.37, 140.83, 139.66, 138.61, 136.78, 136.13, 134.09, 131.55, 130.68, 129.31, 128.96, 123.48, 116.71, 116.22, 112.33, 21.46, 21.16, 19.90, 17.68. HRMS calcd for $C_{25}H_{30}N_3AuCl$ $(M+NH_4)^+$ 604.1794, found 604.1816.

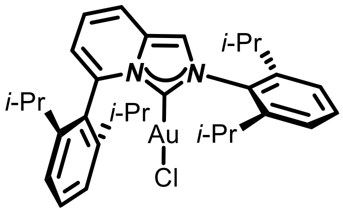

ImPyDippDippAuCl 8.

In a sealed reaction vial equipped with a magnetic stirring bar, 2,5-bis(2,6-diisopropylphenyl)imidazo[1,5-a]pyridin-2-ium **(6)** (71mg, 0.15mmol, 1 equiv) and $AuCl{\cdot}SMe_2$ (45mg, 0.15mmol, 1 equiv) and potassium *tert*-butoxide (21mg, 0.18 mmol, 1.2 equiv) were stirred in THF at room temperature for 6 hours. Mixture was filtered through silica to get title product as colorless solid in 75% yield (76 mg). ^1H NMR (500 MHz, $CDCl_3$) δ 7.55 (t, *J* = 7.8 Hz, 1H), 7.47 – 7.40 (m, 1H), 7.37 (t, *J* = 7.8 Hz, 1H), 7.27 (s, 1H), 7.24 (d, *J* = 7.9 Hz, 2H), 7.14 (d, *J* = 7.8 Hz, 2H), 7.03 (dd, *J* = 9.2, 6.6 Hz, 1H), 6.53 (dd, *J* = 6.6, 1.2 Hz, 1H), 2.35 (p, *J* = 6.8 Hz, 2H), 2.12 – 2.02 (m, 2H), 1.22 – 1.19 (m, 6H), 1.13 (d, *J* = 6.8 Hz, 6H), 1.06 (dd, *J* = 12.7, 6.8 Hz, 12H). ^{13}C NMR (126 MHz, $CDCl_3$) δ 166.09, 147.34, 145.01, 137.90, 135.51, 131.41, 130.96, 130.57, 124.08, 123.85, 123.36, 116.97, 116.91, 113.47, 31.76, 28.51, 25.21, 24.45, 24.21, 23.48. HRMS calcd for $C_{31}H_{39}N_2AuCl$ $(M+H)^+$ 671.2467, found 671.2457.

General Procedure for Hydration of Alkynes

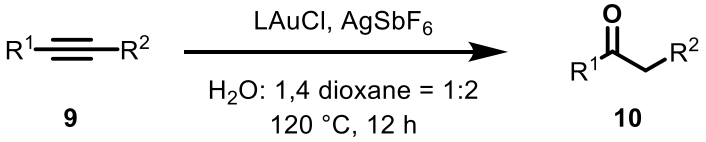

In a sealed reaction vial equipped with a magnetic stirring bar, alkynes (1 mmol, 1.0 equiv), Au-ImPy catalyst (50-1000 ppm), a small amount of $AgSbF_6$ (covering the tip of a spatula) was added. Solvent (0.6 mL) and water (0.3 mL) were added. The reaction was stirred at 120 °C for 12 hours. The residue was analysed by ^1H NMR ($CDCl_3$, 500 MHz) using internal standard. Purification by chromatography on silica gel (EtOAc/hexanes or DCM/MeOH) afforded the title product.

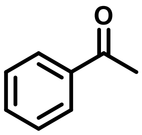

1-Phenylethan-1-one 10a.
According to the general procedure, using **7** (50 ppm), the reaction afforded compound **10a** in 99% yield (120.0 mg). Using **8** (50 ppm), the reaction afforded compound **10a** as colourless oil in 75% yield (90.0 mg). ^{1}H NMR (500 MHz, $CDCl_3$) 7.89 (d, *J* = 7.0 Hz, 2H), 7.51 – 7.47 (t, 1H), 7.42 – 7.37 (m, 2H), 2.53 (s, 3H). ^{13}C NMR (126 MHz, $CDCl_3$): δ 198.17, 137.15, 133.12, 128.58, 128.32 26.63. NMR spectroscopic data agreed with literature values.[3]

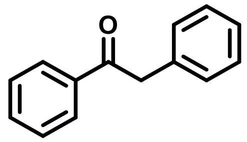

1,2-Diphenylethan-1-one 10b.
According to the general procedure, using **7** (1000 ppm), the reaction afforded compound **10b** in 99% yield (196.0 mg). Using **8** (1000 ppm), the reaction afforded compound **10b** as pale yellow solid in 77% yield (150.0 mg). ^{1}H NMR (500 MHz, $CDCl_3$) δ 7.94 – 7.92 (d, *J*=7.5Hz, 2H), 7.50 (t, *J* =7.0Hz, 1H), 7.40 (t, *J* =7.5Hz, 2H), 7.28 (t, *J* =7.5Hz, 2H), 7.21 (t, *J* =7.0Hz, 3H), 4.20 (s, 2H). ^{13}C NMR (126 MHz, $CDCl_3$) δ 197.66, 136.63, 134.57, 133.20, 129.50, 128.71, 128.68, 128.65, 126.92, 45.53. NMR spectroscopic data agreed with literature values.[4]

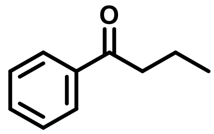

1-Phenylbutan-1-one 10ca.
According to the general procedure, using **7** (1000 ppm), the reaction afforded compound **10ca** in 11% yield (16 mg). Using **8** (1000 ppm), the reaction afforded compound **10ca** as colourless oil in 6.4% yield (10 mg). ^{1}H NMR (500 MHz, $CDCl_3$) δ 7.90 – 7.83 (m, 2H), 7.48 – 7.39 (m, 1H), 7.38 – 7.31 (m, 2H), 2.84 (t, *J* = 7.3 Hz, 2H), 1.67 (h, *J* = 7.3 Hz, 2H), 0.92 (q, *J* = 7.3 Hz, 3H). ^{13}C NMR (126 MHz, $CDCl_3$) δ 200.49, 137.11,132.91, 128.57, 128.06, 40.52, 17.79, 13.91. NMR spectroscopic data agreed with literature values.[3]

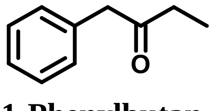

1-Phenylbutan-2-one 10cb.
According to the general procedure, using **7** (1000 ppm), the reaction afforded compound **10cb** in 74% yield (110.0 mg). Using **8** (1000 ppm), the reaction afforded compound **10cb** as colourless oil in 39% yield (54.0 mg). ^{1}H NMR (500 MHz, $CDCl_3$) ^{1}H NMR (500 MHz, CDCl3) δ 7.27 – 7.18 (m, 2H), 7.18 – 7.13 (m, 1H), 7.12 – 7.06 (m, 2H), 3.58 (s, *J* = 7.1 Hz, 2H), 2.37 (q, 2H), 0.94 (t, *J* = 7.3 Hz, 3H).^{13}C NMR (126 MHz, $CDCl_3$) 209.07, 134.51, 126.96, 128.71, 127.01, 49.80, 35.22, 7.79. NMR spectroscopic data agreed with literature values.[5]

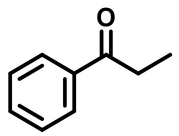

Propiophenone 10da.

According to the general procedure, using **7** (1000 ppm), the reaction afforded compound **10da** in 45% yield (60.0 mg). Using **8** (1000 ppm), the reaction afforded compound **10da** as colourless oil in 17% yield (22.0 mg). ^{1}H NMR (500 MHz, $CDCl_3$) δ 7.92 (d, *J* = 7.5 Hz, 2H), 7.50 (t, *J* = 7.5 Hz, 1H), 7.41(t, *J* = 8.0 Hz, 2H), 2.92 (q, *J* = 7.3 Hz, 2H), 1.15 (t, *J* = 7.2 Hz, 3H).^{13}C NMR (126 MHz, $CDCl_3$) δ 200.90, 136.94, 132.90, 128.58, 128.00, 31.80, 8.27. NMR spectroscopic data agreed with literature values.[3]

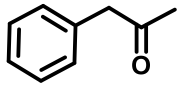

1-Phenylpropan-2-one 10db.

According to the general procedure, **7** using (1000 ppm), the reaction afforded compound **10db** in 47% yield (63.0 mg). Using **8** (1000 ppm), the reaction afforded compound **10db** as colourless oil in 53% yield (72.0 mg). ^{1}H NMR (500 MHz, $CDCl_3$) δ 7.30 (d, *J* = 7.5 Hz, 2H), 7.22 – 7.16 (t, *J* = 7.5 Hz, 1H), 7. (t, *J* = 8.0 Hz, 2H), 3.61 (s, 2H), 2.07 (s, 3H).^{13}C NMR (126 MHz, $CDCl_3$) δ 206.50, 134.27, 129.42, 128.78, 127.09, 51.04, 29.29. NMR spectroscopic data agreed with literature values.[5]

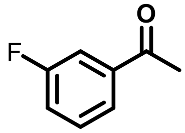

1-(3-Fluorophenyl)ethan-1-one 10e.

According to the general procedure, using **7** (100 ppm), the reaction afforded compound **10e** in 99% yield (138.0 mg). Using **8** (100 ppm), the reaction afforded compound **10e** as colourless oil in 88% yield (121.0 mg).^{1}H NMR (500 MHz, $CDCl_3$) δ 7.66 (dt, *J* = 7.7, 1.3 Hz, 1H), 7.56 (ddd, *J* = 9.5, 2.7, 1.6 Hz, 1H), 7.37 (td, *J* = 8.0, 5.5 Hz, 1H), 7.19 (tdd, *J* = 8.3, 2.6, 1.0 Hz, 1H), 2.52 (s, 3H).^{13}C NMR (126 MHz, $CDCl_3$) δ 195.79 (d, *J* = 2.1 Hz), 162.80, 160.83, 129.24 (d, *J* = 7.4 Hz), 123.11 (d, *J* = 2.8 Hz), 119.11 (d, *J* = 21.7 Hz), 113.92 (d, *J* = 22.2 Hz), 25.66. ^{19}F NMR (471 MHz, $CDCl_3$) δ -112.95. NMR spectroscopic data agreed with literature values.[3]

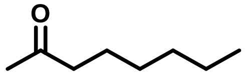

2-Octanone 10f.

According to the general procedure, using **7** (50 ppm), the reaction afforded compound **10f** in 99% yield (128.0 mg). Using **8** (50 ppm), the reaction afforded compound **10f** as colourless oil in 65% yield (81.0 mg). ^{1}H NMR (500 MHz, $CDCl_3$) δ 2.34 (t, *J* = 7.5 Hz, 2H), 2.05 (d, *J* = 2.0 Hz, 3H), 1.52 – 1.44 (m, 2H), 1.29 – 1.14 (m, 6H), 0.80 (t, *J* = 6.8 Hz, 3H). ^{13}C NMR (126 MHz, $CDCl_3$) δ 208.95, 170.84, 43.61, 31.49, 28.73, 23.69, 22.37, 13.85. NMR spectroscopic data agreed with literature values.[3]

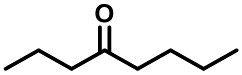

4-Octanone 10g.

According to the general procedure, using **7** (100 ppm), the reaction afforded compound **10g** in 99% yield (128.0 mg). Using **8** (100 ppm), the reaction afforded compound **10g** as colourless oil

in 98% yield (128.0 mg). δ 2.45 – 2.25 (m, 4H), 1.59 – 1.43 (m, 4H), 1.24 (d, *J* = 7.5 Hz, 2H), 0.80 (t, *J* = 6.8 Hz, 6H).^{13}C NMR (126 MHz, $CDCl_3$) δ 211.53, 44.67, 42.51, 25.94, 22.35, 17.28, 13.82, 13.73. NMR spectroscopic data agreed with literature values.[4]

General Procedure for Cyclization of N-Propargylamides

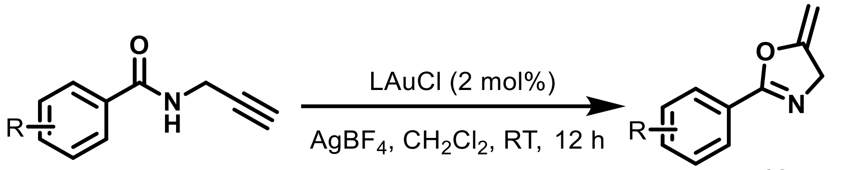

In a sealed reaction vial equipped with a magnetic stirring bar, Au-ImPy catalyst (2 mol%) was dissolved in CH_2Cl_2 (1 mL). A small amount of $AgBF_4$ (covering the tip of a spatula) was added and the reaction mixture was stirred for 1 minute. Propargyl amide (1 mmol, 1 equiv) was added. The reaction mixture was then stirred at room temperature for 12 hours. The residue was analysed by ^{1}H NMR ($CDCl_3$, 500 MHz) using internal standard. Purification by chromatography on silica gel (EtOAc/hexanes) afforded the title product.

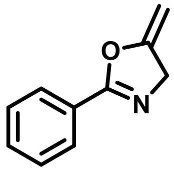

2-Phenyl-5-methylene-4,5-dihydrooxazole 12a.
According to the general procedure, using **7** (2 mol%), the reaction afforded compound **12a** in 99% yield (158.0 mg). Using **8** (2 mol%), the reaction afforded compound **12a** as colorless oil in 87% yield (137.0 mg). ^{1}H NMR (500 MHz, $CDCl_3$) δ 7.93 – 7.88 (dd, 2H), 7.47 – 7.41 (m, 1H), 7.37 (tt, *J* = 6.7, 1.5 Hz, 2H), 4.75 (q, *J* = 3.0 Hz, 1H), 4.58 (t, *J* = 2.9 Hz, 2H), 4.29 (q, *J* = 2.7 Hz, 1H). ^{13}C NMR (126 MHz, $CDCl_3$) δ 163.77, 158.83, 131.84, 128.51, 128.03, 126.74, 83.82, 57.73. NMR spectroscopic data agreed with literature values.[6]

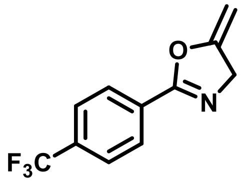

2-(4-(Trifluoromethyl)phenyl)-4,5-dihydro-5-methyleneoxazole 12b.
According to the general procedure, using **7** (2 mol%), the reaction afforded compound **12b** in 99% yield (226.0 mg). Using **8** (2 mol%), the reaction afforded compound **12b** as colorless solid in 83% yield (187.0 mg). ^{1}H NMR (500 MHz, $CDCl_3$) δ 8.03 (d, *J* = 8.2 Hz, 2H), 7.64 (d, *J* = 8.2 Hz, 2H), 4.78 (q, *J* = 3.0 Hz, 1H), 4.61 (t, *J* = 2.9 Hz, 2H), 4.34 (q, *J* = 2.8 Hz, 1H).^{13}C NMR (126 MHz, $CDCl_3$) δ 162.62, 158.47, 133.42(q, *J* = 32.7 Hz), 130.06, 128.89, 128.40, 125.52 (q, *J* = 3.8 Hz),

84.49, 57.81. ^{19}F NMR (471 MHz, $CDCl_3$) δ 63.06. NMR spectroscopic data agreed with literature values.[6]

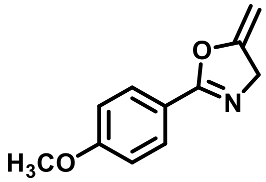

2-(4-Methoxyphenyl)-5-methylene-4,5-dihydrooxazole 12c.
According to the general procedure, using **7** (2 mol%), the reaction afforded compound **12c** in 99% yield (189.0 mg). Using **8** (2 mol%), the reaction afforded compound **12c** as colorless solid in 90% yield (170.0 mg). ^{1}H NMR (500 MHz, $CDCl_3$) δ 7.89 – 7.82 (m, 2H), 6.91 – 6.84 (m, 2H), 4.73 (q, *J* = 3.0 Hz, 1H), 4.56 (t, *J* = 2.8 Hz, 2H), 4.28 (q, *J* = 2.6 Hz, 1H), 3.79 (s, 3H). ^{13}C NMR (126 MHz, $CDCl_3$) δ 163.59, 162.52, 158.91, 129.85, 113.91, 83.55, 57.54, 55.41. NMR spectroscopic data agreed with literature values.[6]

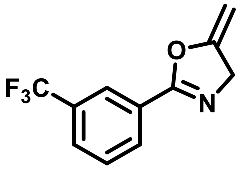

5-Methylene-2-(3-(trifluoromethyl) phenyl)-4,5-dihydrooxazole 12d.
According to the general procedure, using **7** (2 mol%), the reaction afforded compound **12d** in 90% yield (204.0 mg). Using **7** (2 mol%), the reaction afforded compound **12d** pale yellow solid in 78% yield (177.0 mg). ^{1}H NMR (500 MHz, $CDCl_3$) δ 8.16 (d, *J* = 1.8 Hz, 1H), 8.10 (dd, *J* = 7.7, 1.6 Hz, 1H), 7.71 – 7.67 (m, 1H), 7.51-7.47(m,1H), 4.81 (q, *J* = 3.1 Hz, 1H), 4.64 (t, *J* = 2.9 Hz, 2H), 4.37 (q, *J* = 2.8 Hz, 1H). ^{13}C NMR (126 MHz, $CDCl_3$) δ 162.60, 158.45, 132.06 – 130.72 (m), 129.15, 128.38 (q, *J* = 3.7 Hz), 127.61, 125.03 (q, *J* = 3.9 Hz), 84.57, 57.74, 11.07. ^{19}F NMR (471 MHz, $CDCl_3$) δ -62.87. HRMS calcd for $C_{11}H_8F_3NO$ 227.0558, found 227.0564. Elemental analysis has not been performed. In this case, the NMR and HRMS data were used to confirm the structure.

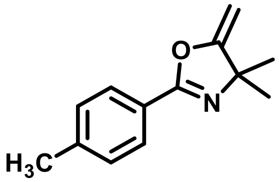

4,4-Dimethyl-5-methylene-2-(*p*-tolyl)-4,5-dihydrooxazole 12e.
According to the general procedure, using **7** (2 mol%), the reaction afforded compound **12e** in 90% yield (200.0 mg). Using **8** (2 mol%), the reaction afforded compound **12e** as colorless oil in 65% yield (130.0 mg). ^{1}H NMR (500 MHz, $CDCl_3$) δ 7.79 (d, *J* = 8.2 Hz, 2H), 7.15 (d, *J* = 8.0 Hz, 2H), 4.64 (d, *J* = 2.9 Hz, 1H), 4.15 (d, *J* = 2.9 Hz, 1H), 2.32 (s, 3H), 1.37 (s, 6H). ^{13}C NMR (126 MHz, $CDCl_3$) δ 168.02, 159.91, 142.09, 129.17, 128.07, 124.19, 82.08, 68.99, 29.79, 21.60. NMR spectroscopic data agreed with literature values.[7]

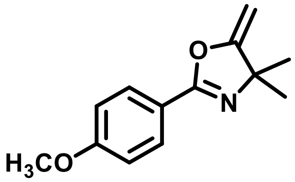

2-(4-Methoxyphenyl)-4,4-dimethyl-5-methylene-4,5-dihydrooxazole 12f.
According to the general procedure, using **7** (2 mol%), the reaction afforded compound **12f** in 87% yield (188.0 mg). Using **8** (2 mol%), the reaction afforded compound **12f** pale yellow solid in 60% yield (130.0 mg). ^{1}H NMR (500 MHz, CDCl3) δ 7.85 (d, *J* = 8.9 Hz, 2H), 6.85 (d, *J* = 8.9 Hz, 2H), 4.63 (d, *J* = 2.9 Hz, 1H), 4.15 (d, *J* = 2.8 Hz, 1H), 3.77 (s, 3H), 1.36 (s, 6H). ^{13}C NMR (126 MHz, $CDCl_3$) δ 168.07, 162.34, 159.61, 129.86, 119.40, 113.83, 81.96, 68.92, 55.38, 29.82. NMR spectroscopic data agreed with literature values.[7]

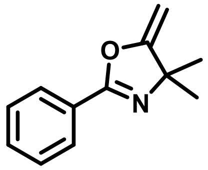

4,4-Dimethyl-5-methylene-2-phenyl-4,5-dihydrooxazole 12g.
According to the general procedure, using **7** (2 mol%), the reaction afforded compound **12g** in 99% yield (189.0 mg). Using **8** (2 mol%), the reaction afforded compound **12g** as colorless solid in 90% yield (170.0 mg). ^{1}H NMR (500 MHz, $CDCl_3$) δ 7.95 – 7.90 (m, 2H), 7.43 (d, *J* = 7.5 Hz, 1H), 7.39 – 7.33 (m, 2H), 4.67 (d, *J* = 2.9 Hz, 1H), 4.18 (d, *J* = 2.9 Hz, 1H), 1.39 (s, 6H).^{13}C NMR (126 MHz, $CDCl_3$) 168.00, 159.85, 131.63, 128.44, 128.12, 127.05, 82.26, 69.10, 29.75. NMR spectroscopic data agreed with literature values.[6]

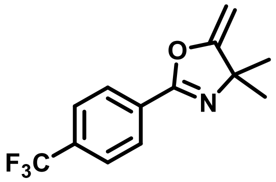

2-(4-(Trifluoromethyl)phenyl)-4,4-dimethyl-5-methylene-4,5-dihydrooxazole 12h.
According to the general procedure, using **7** (2 mol%), the reaction afforded compound **12h** in 99% yield (255.0 mg). Using **8** (2 mol%), the reaction afforded compound **12h** colorless oil in 99% yield (255.0 mg). ^{1}H NMR (500 MHz, $CDCl_3$) δ 8.04 (d, *J* = 8.1 Hz, 2H), 7.63 (d, *J* = 8.2 Hz, 2H), 4.70 (d, *J* = 3.1 Hz, 1H), 4.22 (d, *J* = 3.0 Hz, 1H), 1.39 (s, 6H).^{13}C NMR (126 MHz, $CDCl_3$) δ 167.58, 158.76, 133.42, 133.16, 130.38, 128.49, 125.46 (t, *J* = 3.7 Hz), 124.82, 122.65, 83.03, 69.40, 29.68. ^{19}F NMR (471 MHz, $CDCl_3$) δ -63.00. NMR spectroscopic data agreed with literature values.[7]

ORTEP Structures of 7 and 8

Figure S1. *ORTEP Structure of* **7** *(50% ellipsoids)*. (Crystallographic data has been deposited with the Cambridge Crystallographic Data Center as supplementary publication no. CCDC 2426013).

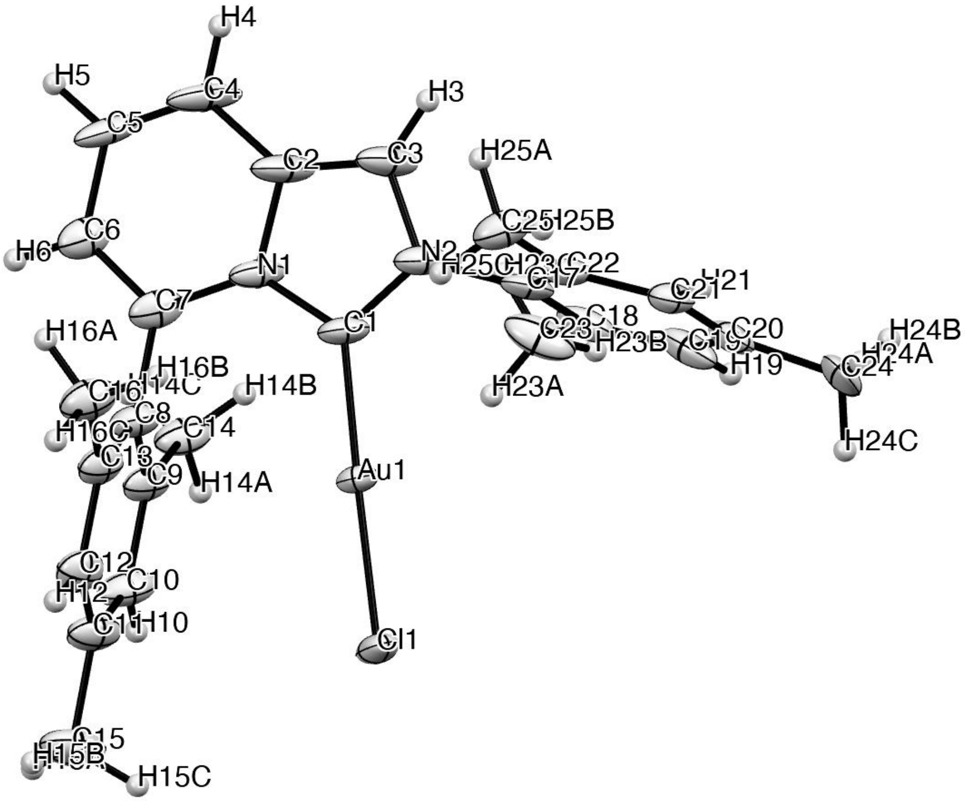

Selected bond lengths [Å] and angles [°]: Au–C1, 1.998; Au–Cl, 2.299; C1–N1, 1.333; C1–N2, 1.379; C2–C3, 1.223; C1–Au–Cl, 179.3; Au–C1–N2, 127.5; Au–C1–N1, 127.75; N1–C1–N2, 104.8; C1–N1–C2, 108.8; C3–N2–C1, 111.2.

Figure S2. *ORTEP Structure of* **8** *(50% ellipsoids).* (Crystallographic data has been deposited with the Cambridge Crystallographic Data Center as supplementary publication no. CCDC 2426012).

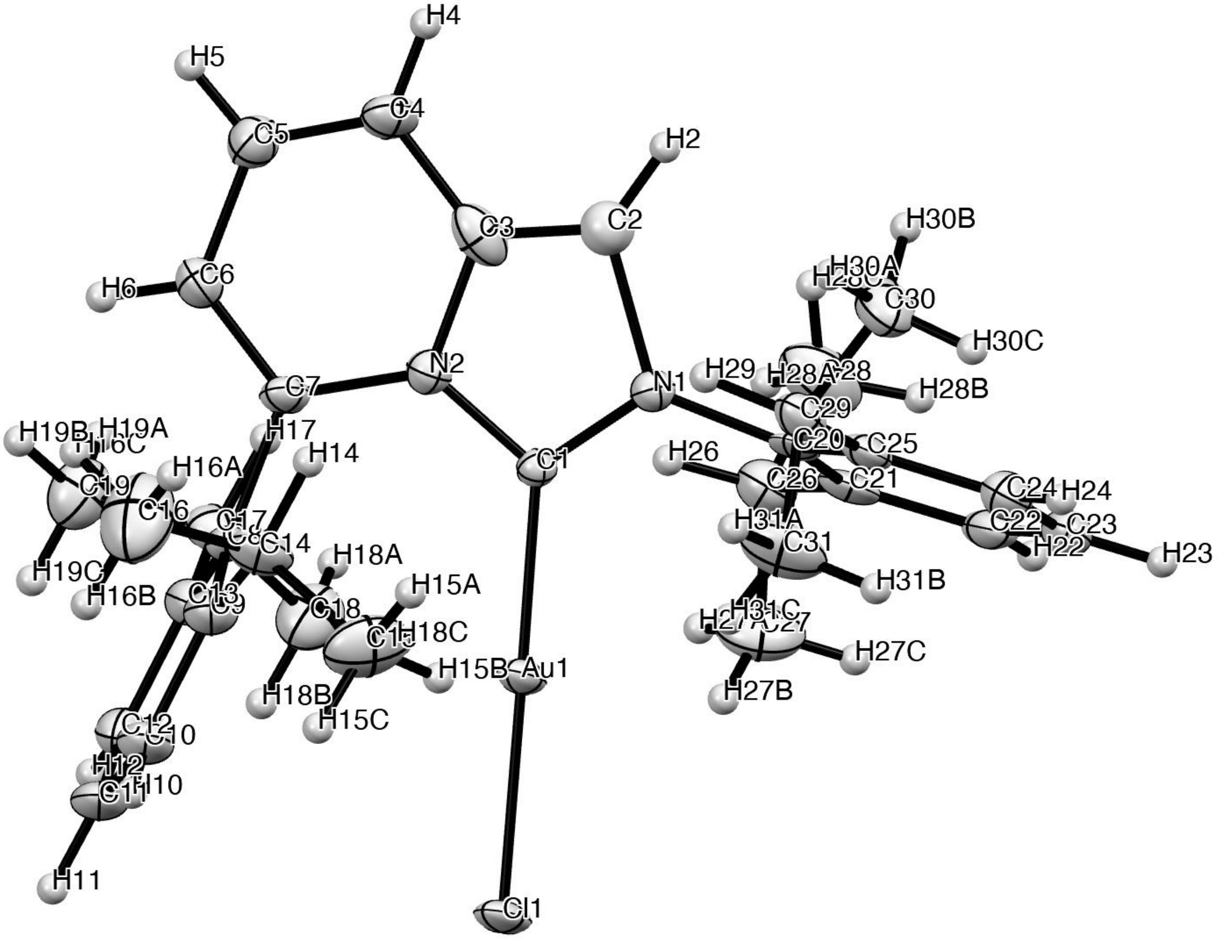

Selected bond lengths [Å] and angles [°]: Au–C1, 1.977; Au–Cl, 2.2784; C1–N1, 1.385; C1–N2, 1.354; C2–C3, 1.373; C1–Au–Cl, 178.1; Au–Cl–N2, 125.5; Au–Cl–N1, 130.2; N1–C1–N2, 104.2; C1–N1–C2, 110.5; C3–N2–C1, 112.5.

Table S1. *Crystal Data and Structure Refinement Summaries for* **7** *and* **8**.

Compound	**7**	**8**
Chemical formula	$C_{25}H_{26}AuClN_2\cdot0.492(C_4H_8O)\cdot0.508(C_4H_8O)$	$C_{32}H_{40}AuCl_3N_2$
M_r	658.99	755.97
Crystal system, space group	Triclinic, $P\bar{1}$	Triclinic, $P\bar{1}$
Temperature (K)	100	100
a, *b*, *c* (Å)	8.9996 (2), 10.9622 (2), 14.1101 (3)	9.0381 (3), 9.7983 (2), 18.9038 (7)
β (°)		
α, β, γ (°)	78.929 (1), 81.664 (2), 76.007 (1)	81.326 (2), 76.775 (3), 77.727 (2)
V ($Å^3$)	1318.48 (5)	1583.23 (9)
Z	2	2
Radiation type	Cu *K*α	Cu *K*α
μ (mm^{-1})	11.59	11
Crystal size (mm)	0.51 × 0.10 × 0.06	0.3 × 0.2 × 0.1
Diffractometer	Bruker *SMART* CCD Apex-II area-detector	XtaLAB Synergy R, DW system, HyPix-Arc 150
Absorption correction	Numerical *SADABS* 2016/2: Krause, L., Herbst-Irmer, R., Sheldrick G.M. & Stalke D., J. Appl. Cryst. 48 (2015) 3-10	Multi-scan SCALE3 ABSPACK (Rigaku Oxford Diffraction, 2015).
T_{min}, T_{max}	0.333, 0.753	0.536 , 1.000
No. of measured, independent and observed [$I > 2\sigma(I)$] reflections	16929, 4472, 4126	18078, 5444, 5338
R_{int}	0.042	0.029
$(\sin \theta/\lambda)_{max}$ ($Å^{-1}$)	0.596	0.617
$R[F^2 > 2\sigma(F^2)]$, $wR(F^2)$, S	0.039, 0.107, 1.04	0.065, 0.164, 1.10
No. of reflections	4473	5444
No. of parameters	347	348
H-atom treatment	H-atom parameters constrained	H-atom parameters constrained
$\Delta\rho_{max}$, $\Delta\rho_{min}$ (e $Å^{-3}$)	3.99, −0.91	7.52, −2.19

Computer programs: *APEX* 2 (Bruker, 2006), *APEX* 2, *SAINT* (Bruker, 2005), *SHELXL2016*/6 (Sheldrick, 2016), *SHELXTL*.

Computational Methods

Computational Methods. All the calculations were performed using Gaussian 09 suite of programs. All of the geometry optimizations were performed at the B3LYP level of theory in the gas phase with the QZVP basis set for gold and the 6-311++G(d,p) basis set for the other atoms. For geometry optimizations, we employed the X-ray structures of [(ImPyDippDipp)AuCl] and [(ImPyMesMes)AuCl] as the starting geometry and performed full optimization. The absence of imaginary frequencies was used to characterize the structures as minima on the potential energy surface. All of the optimized geometries were verified as minima (no imaginary frequencies). NBO calculations were performed at the DFT/B3LYP level using NBO program implemented in Gaussian software package. Wiberg bond indices were calculated by the NBO method (*J. Mol. Struc. Theochem* **2008**, *870*, 1). Energetic parameters were calculated under standard conditions (298.15 K and 1 atm). Structural representations were generated using CYLview software (Legault, C. Y. CYL view version 1.0 BETA, University of Sherbrooke). All other representations were generated using Gauss View (GaussView, version 5, Dennington, R.; Keith, T.; Millam, J. Semichem Inc., Shawnee Mission, KS, 2009) or ChemCraft software (Andrienko, G. L. ChemCraft version b562a, https://www.chemcraftprog.com).

Full Reference for Gaussian 09

Gaussian 09, Revision D.01, Frisch, M. J.; Trucks, G. W.; Schlegel, H. B.; Scuseria, G. E.; Robb, M. A.; Cheeseman, J. R.; Scalmani, G.; Barone, V.; Mennucci, B.; Petersson, G. A.; Nakatsuji, H.; Caricato, M.; Li, X.; Hratchian, H. P.; Izmaylov, A. F.; Bloino, J.; Zheng, G.; Sonnenberg, J. L.; Hada, M.; Ehara, M.; Toyota, K.; Fukuda, R.; Hasegawa, J.; Ishida, M.; Nakajima, T.; Honda, Y.; Kitao, O.; Nakai, H.; Vreven, T.; Montgomery, J. A., Jr.; Peralta, J. E.; Ogliaro, F.; Bearpark, M.; Heyd, J. J.; Brothers, E.; Kudin, K. N.; Staroverov, V. N.; Kobayashi, R.; Normand, J.; Raghavachari, K.; Rendell, A.; Burant, J. C.; Iyengar, S. S.; Tomasi, J.; Cossi, M.; Rega, N.; Millam, M. J.; Klene, M.; Knox, J. E.; Cross, J. B.; Bakken, V.; Adamo, C.; Jaramillo, J.; Gomperts, R.; Stratmann, R. E.; Yazyev, O.; Austin, A. J.; Cammi, R.; Pomelli, C.; Ochterski, J. W.; Martin, R. L.; Morokuma, K.; Zakrzewski, V. G.; Voth, G. A.; Salvador, P.; Dannenberg, J. J.; Dapprich, S.; Daniels, A. D.; Farkas, Ö.; Foresman, J. B.; Ortiz, J. V.; Cioslowski, J.; Fox, D. J. Gaussian, Inc., Wallingford CT, 2009.

^{1}H and ^{13}C NMR Spectra

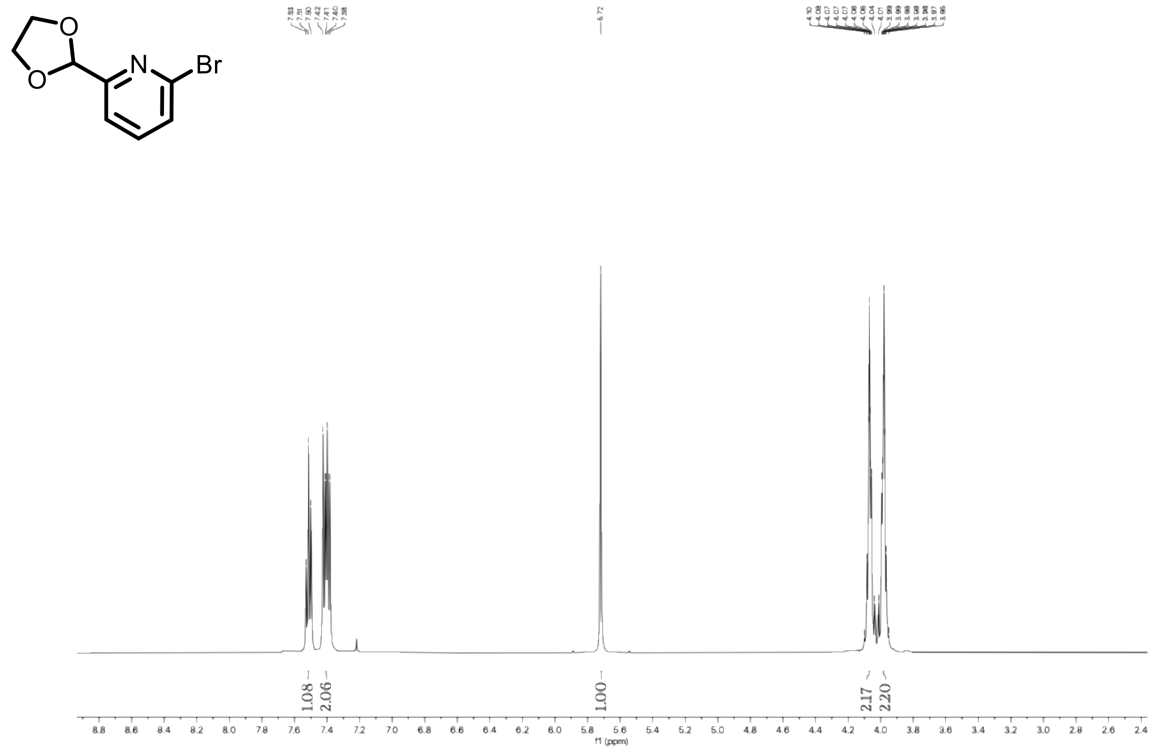

Figure S3. ^{1}H NMR (500 MHz, $CDCl_3$) Spectrum of **2**

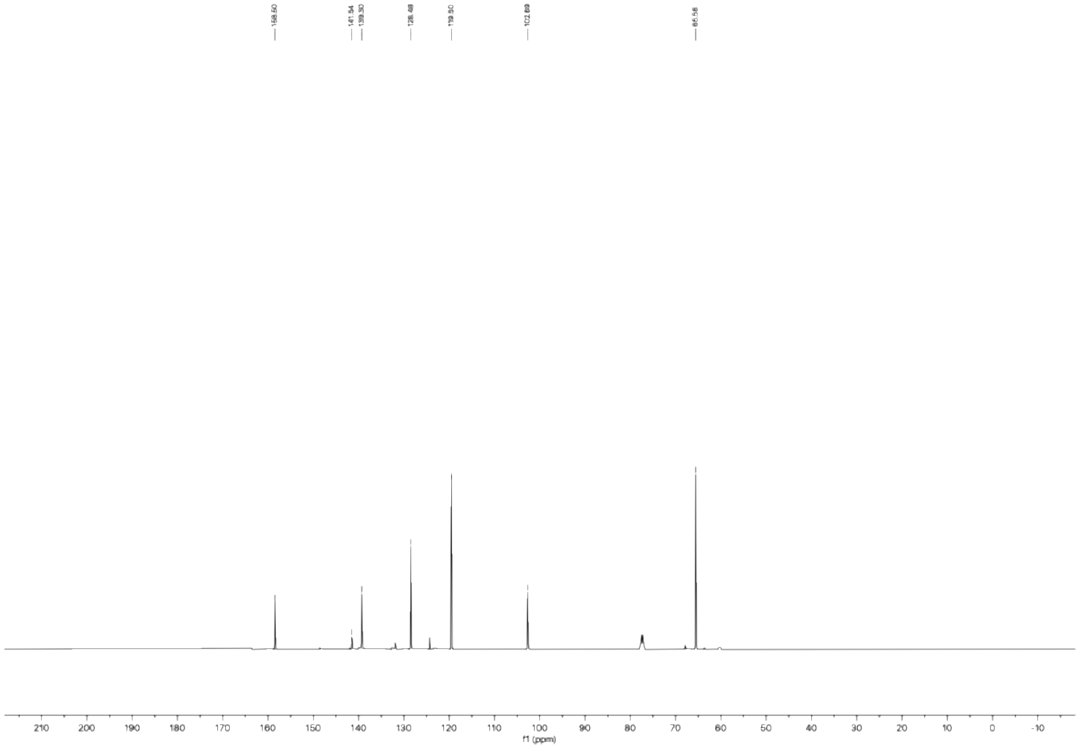

Figure S4. ^{13}C{^{1}H} NMR (125 MHz, $CDCl_3$) Spectrum of **2**

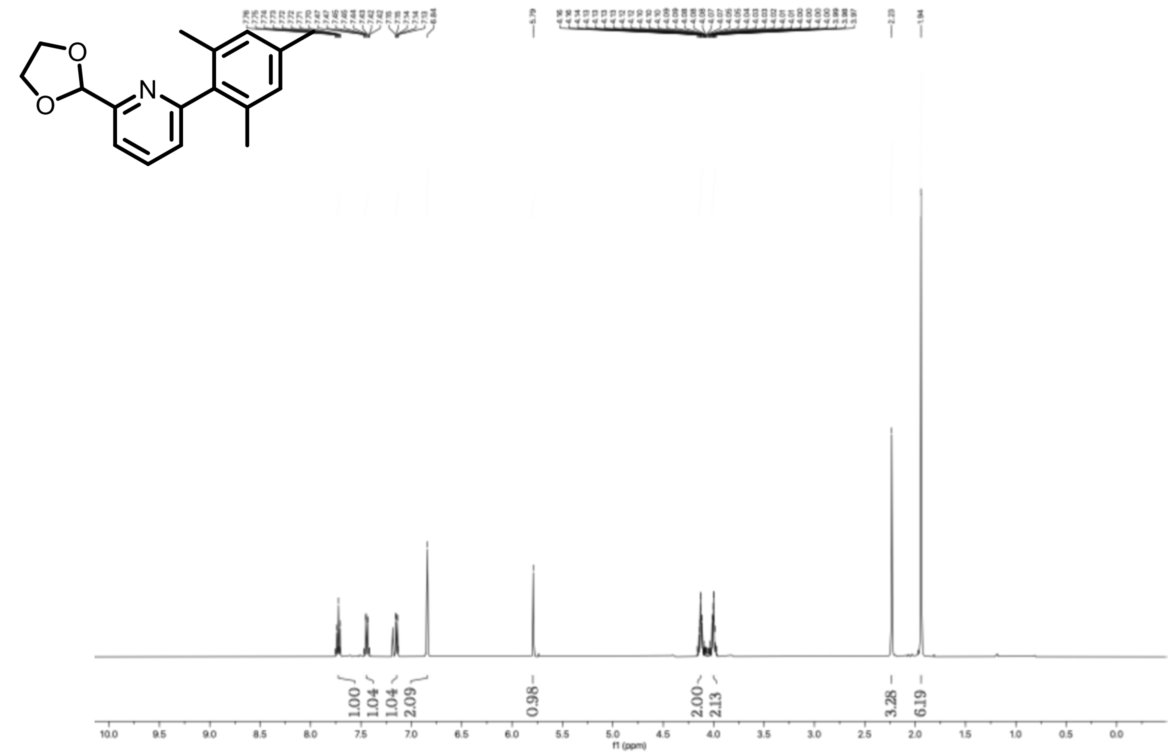

Figure S5. ^{1}H NMR (500 MHz, $CDCl_3$) Spectrum of **3**

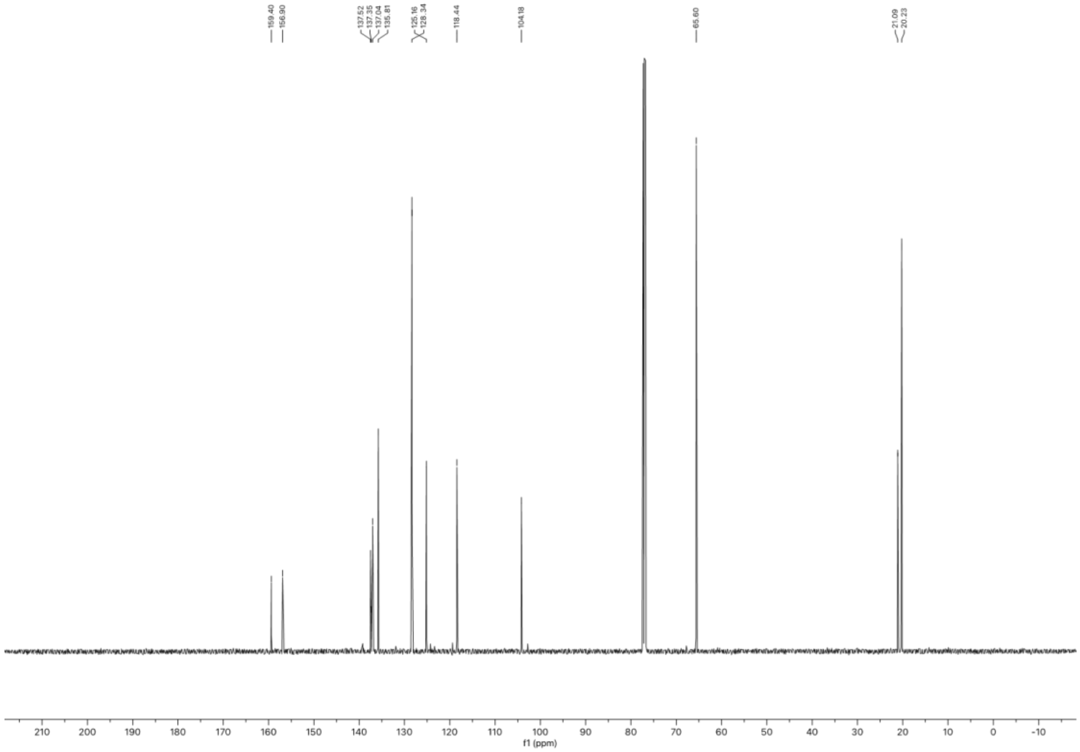

Figure S6. $^{13}C\{^{1}H\}$ NMR (125 MHz, $CDCl_3$) Spectrum of **3**

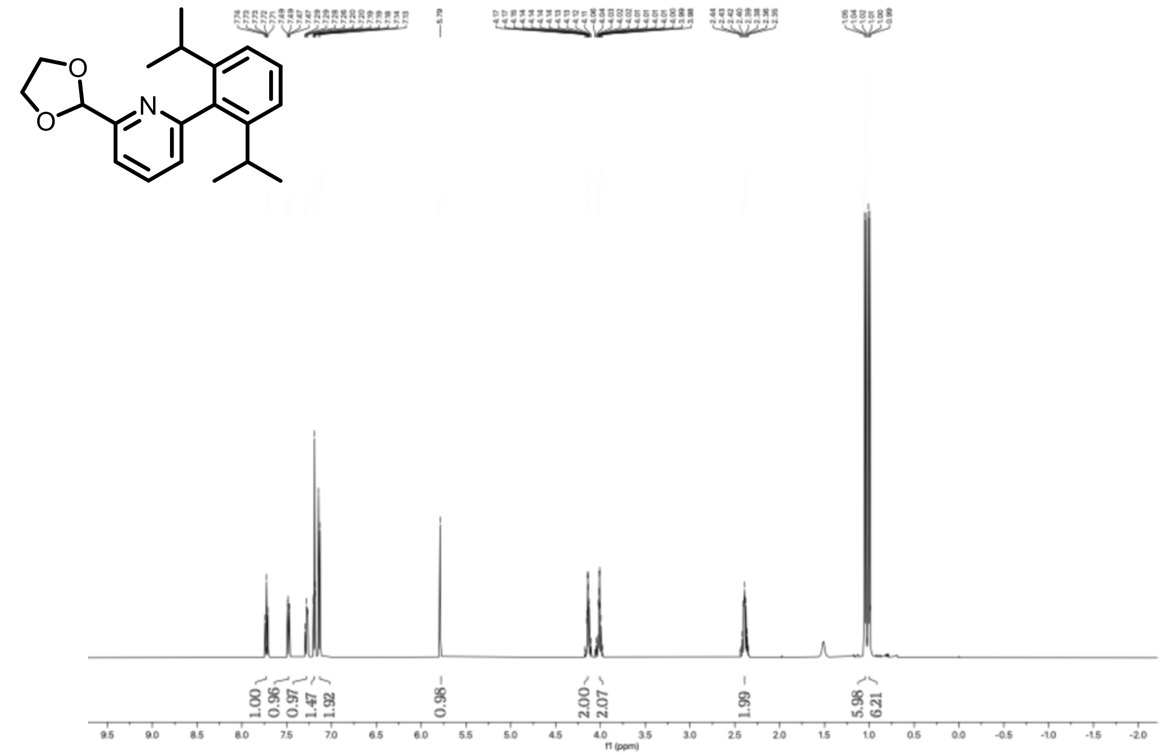

Figure S7. ^{1}H NMR (500 MHz, $CDCl_3$) Spectrum of **4**

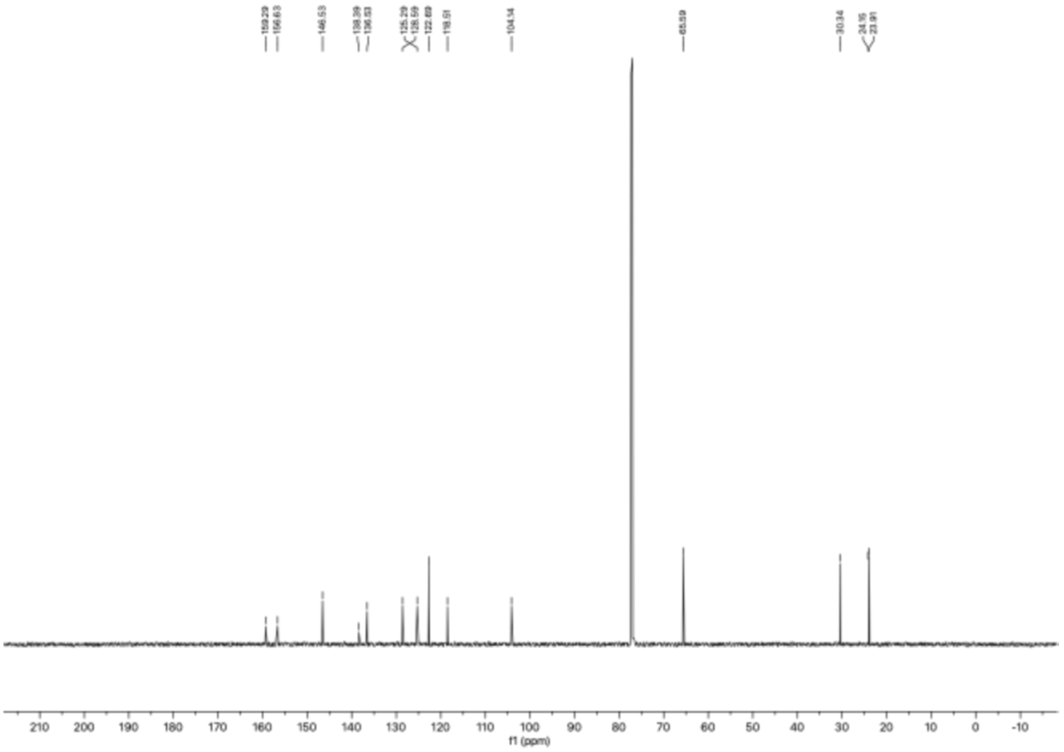

Figure S8. $^{13}C\{^{1}H\}$ NMR (125 MHz, $CDCl_3$) Spectrum of **4**

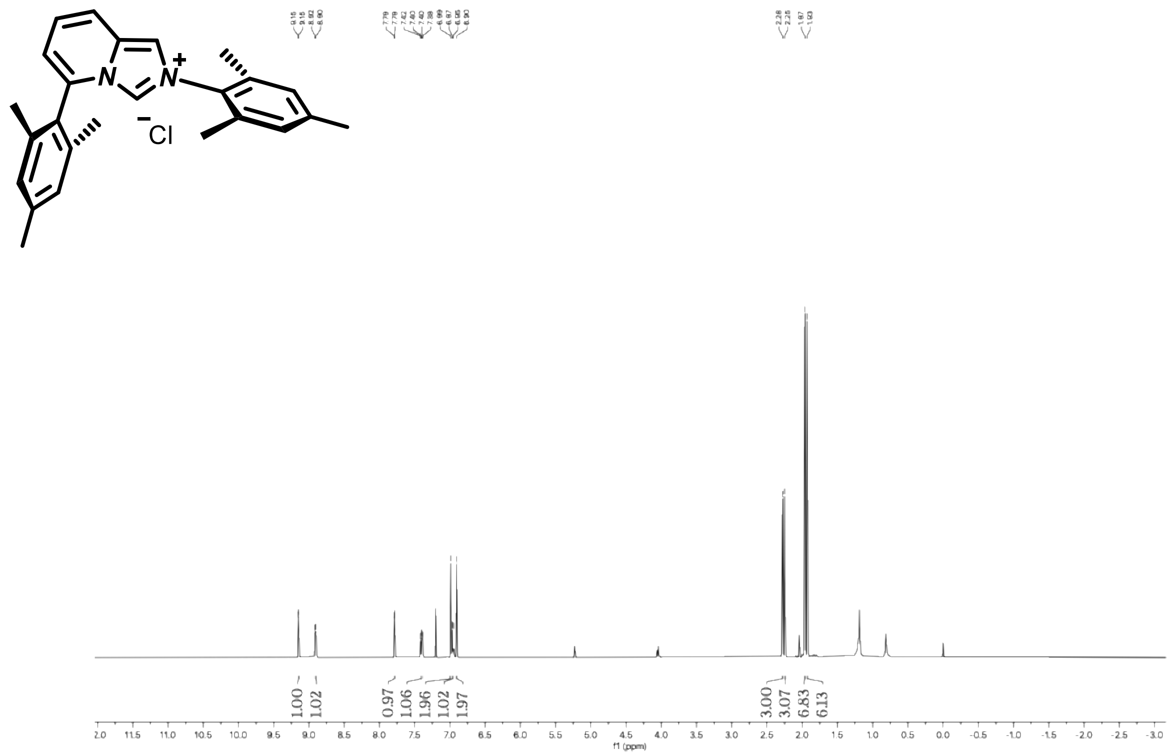

Figure S9. ^{1}H NMR (500 MHz, $CDCl_3$) Spectrum of **5**

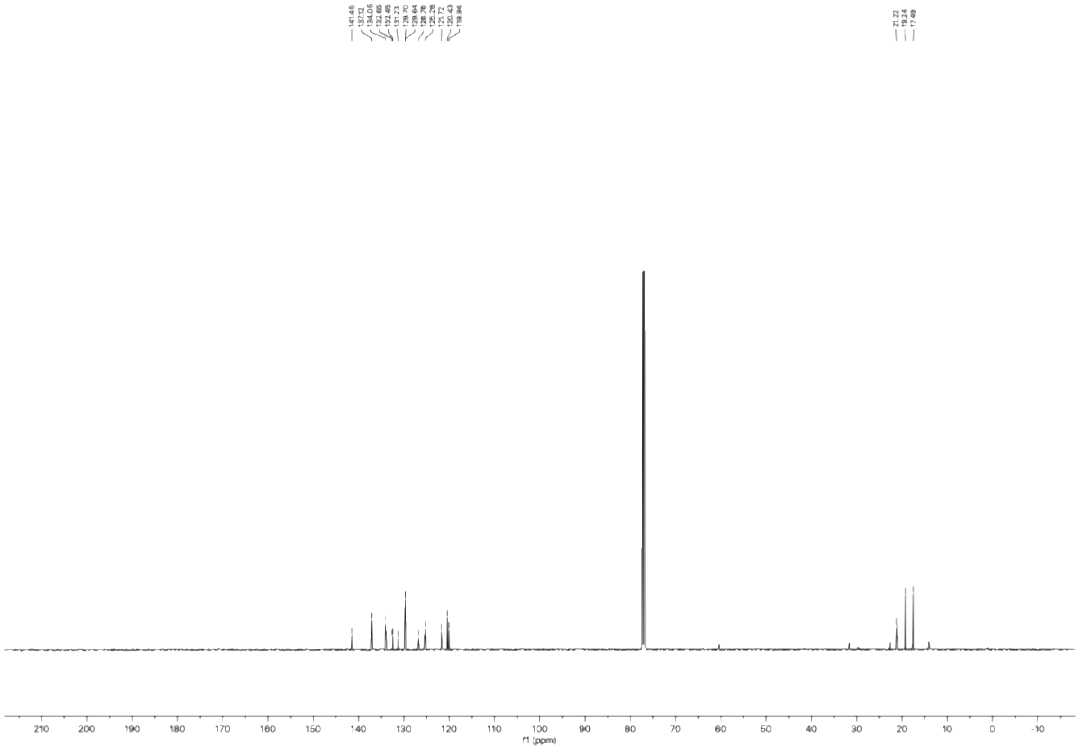

Figure S10. $^{13}C\{^{1}H\}$ NMR (125 MHz, $CDCl_3$) Spectrum of **5**

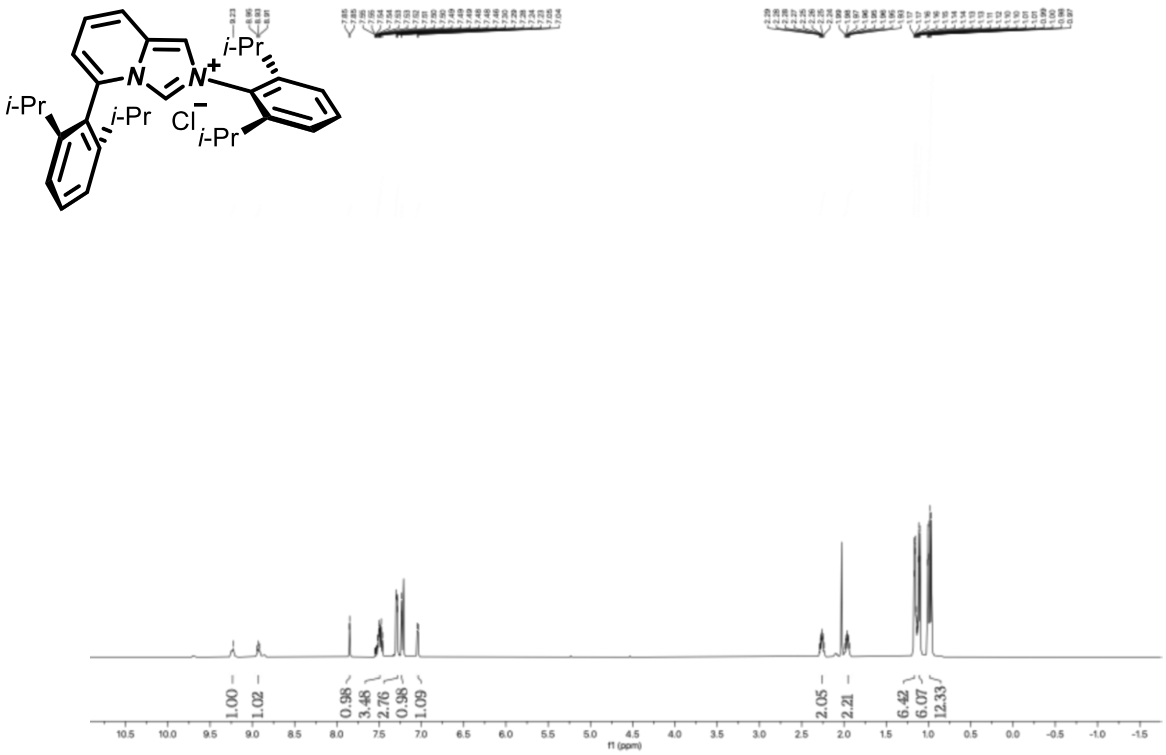

Figure S11. ^{1}H NMR (500 MHz, $CDCl_3$) Spectrum of **6**

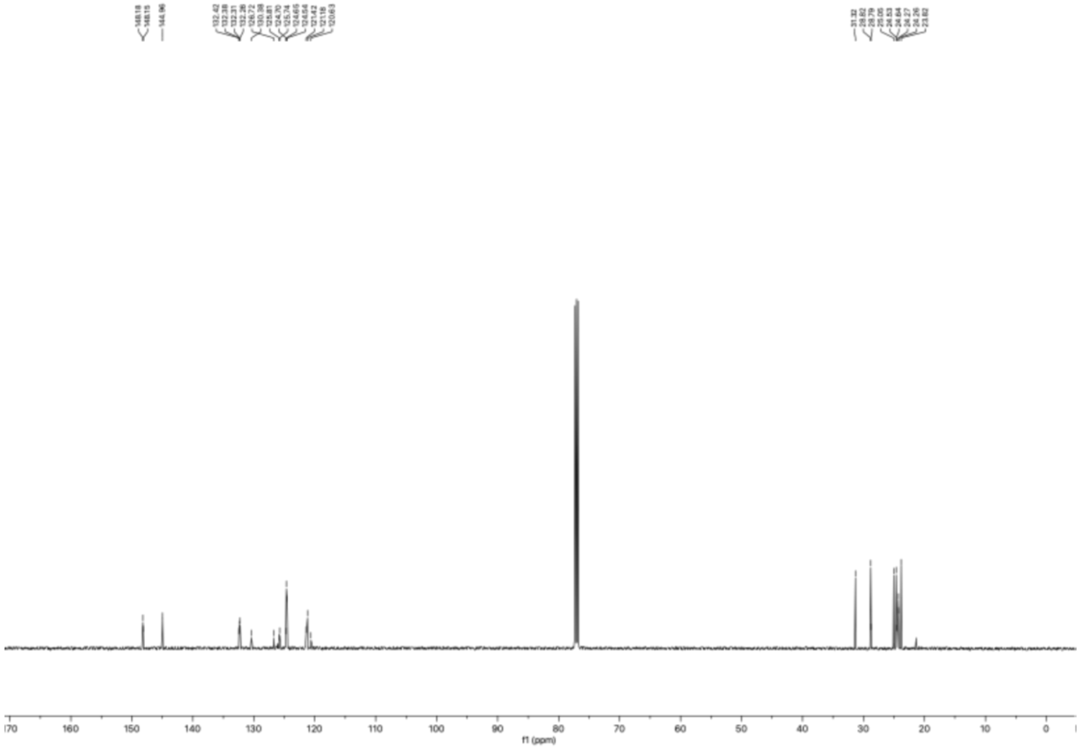

Figure S12. $^{13}C\{^{1}H\}$ NMR (125 MHz, $CDCl_3$) Spectrum of **6**

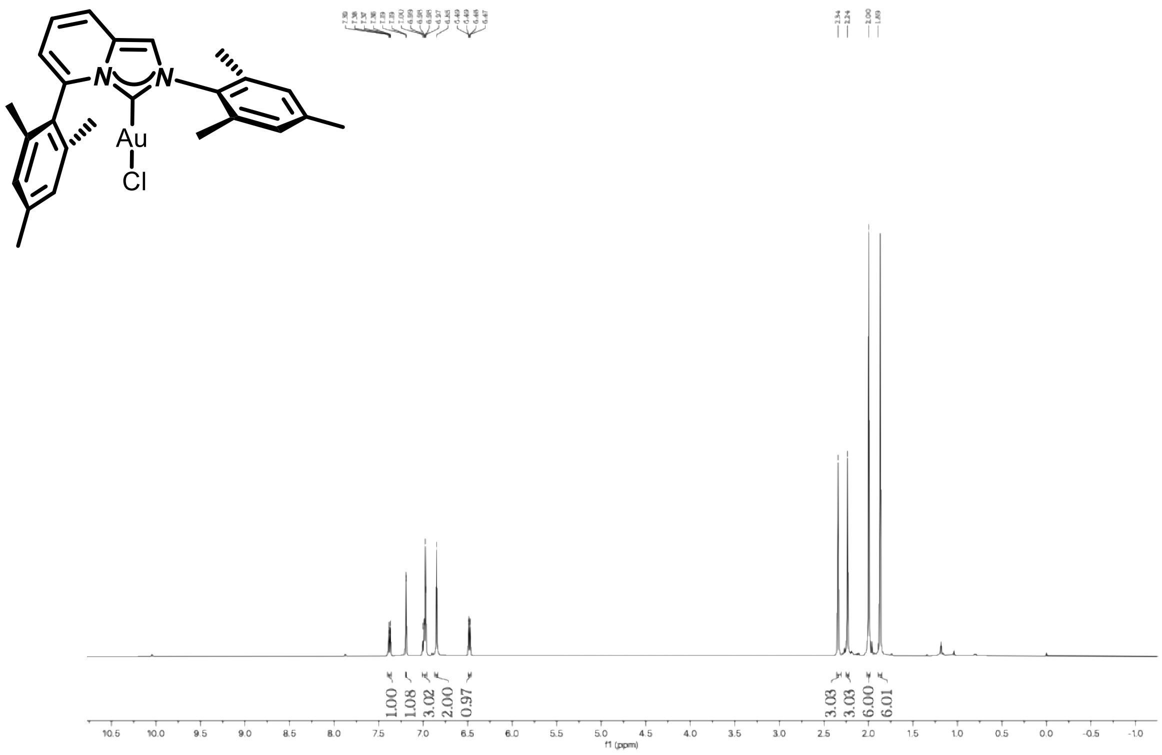

Figure S13. ^{1}H NMR (500 MHz, $CDCl_3$) Spectrum of **7**

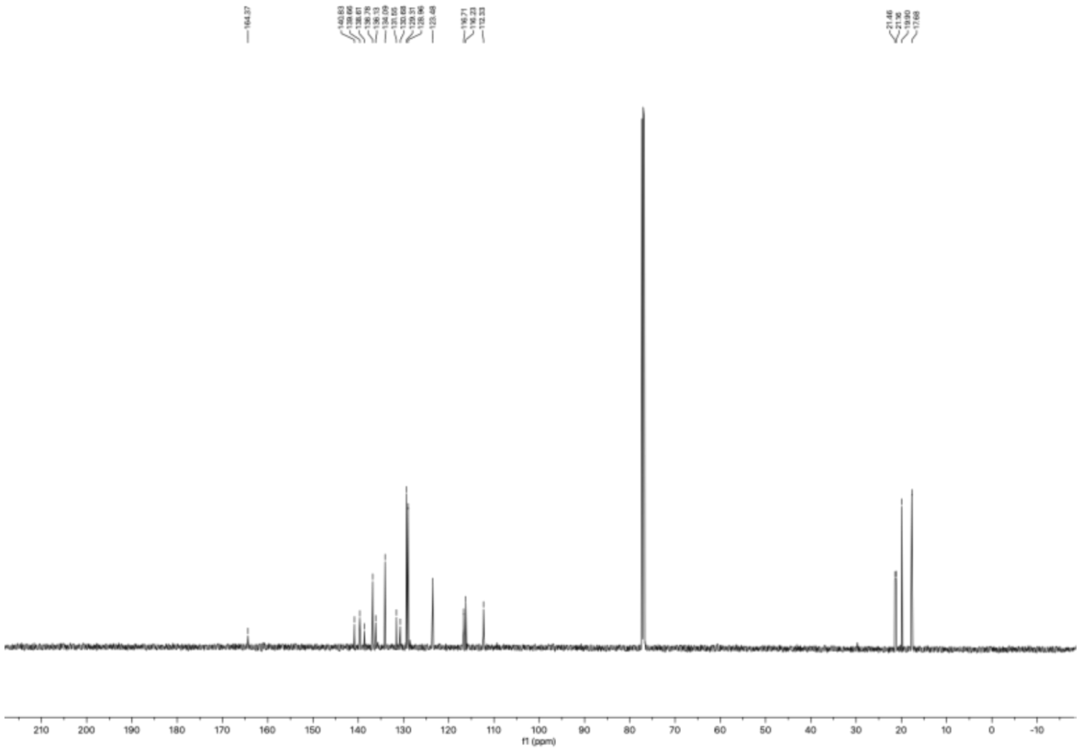

Figure S14. $^{13}C\{^{1}H\}$ NMR (125 MHz, $CDCl_3$) Spectrum of **7**

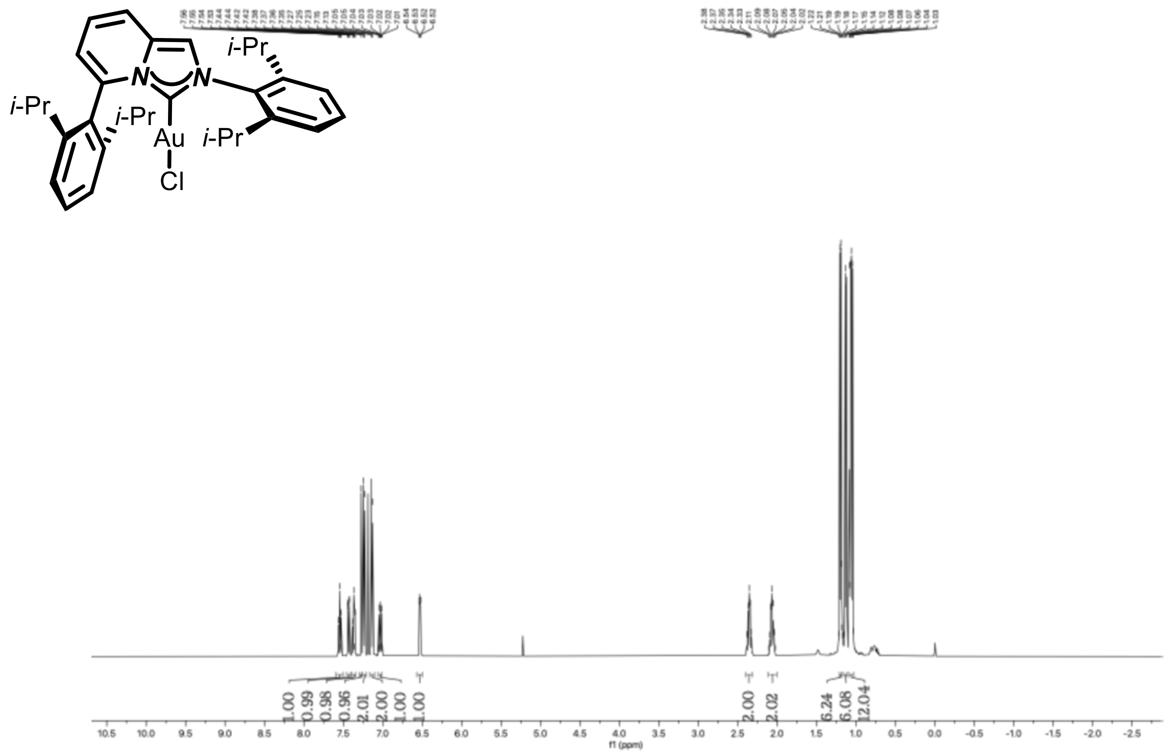

Figure S15. ^{1}H NMR (500 MHz, $CDCl_3$) Spectrum of **8**

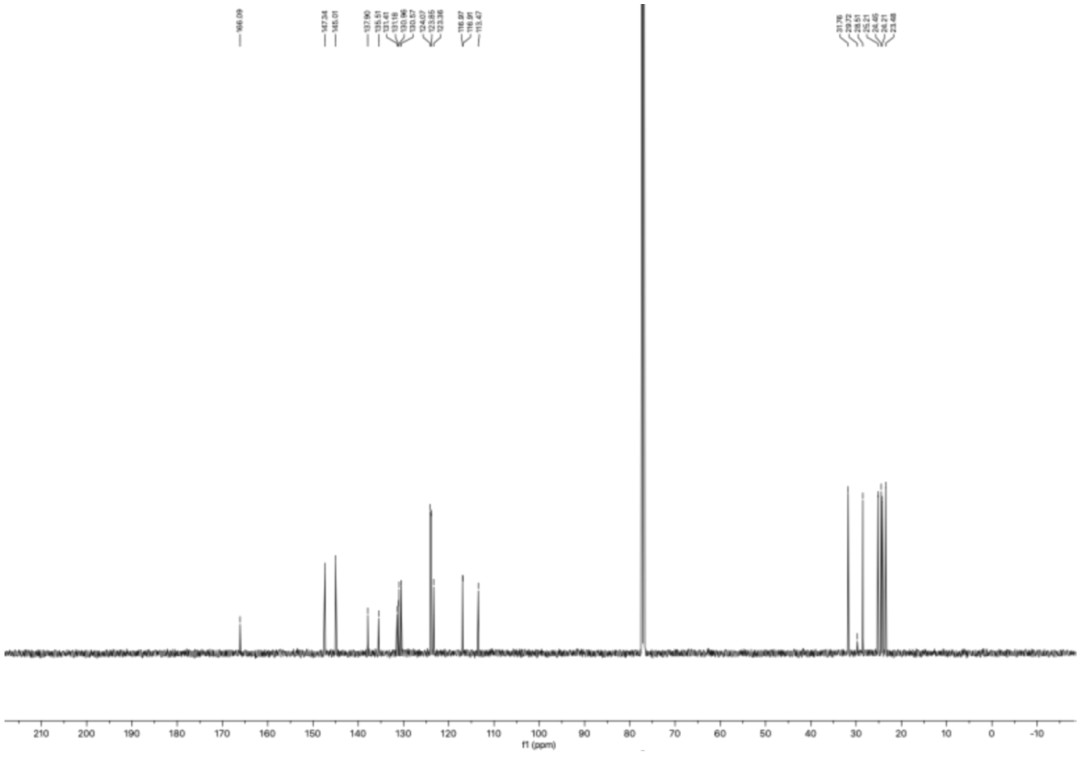

Figure S16. $^{13}C\{^{1}H\}$ NMR (125 MHz, $CDCl_3$) Spectrum of **8**

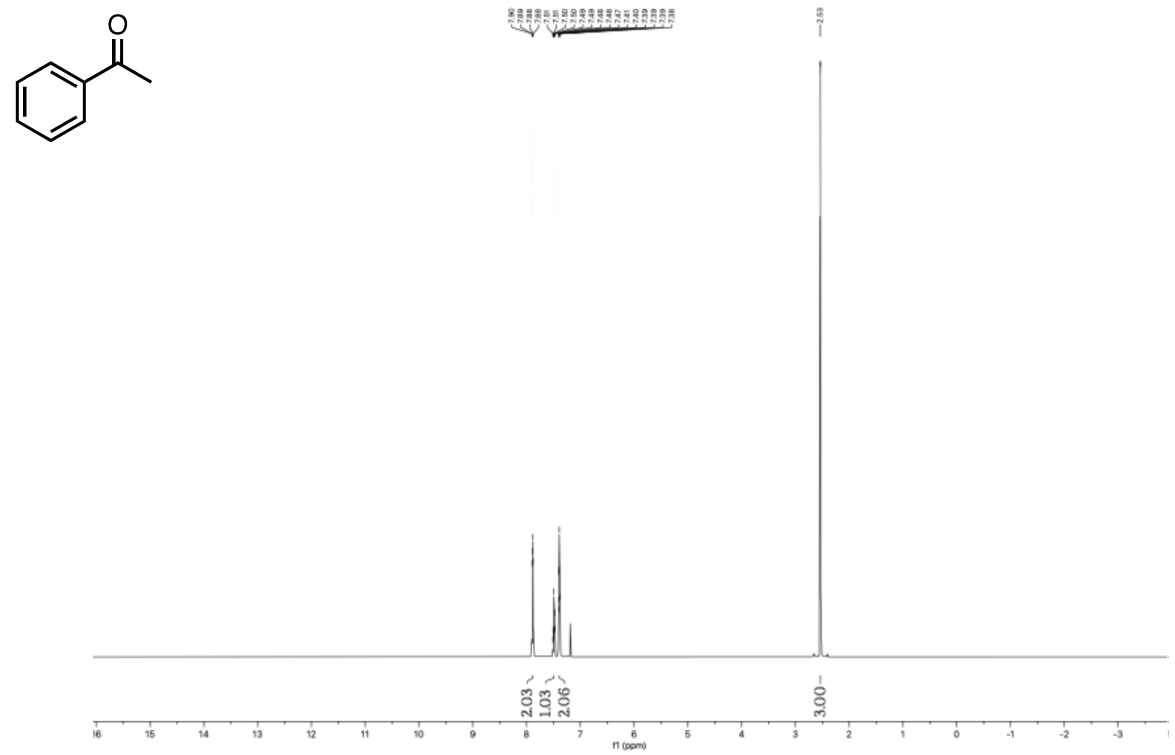

Figure S17. ^{1}H NMR (500 MHz, $CDCl_3$) Spectrum of **10a**

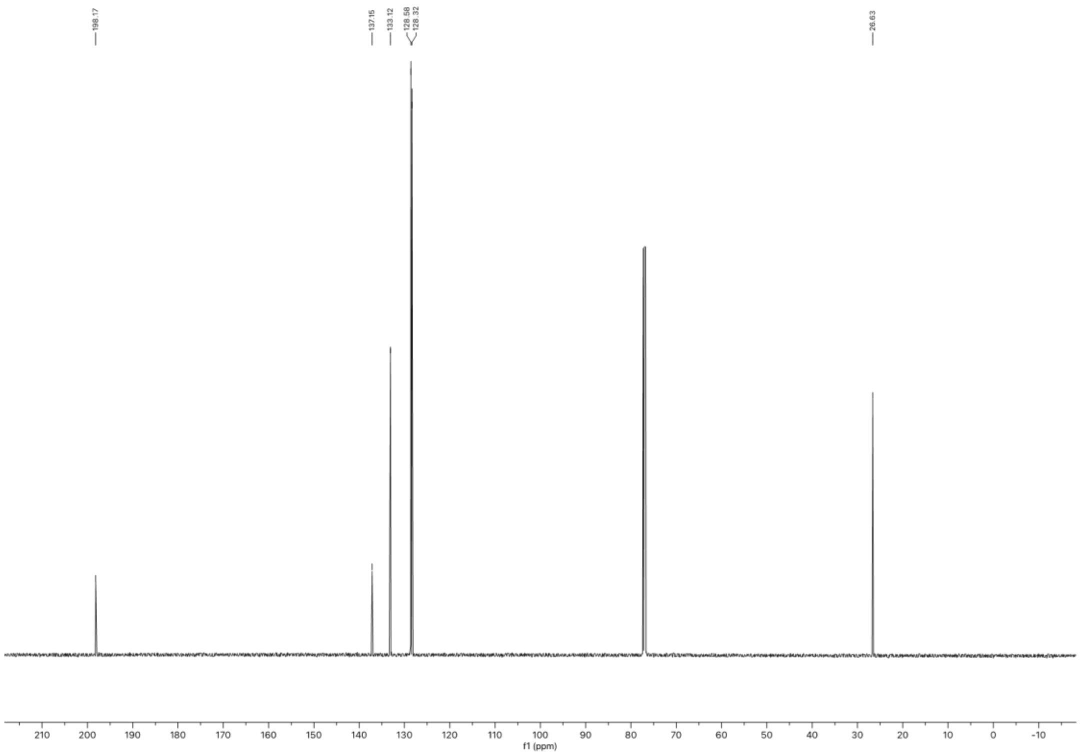

Figure S18. $^{13}C\{^{1}H\}$ NMR (125 MHz, $CDCl_3$) Spectrum of **10a**

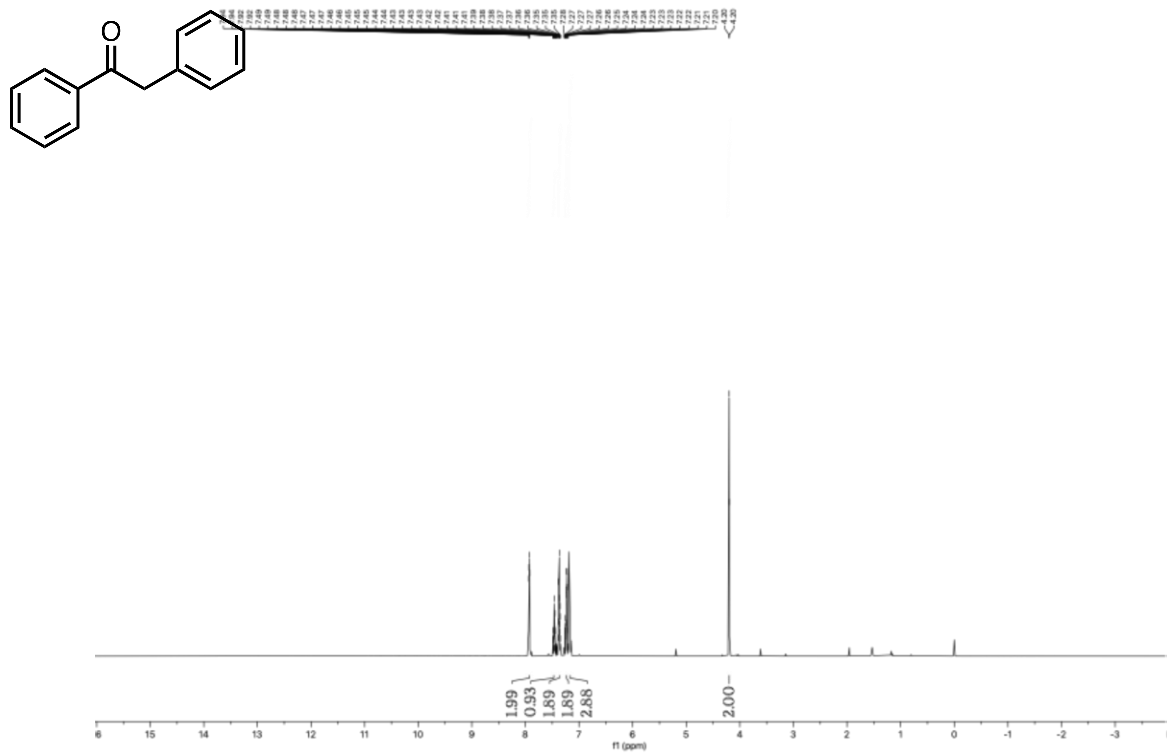

Figure S19. ^{1}H NMR (500 MHz, $CDCl_3$) Spectrum of **10b**

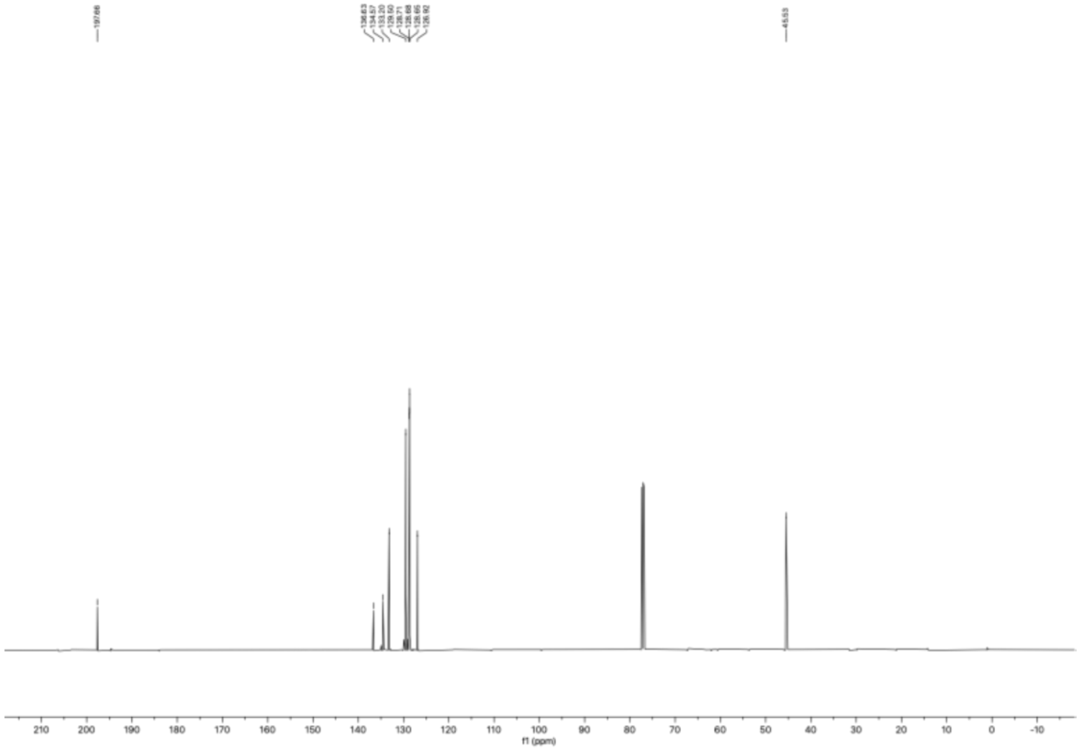

Figure S20. ^{13}Cf{^{1} H} NMR (125 MHz, $CDCl_3$) Spectrum of **10b**

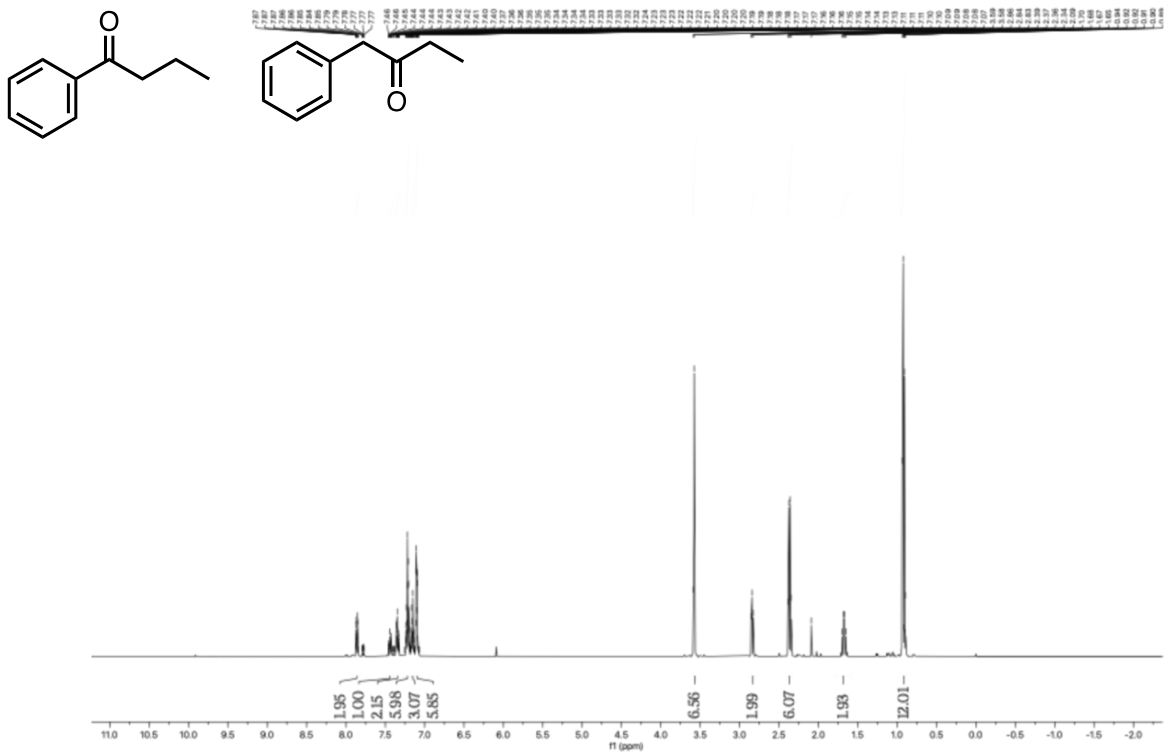

Figure S21. ^{1}H NMR (500 MHz, $CDCl_3$) Spectrum of **10c**

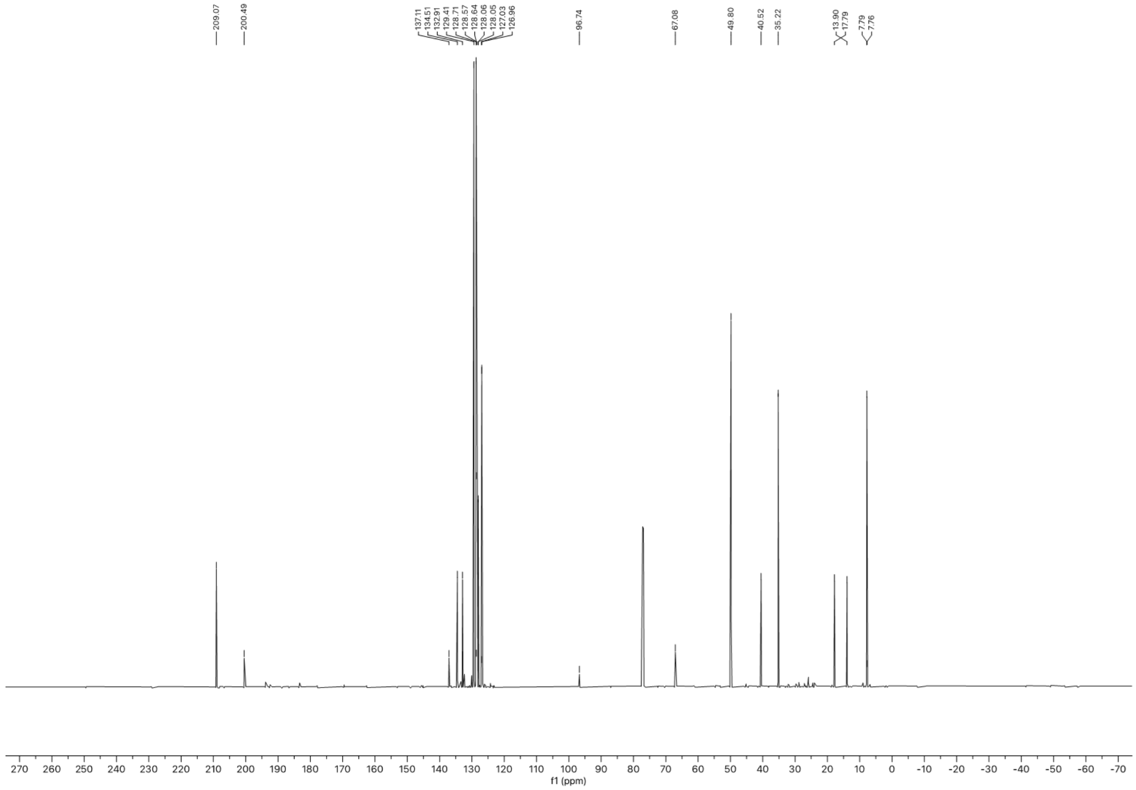

Figure S22. $^{13}C\{^{1}H\}$ NMR (125 MHz, $CDCl_3$) Spectrum of **10c**

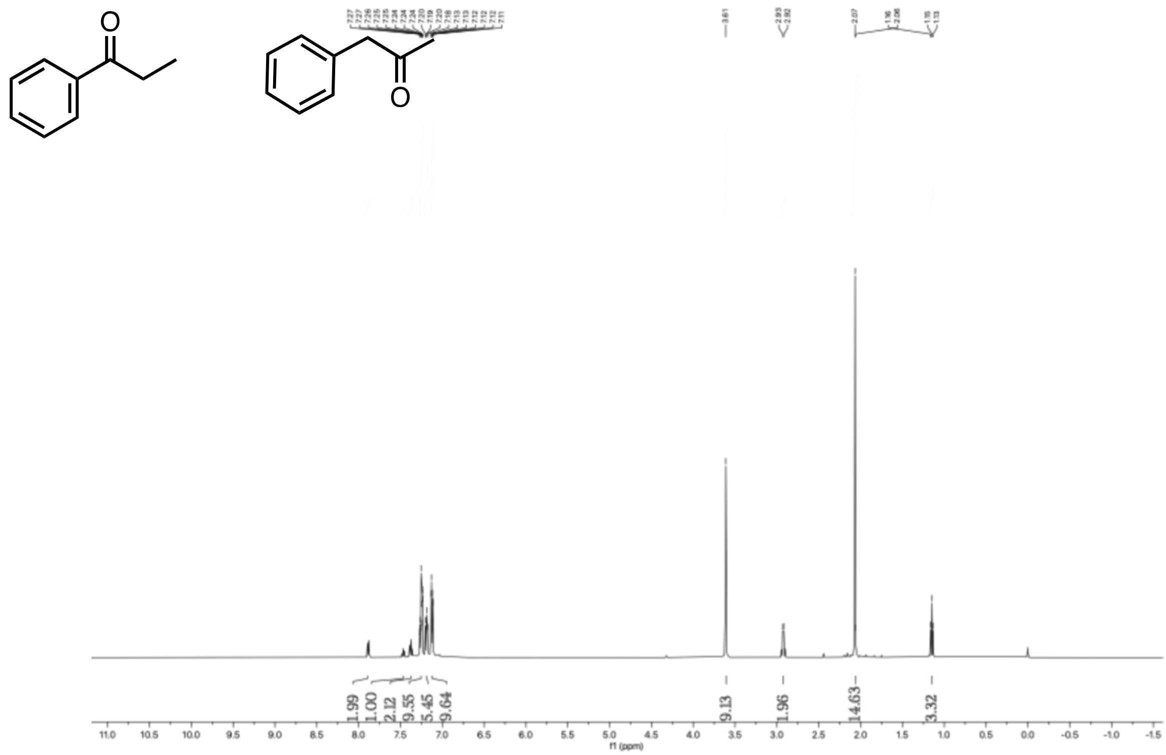

Figure S23. ^{1}H NMR (500 MHz, $CDCl_3$) Spectrum of **10d**

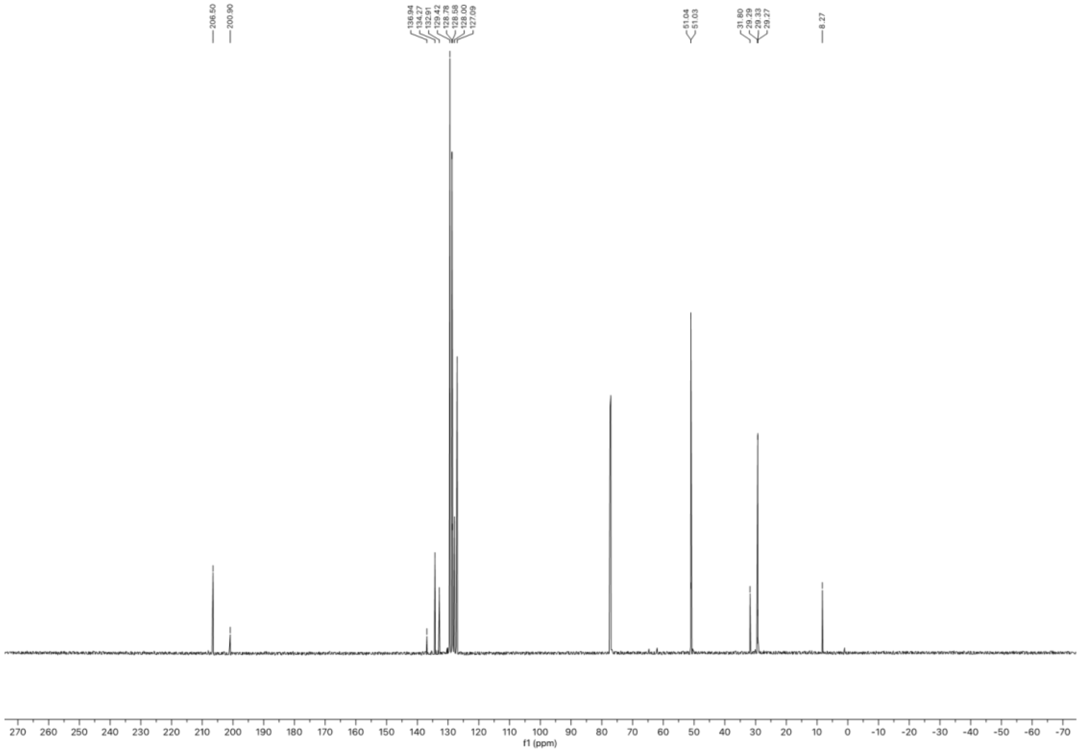

Figure S24. $^{13}C\{^{1}H\}$ NMR (125 MHz, $CDCl_3$) Spectrum of **10d**

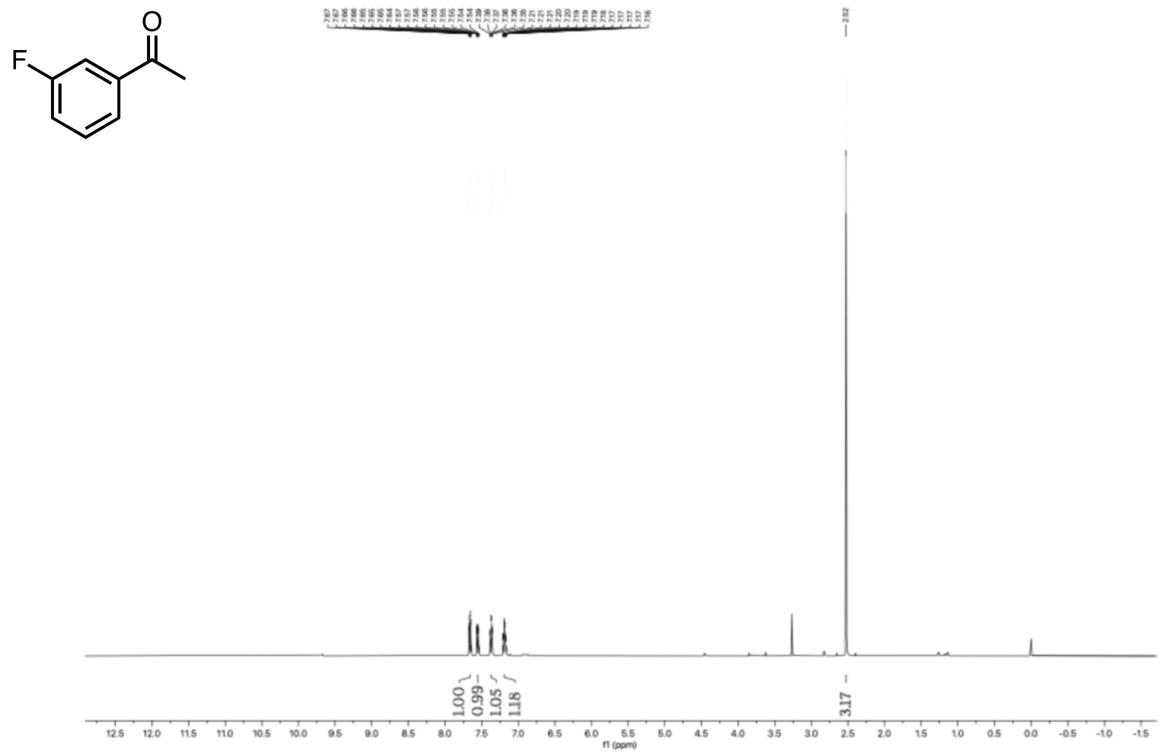

Figure S25. ^{1}H NMR (500 MHz, $CDCl_3$) Spectrum of **10e**

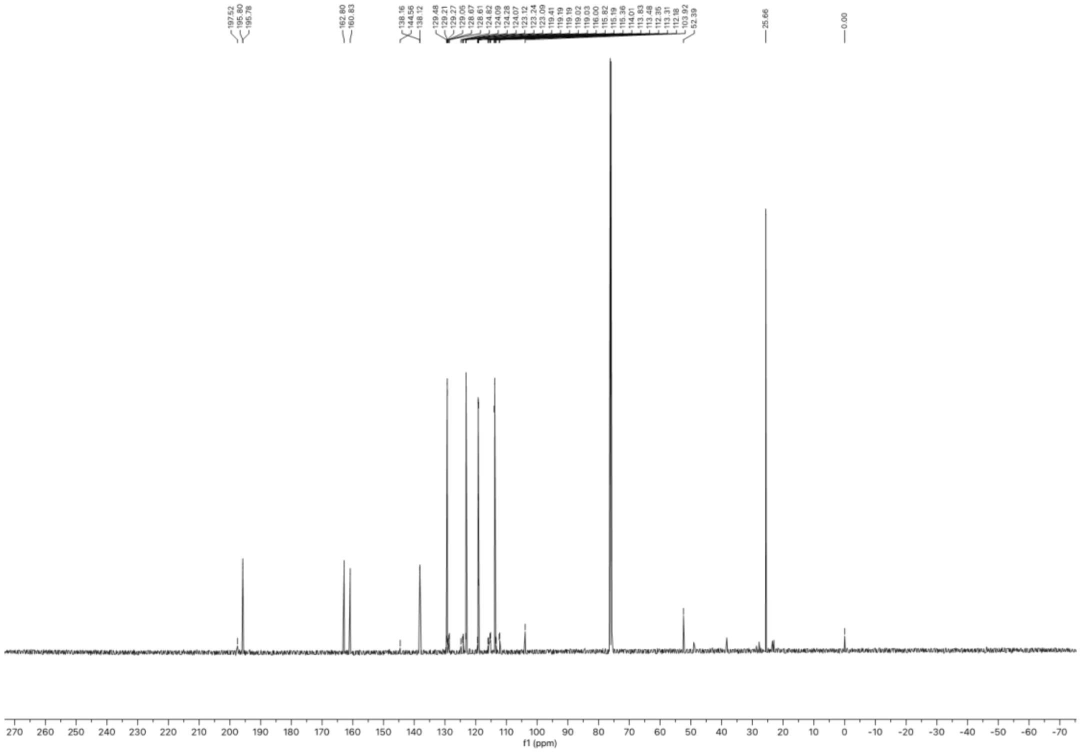

Figure S26. $^{13}C\{^{1}H\}$ NMR (125 MHz, $CDCl_3$) Spectrum of **10e**

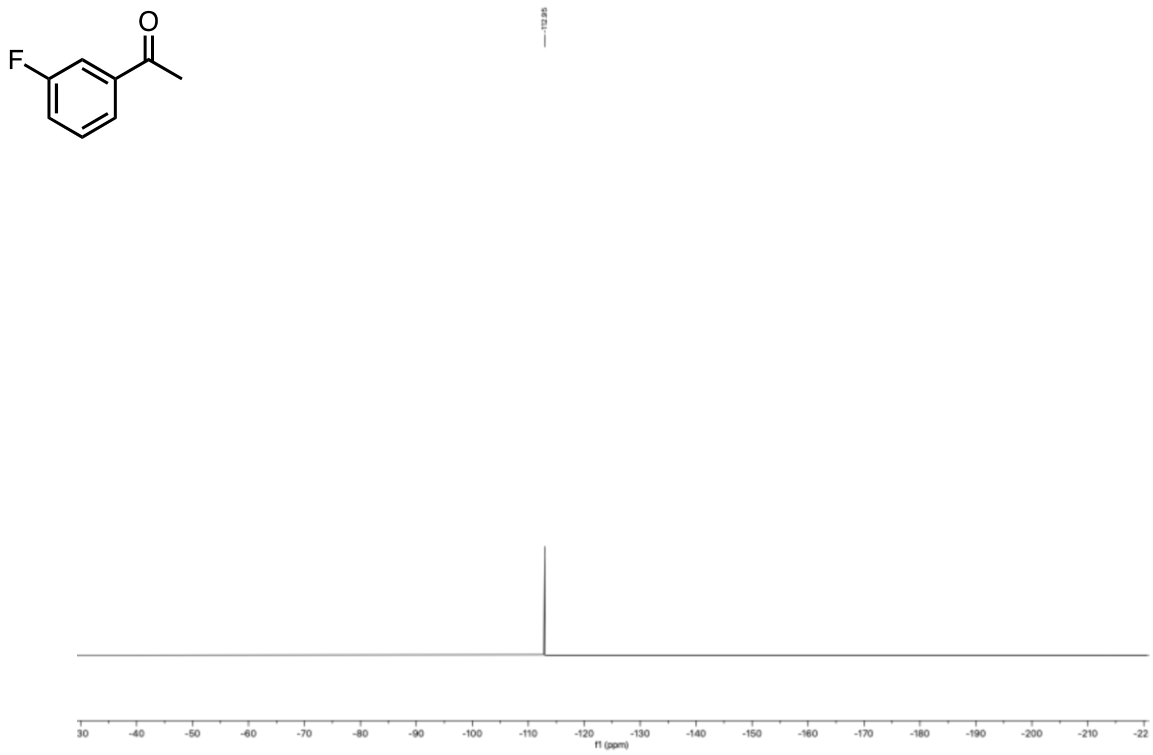

Figure S27. $^{19}F\{^{1}H\}$ NMR (471 MHz, $CDCl_3$) Spectrum of **10e**

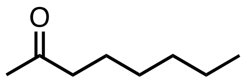

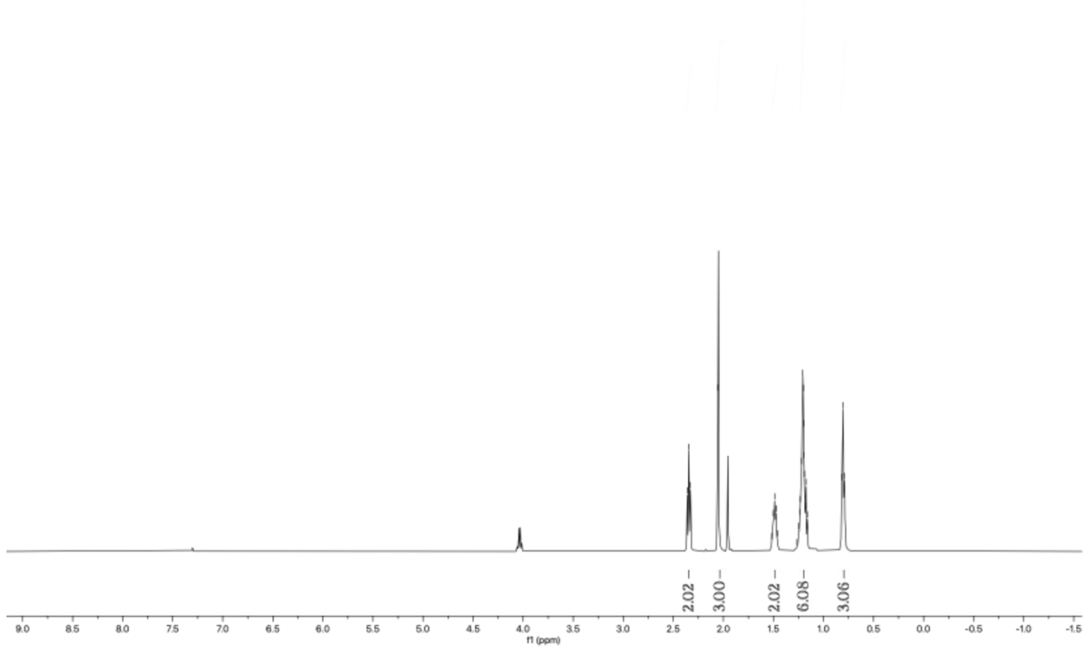

Figure S28. ^{1}H NMR (500 MHz, $CDCl_3$) Spectrum of **10f**

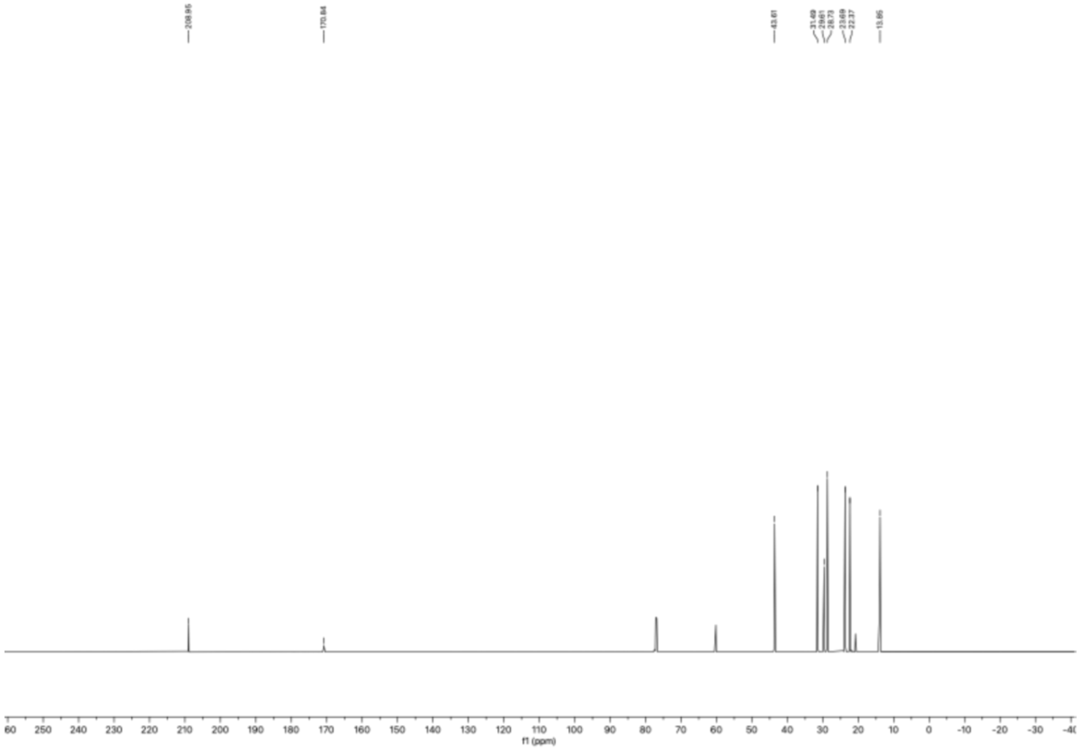

Figure S29. $^{13}C\{^{1}H\}$ NMR (125 MHz, $CDCl_3$) Spectrum of **10f**

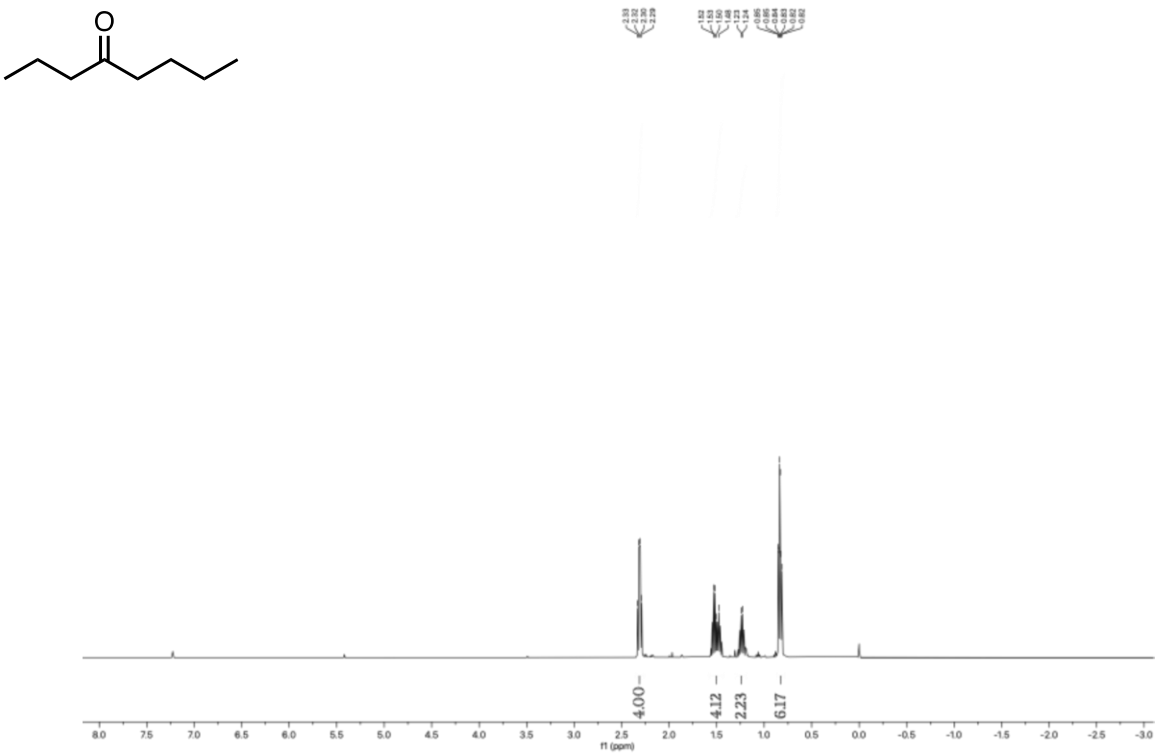

Figure S30. ^{1}H NMR (500 MHz, $CDCl_3$) Spectrum of **10g**

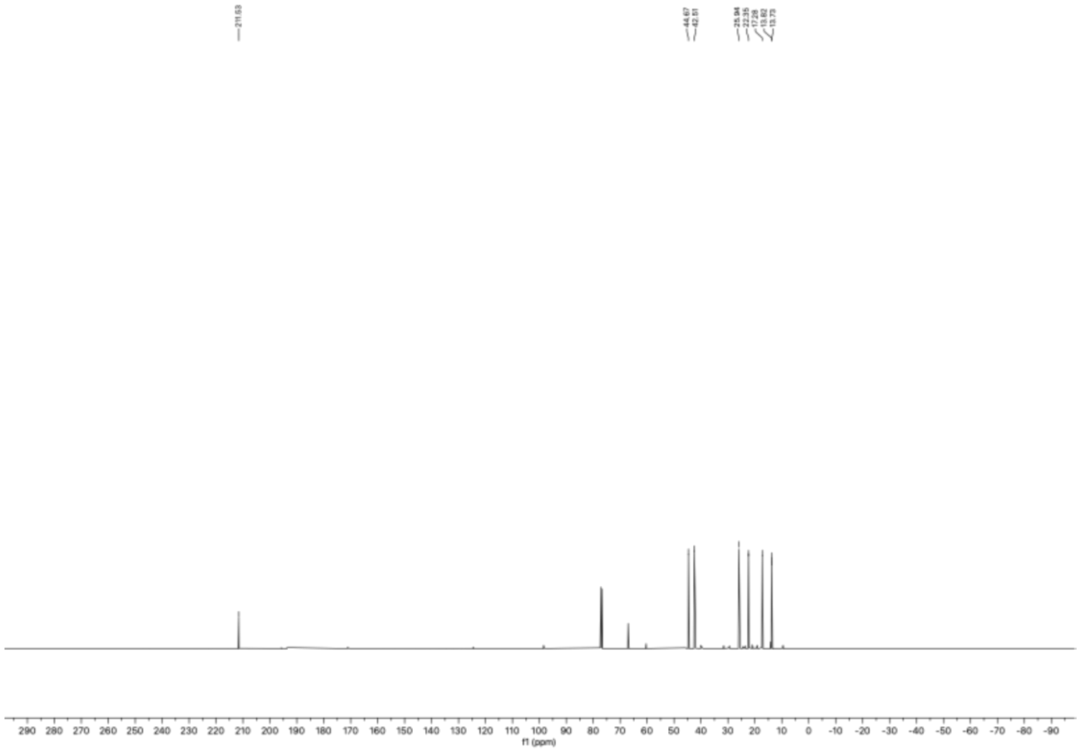

Figure S31. $^{13}C\{^{1}H\}$ NMR (125 MHz, $CDCl_3$) Spectrum of **10g**

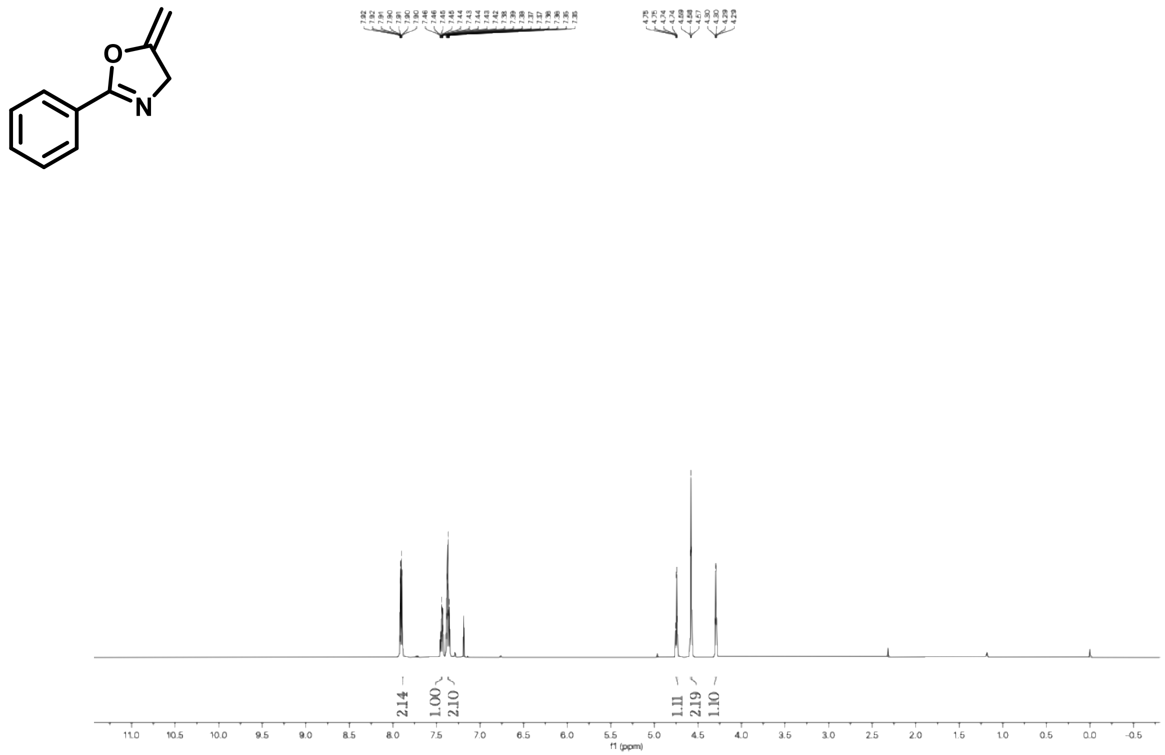

Figure S32. ^{1}H NMR (500 MHz, $CDCl_3$) Spectrum of **12a**

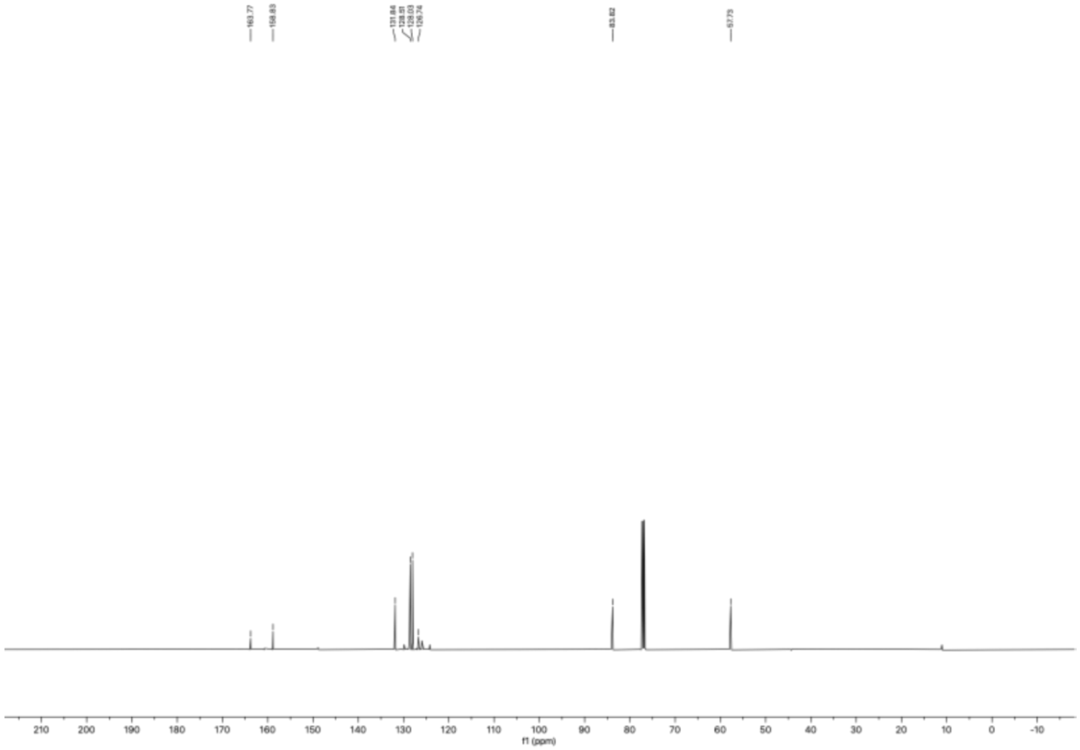

Figure S33. $^{13}C\{^{1}H\}$ NMR (125 MHz, $CDCl_3$) Spectrum of **12a**

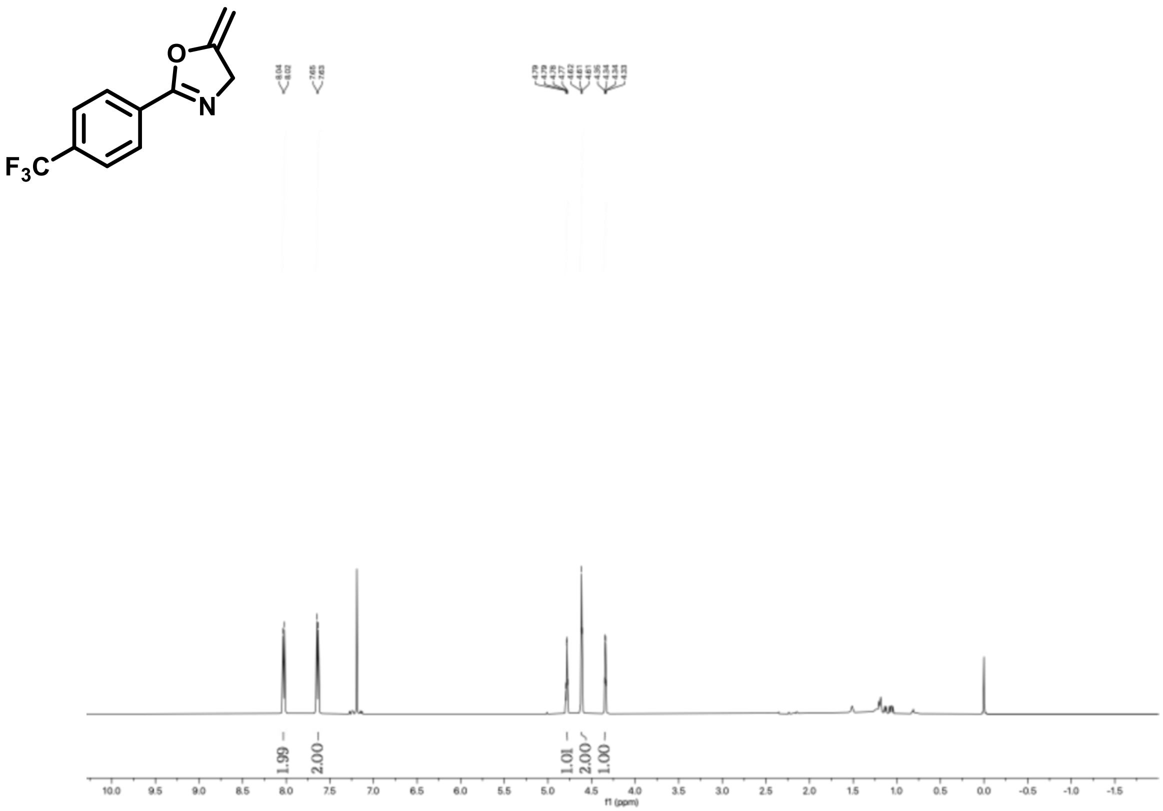

Figure S34. ^{1}H NMR (500 MHz, $CDCl_3$) Spectrum of **12b**

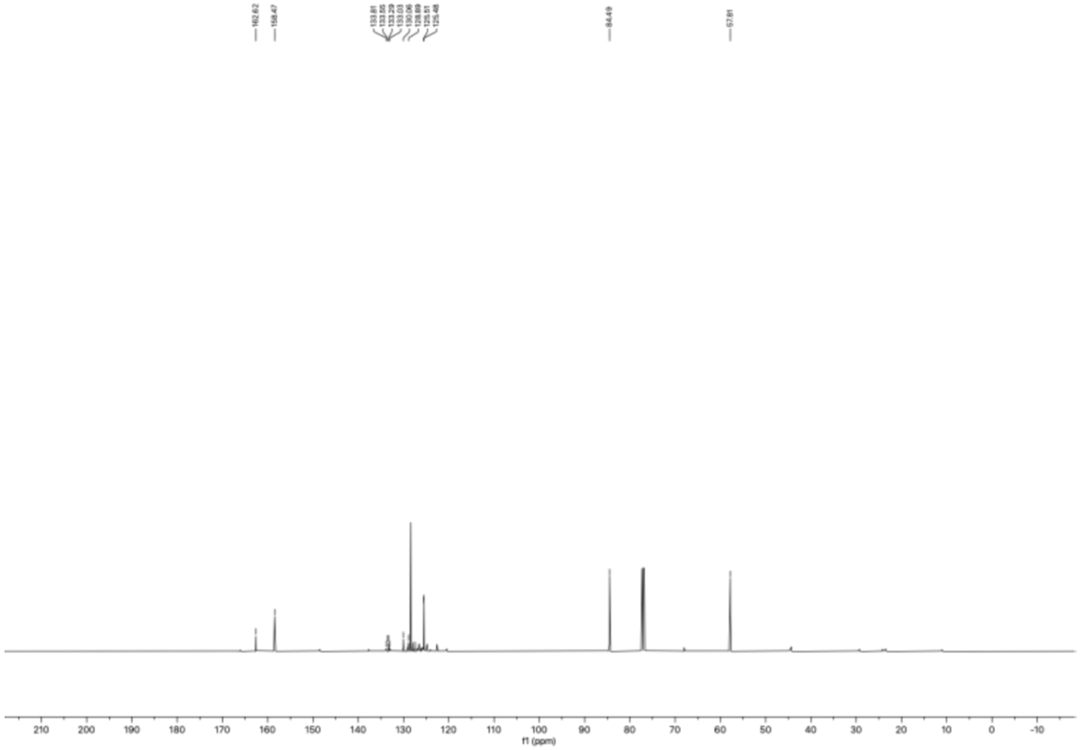

Figure S35. $^{13}C\{^{1}H\}$ NMR (125 MHz, $CDCl_3$) Spectrum of **12b**

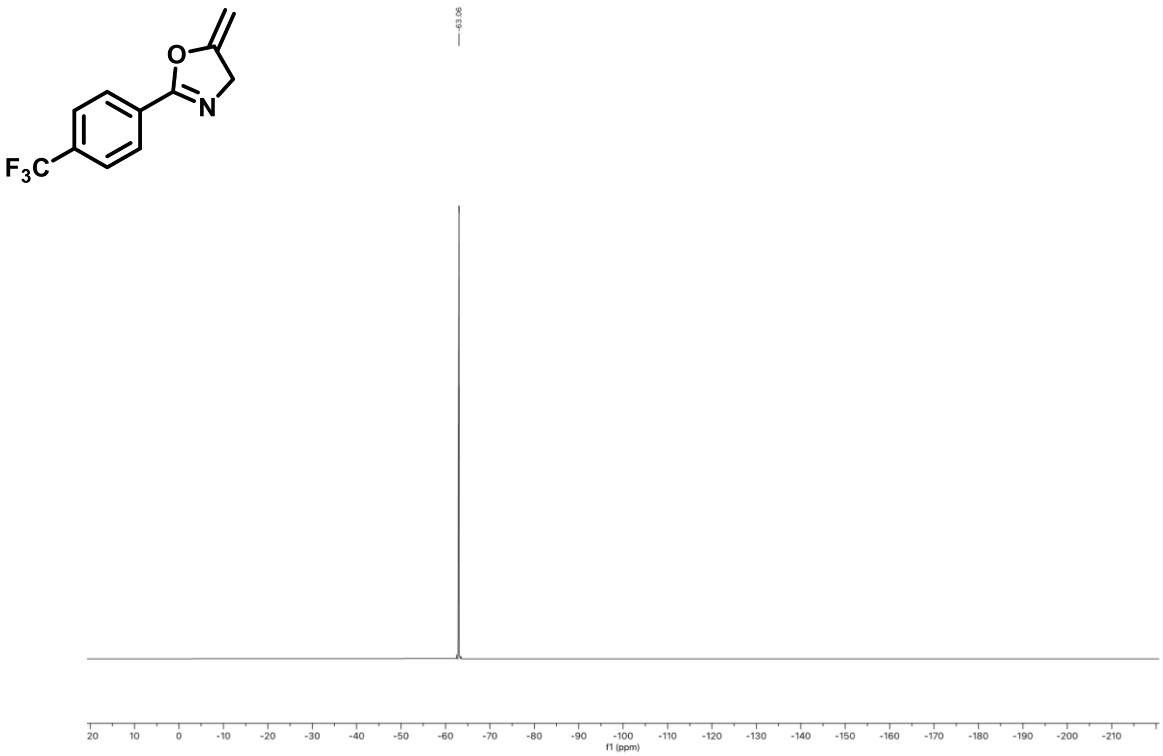

Figure S36. $^{19}F\{^{1}H\}$ NMR (471 MHz, $CDCl_3$) Spectrum of **12b**

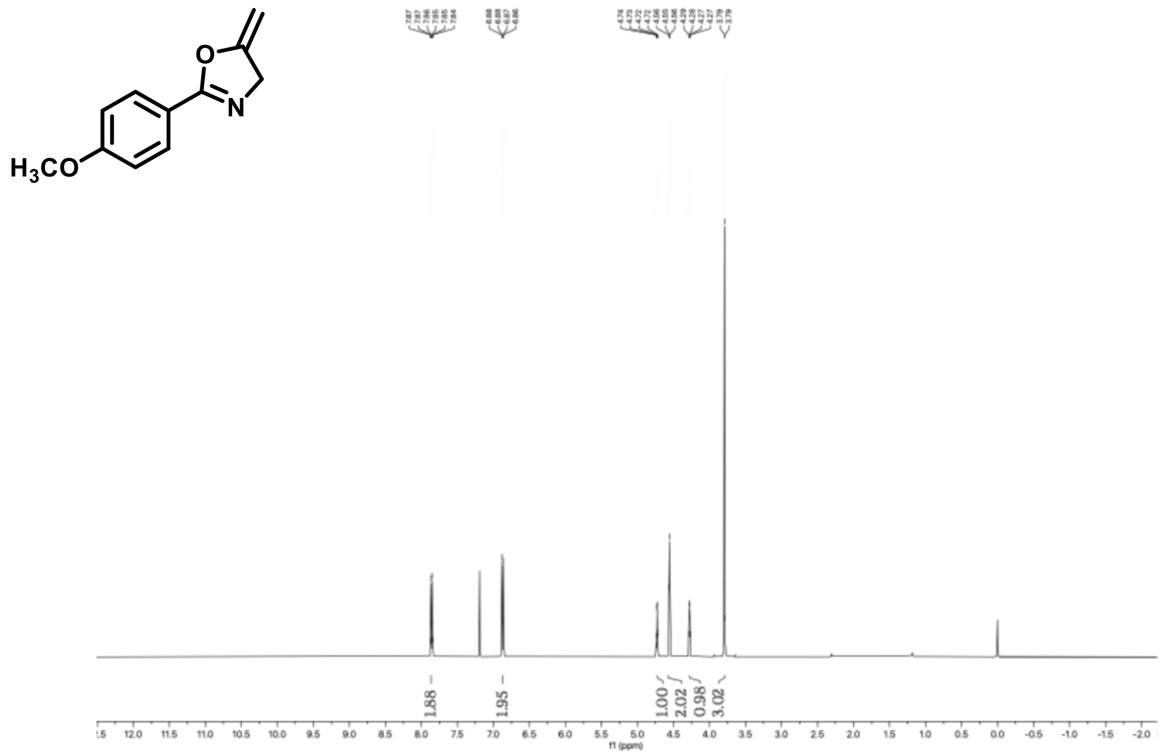

Figure S37. ^{1}H NMR (500 MHz, $CDCl_3$) Spectrum of **12c**

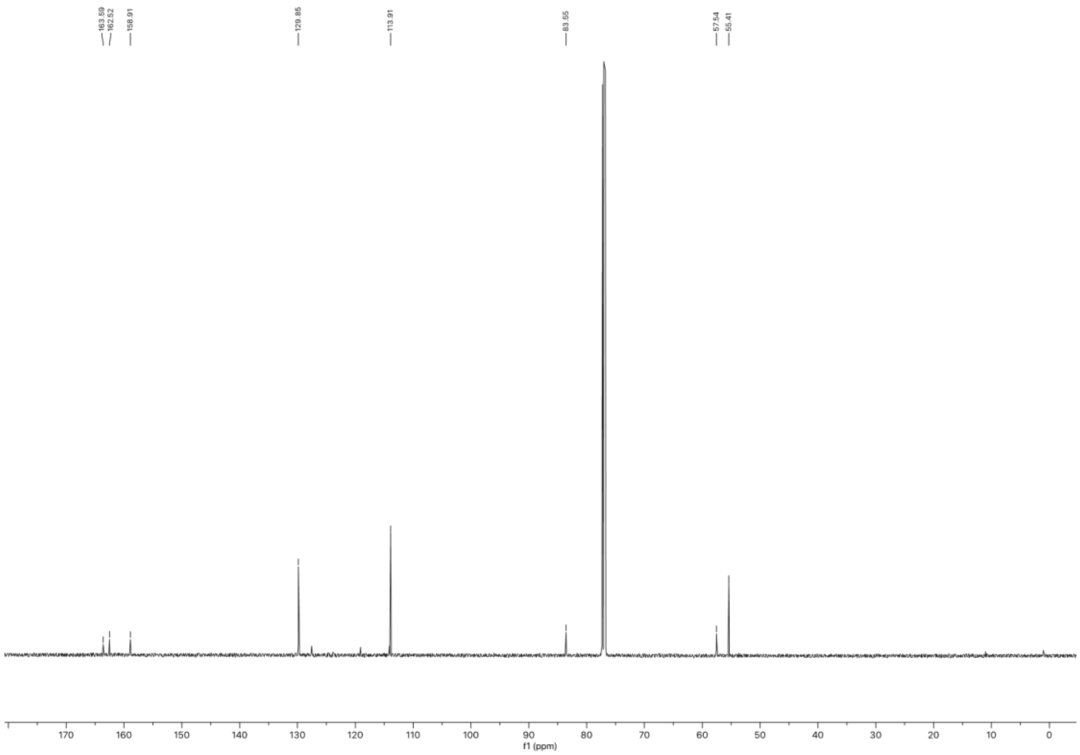

Figure S38. $^{13}C\{^{1}H\}$ NMR (125 MHz, $CDCl_3$) Spectrum of **12c**

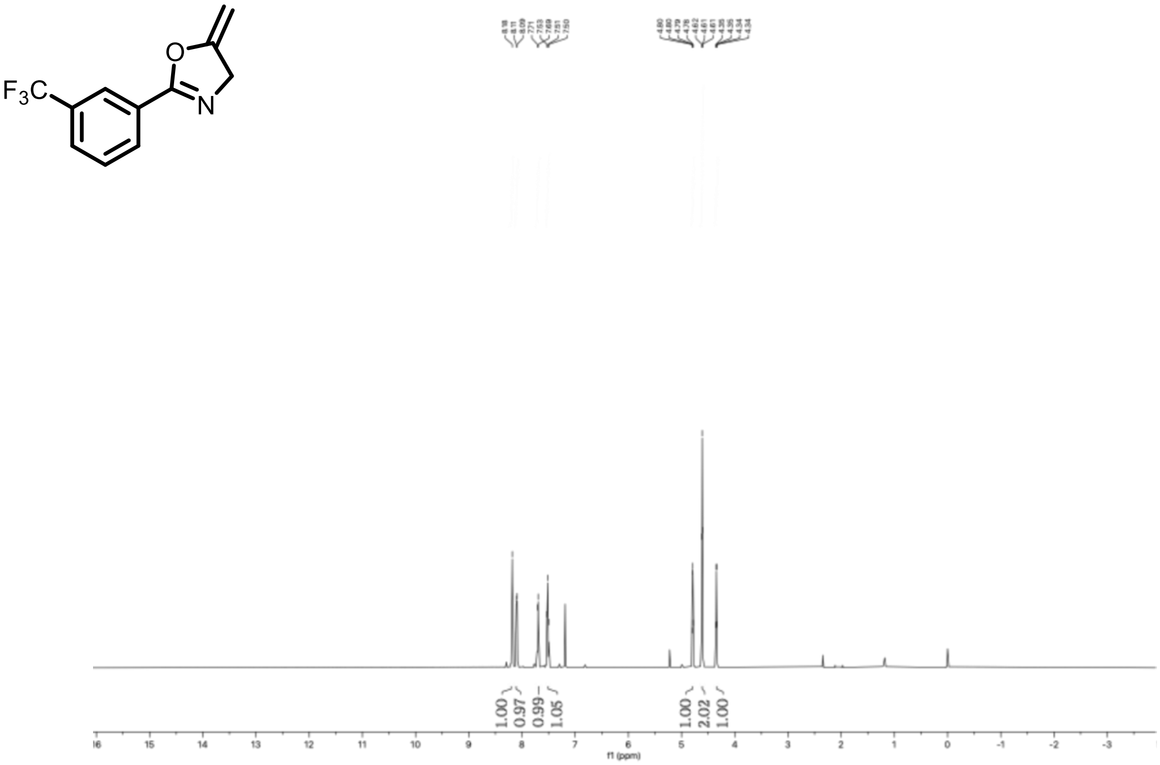

Figure S39. ^{1}H NMR (500 MHz, $CDCl_3$) Spectrum of **12d**

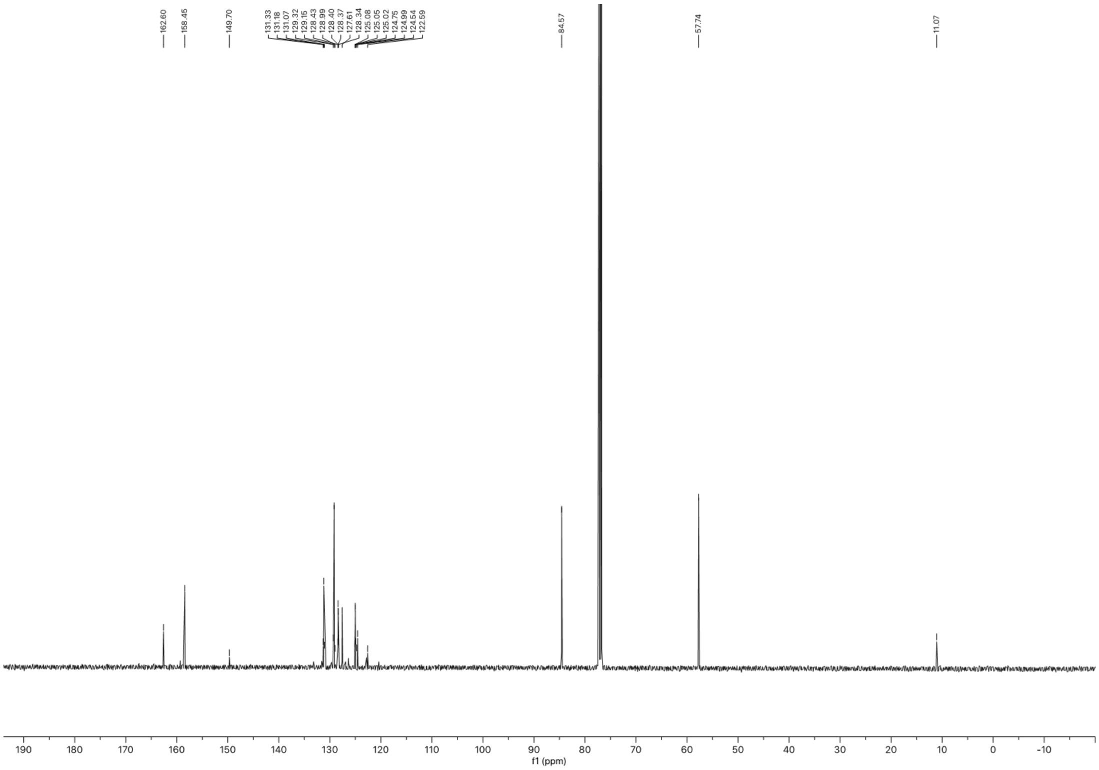

Figure S40. $^{13}C\{^{1}H\}$ NMR (125 MHz, $CDCl_3$) Spectrum of **12d**

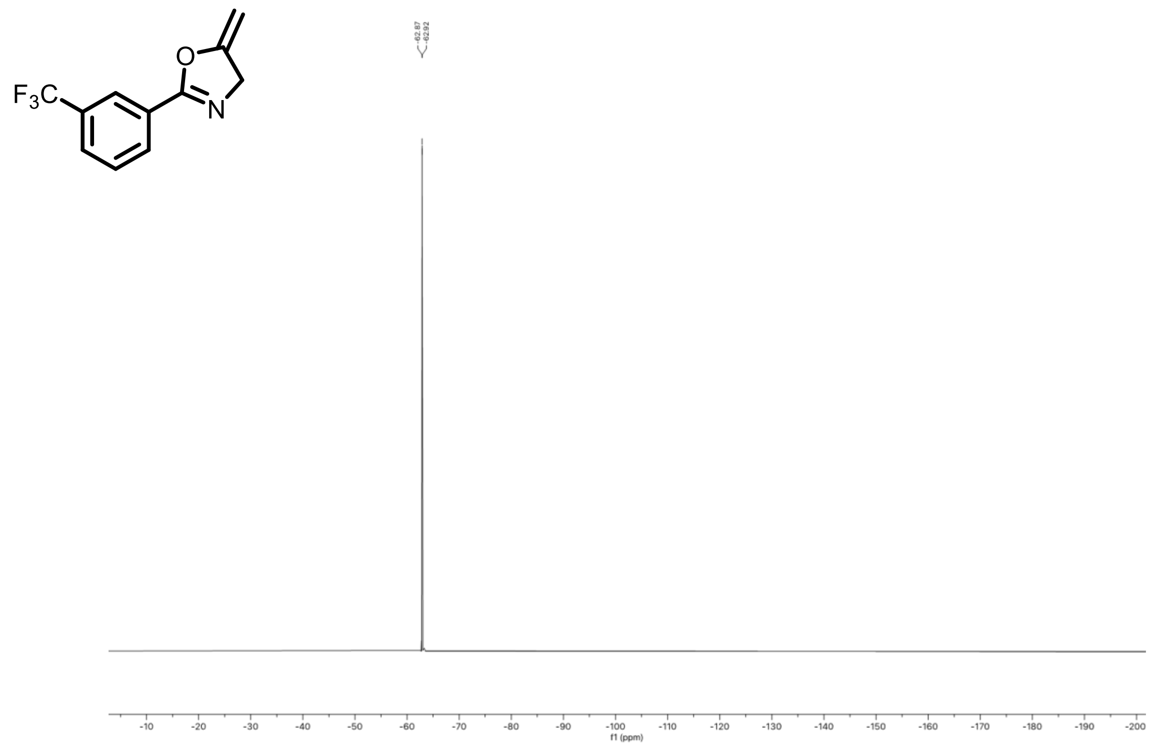

Figure S41. $^{19}F\{^{1}H\}$ NMR (471 MHz, $CDCl_3$) Spectrum of **12d**

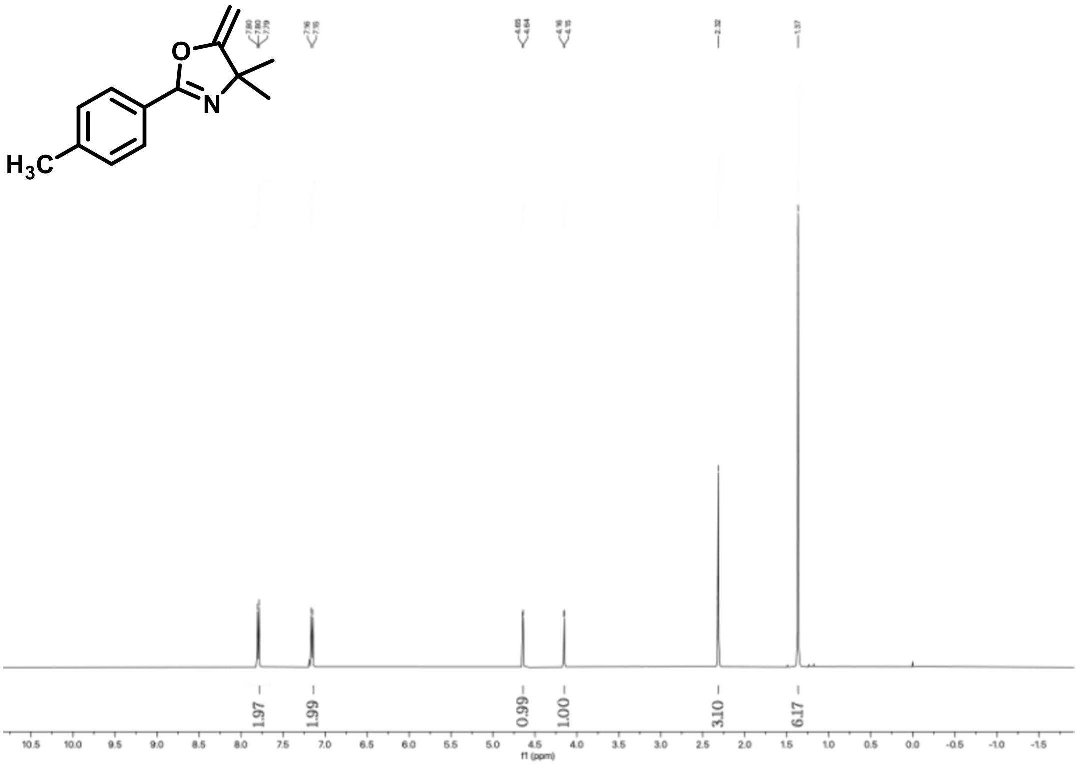

Figure S42. ^{1}H NMR (500 MHz, $CDCl_3$) Spectrum of **12e**

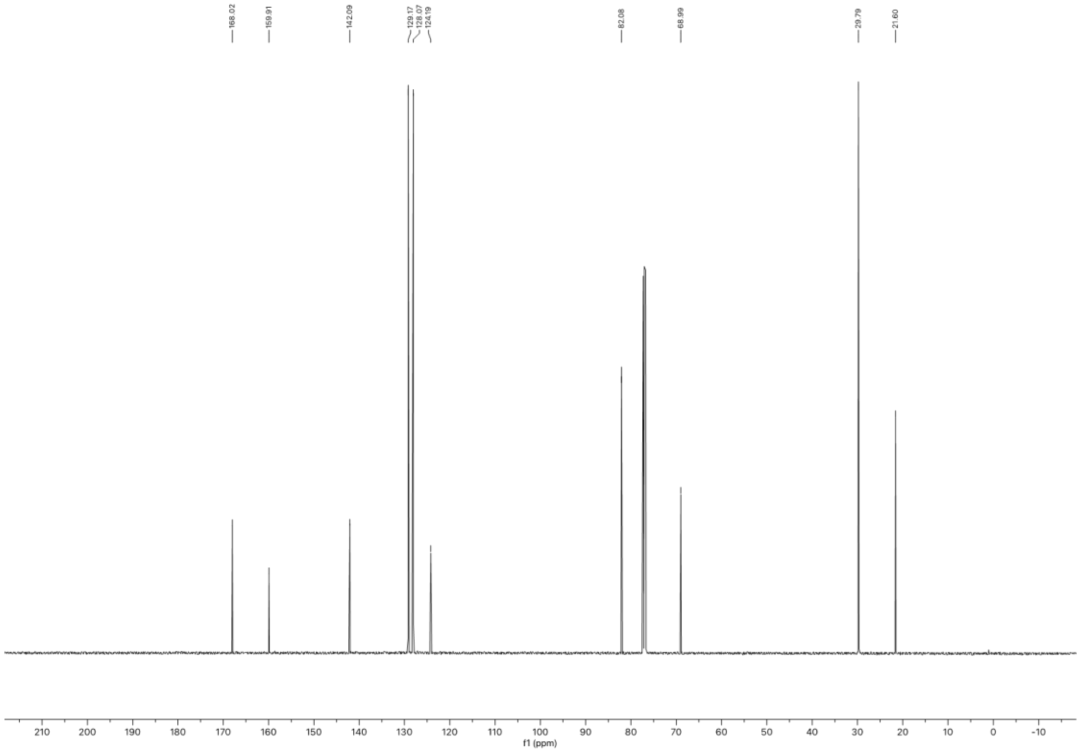

Figure S43. $^{13}C\{^{1}H\}$ NMR (125 MHz, $CDCl_3$) Spectrum of **12e**

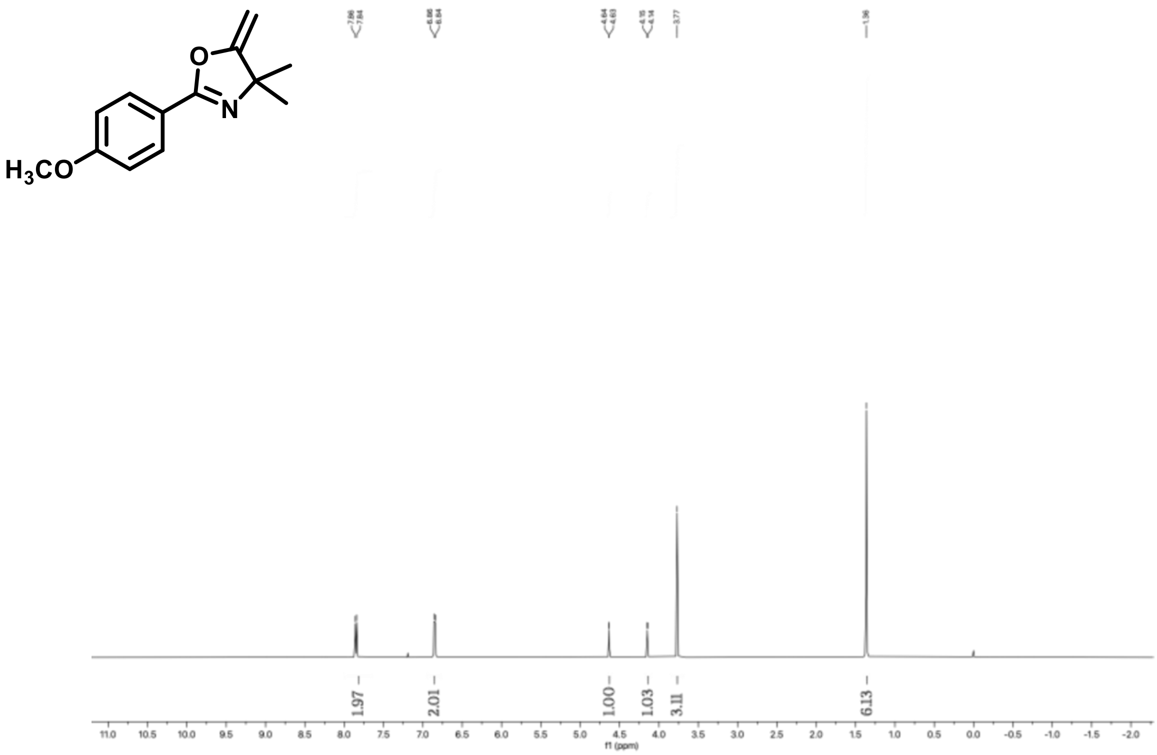

Figure S44. ^{1}H NMR (500 MHz, $CDCl_3$) Spectrum of **12f**

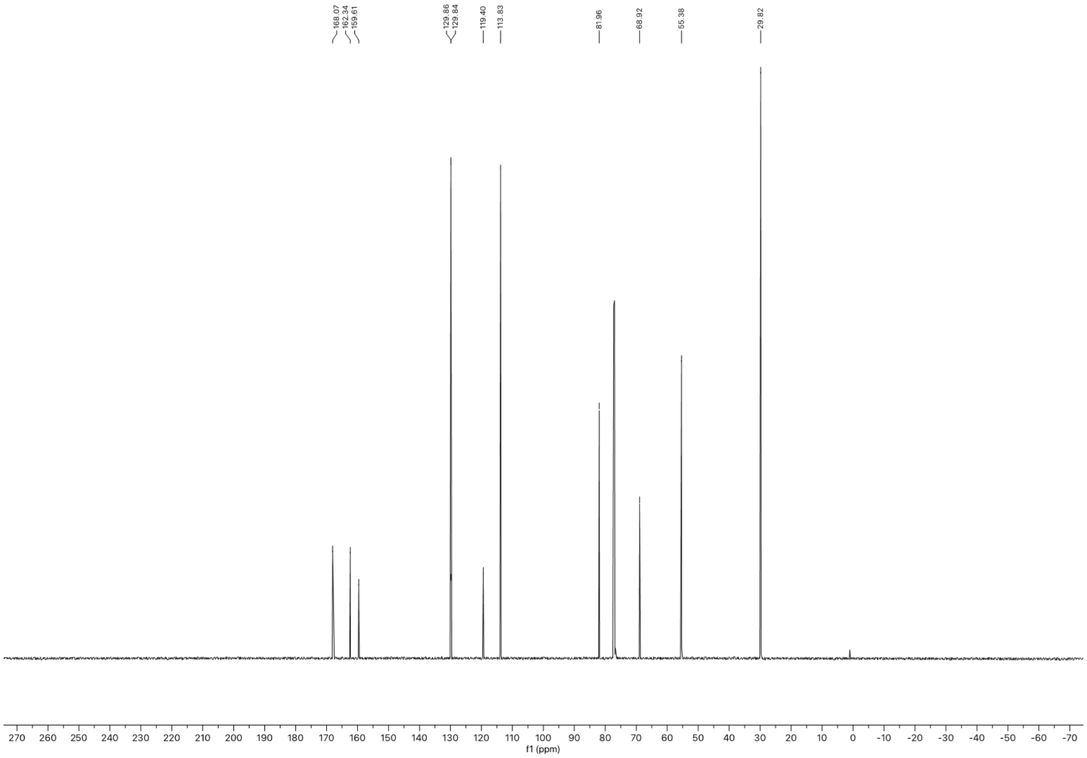

Figure S45. $^{13}C\{^{1}H\}$ NMR (125 MHz, $CDCl_3$) Spectrum of **12f**

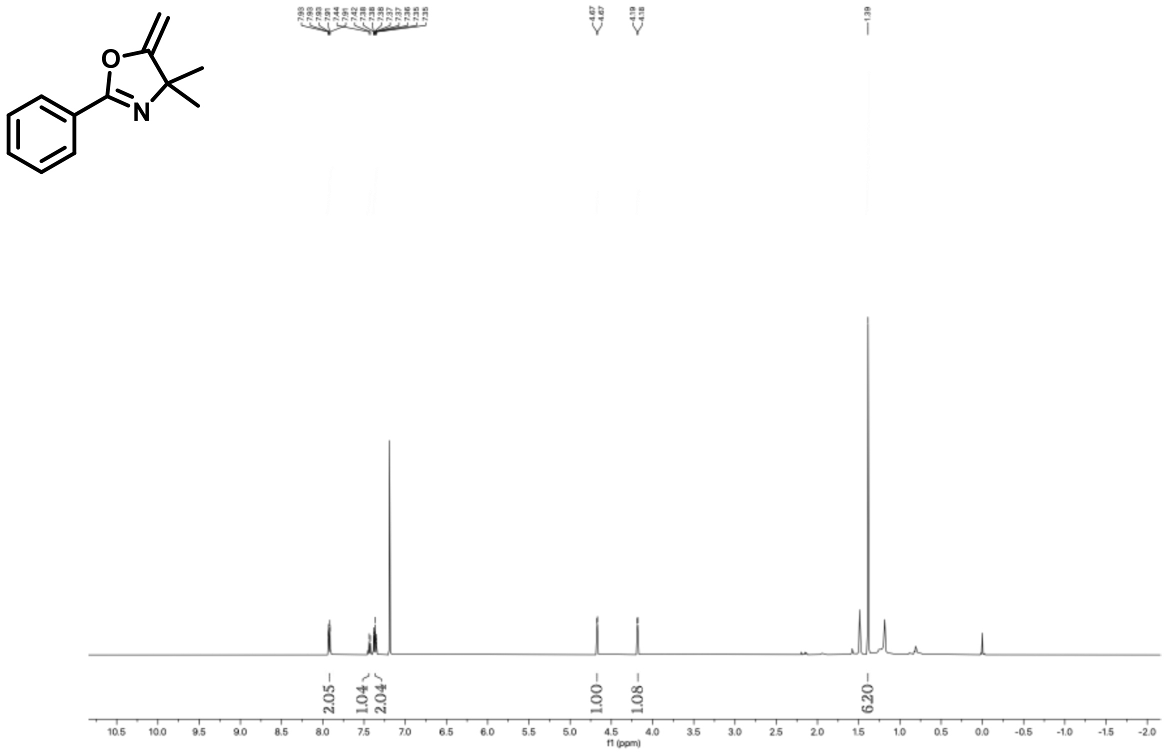

Figure S46. ^{1}H NMR (500 MHz, $CDCl_3$) Spectrum of **12g**

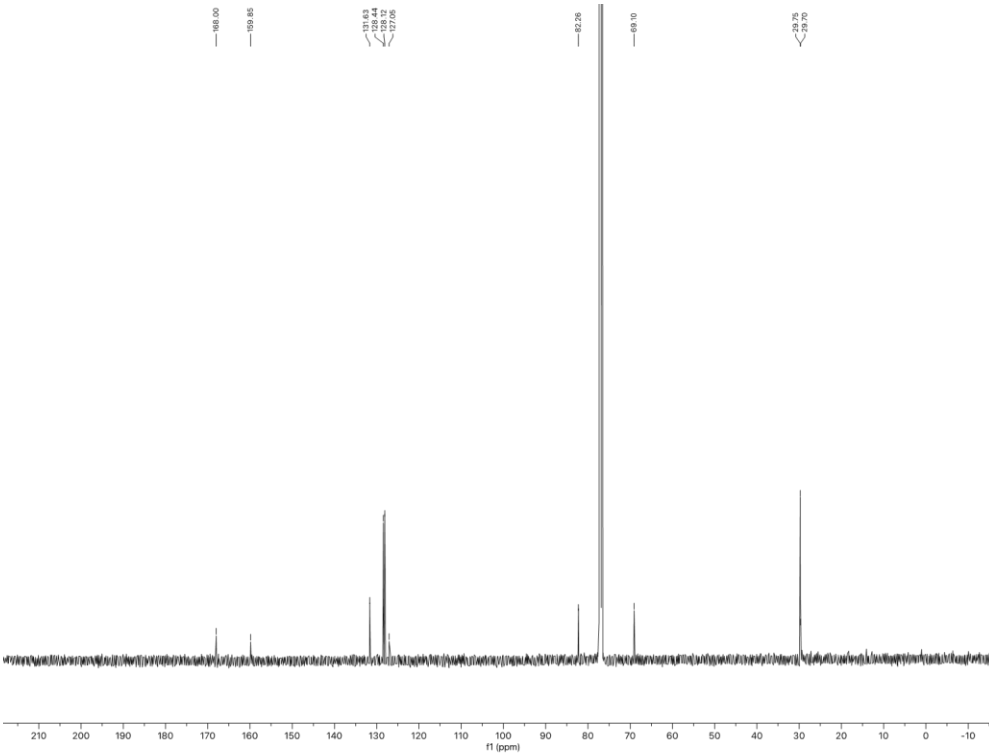

Figure S47. $^{13}C\{^{1}H\}$ NMR (125 MHz, $CDCl_3$) Spectrum of **12g**

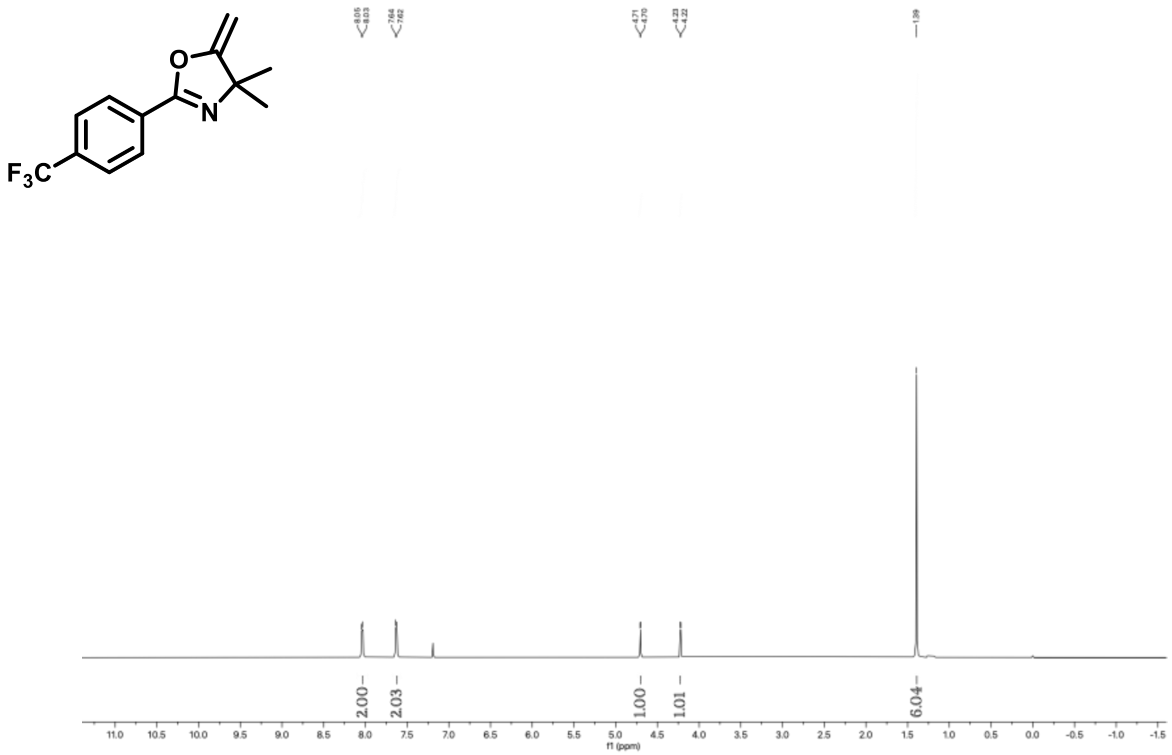

Figure S48. ^{1}H NMR (500 MHz, $CDCl_3$) Spectrum of **12h**

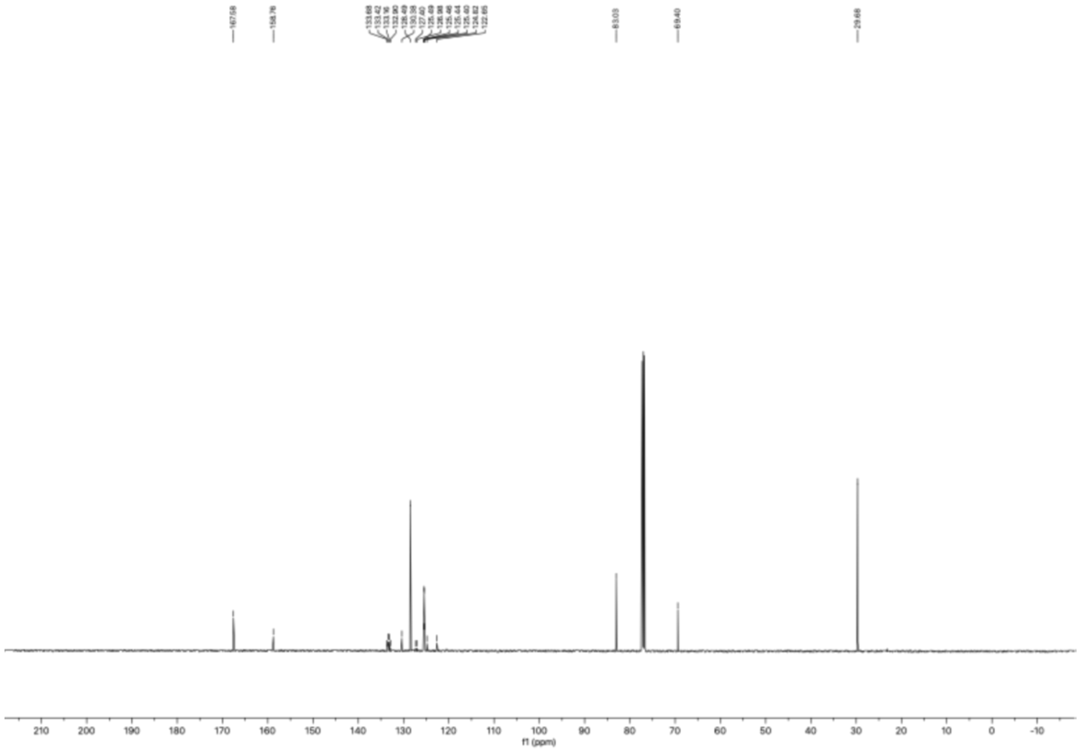

Figure S49. $^{13}C\{^{1}H\}$ NMR (125 MHz, $CDCl_3$) Spectrum of **12h**

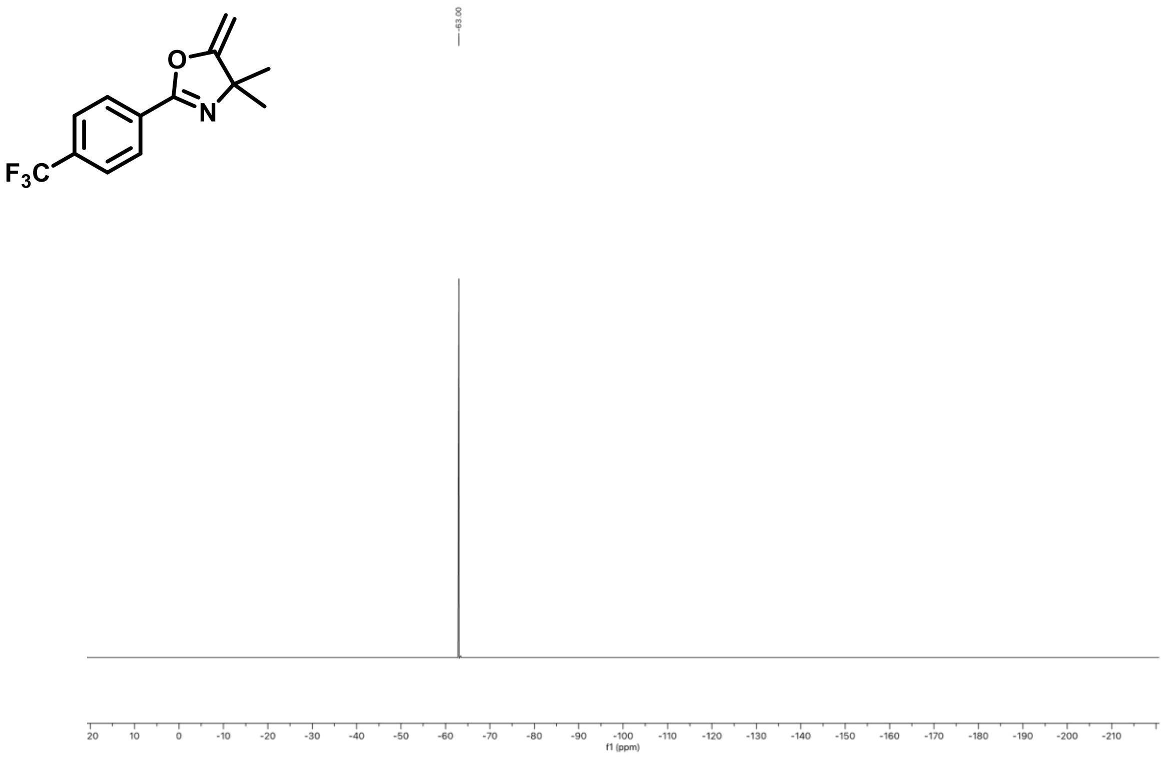

Figure S50. $^{19}F\{^{1}H\}$ NMR (471 MHz, $CDCl_3$) Spectrum of **12h**

References

[1] Park, D.-A.; Ryu, J. Y.; Lee, J.; Hong, S. Bifunctional N-Heterocyclic Carbene Ligands for Cu-Catalyzed Direct C–H Carboxylation with CO_2. *RSC Adv.* **2017**, *7*, 52496-52502.

[2] Zhou, T.; Gao, P.; Bisz, E.; Dziuk, B.; Lalancette, R.; Szostak, R.; Szostak, M. Well-defined, air- and moisture-stable palladium–imidazo[1,5-*a*]pyridin-3-ylidene complexes: a versatile catalyst platform for cross-coupling reactions by L-shaped NHC ligands. *Catal. Sci.Technol.* **2022**, *12*, 6581-6589.

[3] Xu, J. , Zhang, Y. , Yue, X. , Huo, J. , Xiong, D. , & Zhang, P. Selective oxidation of alkenes to carbonyls under mild conditions. *Green Chem.* **2021**, *23*, 5549-5555.

[4] Dunsford, J. J. , Cavell, K. J. , & Kariuki, B. M. Gold(I) complexes bearing sterically imposing, saturated six- and seven-membered expanded ring n-heterocyclic carbene ligands. *Organometallics* **2012**, *31*, 4118-4121.

[5] Wang, Tingting, Changmeng Liu, Dong Xu, Jiaxi Xu, and Zhanhui Yang. Iridium-Catalyzed and pH-Dependent Reductions of Nitroalkenes to Ketones. *Molecules* **2022**, *27*, 7822-7835.

[6] Seppnen, O., Aikonen, S. , Muuronen, M. , Alamillo-Ferrer, C. , J Burés, & Helaja, J. Dual H-bond activation of NHC–Au(I)–Cl complexes with amide functionalized side-arms assisted by H-bond donor substrates or acid additives. *Chem. Commun.* **2020**, *56*,14697-14700.

[7] Dong, K., Gurung, R. , Xu, X. , & Doyle, M. Enantioselective catalytic cyclopropanation-rearrangement approach to chiral spiroketals. *Org. Lett.* **2021**, *23*, 3955-3959.
